# Exosomes From Intestinal Epithelial Cells Promote Hepatic Differentiation of Liver Progenitor Cells in Gut‐Liver‐on‐a‐Chip Models

**DOI:** 10.1002/advs.202417478

**Published:** 2025-07-06

**Authors:** Liang Ye, Shi Li, Guofang Bi, Binghui Li, Zhai Cai, Meixian Jin, Ying Zhang, Wanren Yang, Yang Li, Shao Li, Wei Hu, Yi Gao, Mingxin Pan, Shuqin Zhou, Chao Zhang, Huichang Bi, Qing Peng

**Affiliations:** ^1^ Department of Hepatobiliary Surgery II General Surgery Center Zhujiang Hospital Southern Medical University Guangzhou Guangdong 510282 China; ^2^ Central Laboratory of the Second Affiliated Hospital, School of Medicine, The Chinese University of Hong Kong Shenzhen & Longgang District People's Hospital of Shenzhen Shenzhen 510282 China; ^3^ NMPA Key Laboratory for Research and Evaluation of Drug Metabolism & Guangdong Provincial Key Laboratory of New Drug Screening School of Pharmaceutical Sciences Southern Medical University Guangzhou 510282 China; ^4^ Department of General Surgery Zhujiang Hospital Southern Medical University Guangzhou 510000 China; ^5^ Anesthesiology Department of the Second Affiliated Hospital, School of Medicine, The Chinese University of Hong Kong Shenzhen & Longgang District People's Hospital of Shenzhen Shenzhen 518172 China; ^6^ Department of Anesthesiology Zhujiang Hospital Southern Medical University Guangzhou 510282 China; ^7^ Department of Oncology Zhujiang Hospital Southern Medical University Guangzhou 510282 China; ^8^ Institute of Regenerative Medicine Zhujiang Hospital Southern Medical University Guangzhou 510282 China

**Keywords:** exosomes, hepatic progenitor cells, intestinal epithelial cells, liver fibrosis, organ‐on‐a‐chip

## Abstract

Hepatic progenitor cells (HPCs) are frequently overactivated, and their differentiation into hepatocytes is impaired in advanced liver diseases. To explore the effects of intestinal epithelial cells and their exosomes on the hepatic differentiation of HPCs, co‐culture systems of Caco‐2/HepaRG cell lines and intestine/HPC organoids are established in a novel gut‐liver‐on‐a‐chip. Exosomes derived from intestinal organoids are administered to mice with carbon tetrachloride (CCL4)‐induced liver fibrosis. The results showed that the co‐culture of HPCs and intestinal epithelial cells promoted the hepatic differentiation of HPCs, mediated by exosomes derived from intestinal epithelial cells. Treatment with exosomes derived from intestinal organoids ameliorated liver fibrosis in a mouse model of CCL4‐induced liver fibrosis. A cluster of miRNAs, miR‐371‐373, is identified within the exosomes of the intestinal epithelial cells, which target RPS6KA2 to modulate hepatic differentiation. This findings demonstrate that exosomes from intestinal epithelial cells promote the hepatic differentiation of HPCs. Exosomes from intestinal organoids may be a novel therapeutic strategy for the treatment of advanced liver diseases.

## Graphical abstract

The effects of intestinal epithelial cells and their exosomes on the hepatic differentiation of HPCs are explored in a gut‐liver‐on‐a‐chip. Exosomes from intestinal epithelial cells improve liver fibrosis. Moreover, the miR‐371‐373 cluster in the exosomes may mediate this effect by regulating the hepatic differentiation of HPCs.

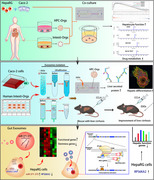

## 1 Introduction

The liver is a key organ that maintains metabolism and homeostasis. Acute or chronic liver injury has various aetiologies.^[^ ^1^ ^]^ ≈2 million people die of liver disease every year, and the number of patients with chronic liver disease continues to increase worldwide.^[^ ^2^ ^]^ The liver has regenerative capacity.^[^ ^3^ ^]^ In patients with mild liver injury, the damaged liver can be repaired via hepatocyte proliferation. However, when hepatocyte proliferation is impaired in severe liver injury, hepatocytes or cholangiocytes can dedifferentiate into hepatic progenitor cells (HPCs), which then proliferate and differentiate into hepatocytes and/or cholangiocytes.^[^ ^3^, ^4^ ^]^ When considering the prevalence of hepatocyte senescence and impaired liver regeneration observed in patients with chronic liver disease and cirrhosis, cholangiocytes as the primary source of HPCs may be a crucial repair mechanism in humans. This hypothesis was substantiated in a previous study.^[^ ^5^ ^]^ Despite the HPC‐mediated mechanism, liver regeneration appears to be inefficient in patients with advanced liver disease for whom the only therapeutic option is liver transplantation. HPC numbers in humans correlate with the severity of liver disease,^[^ ^6^ ^]^ implying that HPCs are robustly activated but inefficiently differentiate into mature hepatocytes in patients.^[^ ^7^, ^8^ ^]^ Therefore, the promotion of HPC‐to‐hepatocyte differentiation in order to generate functional hepatocytes may be a promising therapeutic approach for patients with advanced liver disease. To address this challenge, a better understanding of the mechanisms underlying the differentiation of HPCs into hepatocytes is required.

Previous studies have revealed that, in addition to the HPCs or hepatocytes themselves, the interaction of other non‐parenchymal cells in the liver is crucial in regard to regulating the differentiation of HPCs into hepatocytes.^[^ ^9^, ^10^, ^11^, ^12^ ^]^ However, the effect of extrahepatic microenvironmental factors on hepatocyte differentiation remains poorly understood. While recent studies have highlighted the cross‐talk between organs (e.g., kidney‐liver and lung‐liver interactions),^[^ ^13^, ^14^, ^15^, ^16^, ^17^ ^]^ no studies have yet examined the influence of other extrahepatic organs on the differentiation of hepatic progenitor cells. It is well known that the liver receives ≈70% of its blood supply from the portal vein, which is the direct outflow of the intestine.^[^ ^18^ ^]^ However, whether the intestine participates in maintaining hepatocyte function and regulating HPC development remains unclear. To address this question, it is crucial to build a suitable model that can accurately simulate the structural and anatomical relationship between the gut and liver. Both conditioned medium and co‐cultures in Transwell platforms have been widely used as in vitro models of organ communication in previous studies.^[^ ^19^, ^20^, ^21^, ^22^, ^23^, ^24^ ^]^ However, it cannot reproduce long‐term intercellular communication or effectively simulate complex and dynamic communication in the human body.^[^ ^25^ ^]^ Additionally, Transwell systems are not suitable for studies focusing on the direct effects of one organ on another. Recently, multiorgan‐on‐a‐chip microfluidic cell culture devices have been developed to model interactions between different organs and enable researchers to address some of the challenges mentioned above.^[^ ^25^, ^26^, ^27^ ^]^

**Figure 1:**
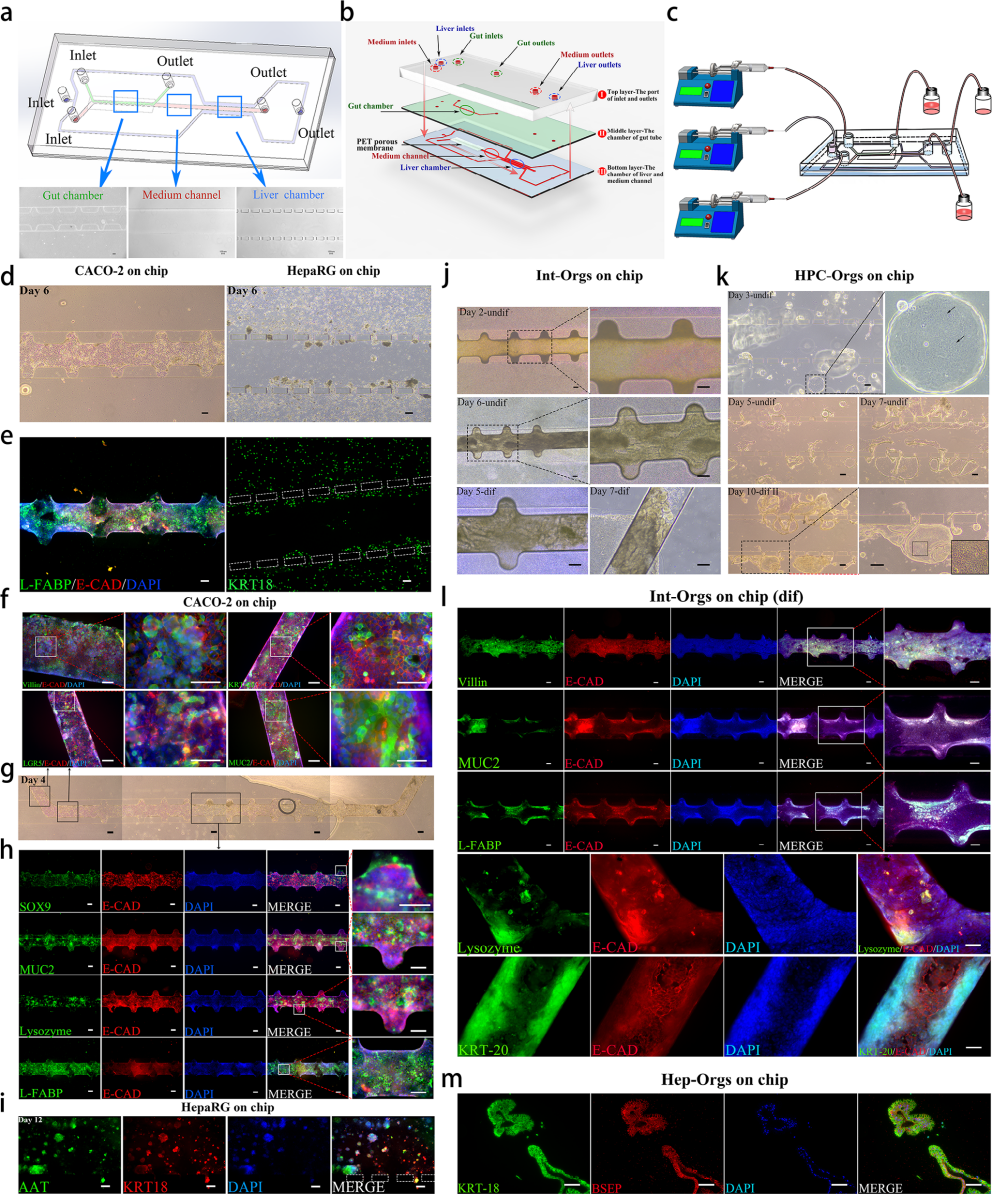
Establishment of an integrated gut‐liver‐on‐a‐chip with cell lines and organoids. a) The schema of the gut‐liver‐on‐a‐chip displays the upper left gut chamber (green), vessel‐like medium channel (red), and right bilateral liver chambers (blue). The bright field images of each area of the chip are shown below the schema, including the gut chamber (left), vessel‐like medium channel (middle), and liver chamber (right). Scale bar = 100 µm. b) Schematic diagram of the chip structure. c) Diagram of the assembled microfluidic device. d) Bright‐field images of intestinal epithelial cells (Caco‐2) and hepatic progenitor cells (HepaRG) cultured on the chip on Day 6. Scale bar = 100 µm. e) Left panel: Caco‐2 cells in the gut chamber were co‐stained with antibodies against E‐CAD (red), L‐FABP (green), and DAPI (blue) to show the nuclei. Right panel: HepaRG cells in the liver chamber were stained with antibodies against KRT18 (green). Scale bar = 100 µm. f) The Caco‐2 cells that grew near the inlet and outlet of the gut chamber were immunostained with antibodies against Villin, KRT20, LGR5, MUC2 (green), E‐CAD (red), and DAPI (blue) to show nuclei. Left panels: low magnification; right panels: high magnification. Scale bar = 100 µm. g) Full field of view: mini‐gut (Caco‐2 cells) on the chip cultured for 4 days, with four continuous bright‐field images. Scale bar = 100 µm. h) Images of Caco‐2 cells cultured for five days in the gut chamber. Immunostaining for intestinal cell markers: SOX9+ crypt stem cells (green), MUC2+ goblet cells (green), lysozyme+ Paneth cells (green), and L‐FABP+ enterocytes (green) were co‐stained with E‐CAD (red) and DAPI (blue). The rightmost images show enlarged views of the selected areas in the corresponding pictures immediately to the left. Scale bar = 100 µm. i) HepaRG cells cultured on the chip for 12 days were co‐stained with antibodies against AAT (green), KRT18 (red), and DAPI (blue). Scale bar = 100 µm. j) Bright‐field images of Int‐Orgs on the chip. Upper left panel and upper right panel: Int‐Orgs at the stage of maintenance perfusion I (Day 2) and high‐magnification field. Middle left and middle right panels: Int‐Orgs at stage of maintenance perfusion II (Day 6) and high‐magnification field. Lower left panel: Int‐Orgs after differentiation for 5 days. Lower right panel: Int‐Orgs after differentiation for 7 days. Scale bar = 100 µm. k) Bright‐field images of HPC‐Orgs on chip. Upper left panel: HPC‐Orgs on Day 3. Middle left and middle right panels: HPC‐Orgs at the stage of maintenance perfusion (Day 5 and Day 7). Lower left and lower right panels: Hep‐Orgs at differentiation stage II (Day 10) and high‐magnification field. Lower right corner: Hep‐Orgs at differentiation stage II (Day 10) with the morphological and structural details of the organoid cells in high magnification field. Scale bar = 100 µm. l) Immunofluorescence images of Int‐Orgs (differentiated) on the chip: Villin, MUC2, L‐FABP, lysozyme, and KRT‐20 (green), E‐CAD (red), and DAPI (blue). The three panels in the top right are magnified images corresponding to the square in the adjacent panels on the left. Scale bar = 100 µm. m) Immunofluorescence images of Hep‐Orgs on the chip: KRT‐18 (green), BSEP (red), and DAPI (blue). Scale bar = 100 µm.

It has been shown that organoids have several advantages over traditional 2D cell cultures. They accurately mimic the complexity and functionality of real organs, allowing more precise studies on disease mechanisms and drug testing.^[^ ^28^ ^]^ The 3D structure of organoids allows interactions between different cell types, leading to a more accurate modelling of cellular behaviour in vivo. Furthermore, organoids‐on‐a‐chip combines the advantages of both organoids and microfluidic chips and has become a new and promising form of a micro engineered model that recapitulates 3D tissue structure and physiology.^[^ ^29^ ^]^ To investigate the effect of the intestine on the differentiation of HPCs into hepatocytes, we established an integrated gut‐liver‐on‐a‐chip platform in our preliminary study. This two‐chamber chip was separated by a biocompatible porous membrane, allowing simultaneous culture of both intestinal and liver tissues. The specialized 3D villus structure of the intestine and the hepatic sinusoid of the liver can be recapitulated in each chamber of the chip (**Figure** **1**a). Gut‐liver coculture systems were constructed using either cell lines (Caco‐2 and HepaRG) or organoids (small intestine organoids (Int‐Orgs)) and liver organoids (Hep‐Orgs). on‐chip platforms. An initial study based on the gut‐liver‐on‐a‐chip platform showed that co‐culturing with intestinal tissue significantly promoted the differentiation of HPCs into hepatocytes.

Factors for inter‐organ information exchange are primarily transmitted via extracellular vesicles, among which exosomes constitute a significant proportion.^[^ ^30^ ^]^ Exosomes (EXOs) contain a wide range of signalling proteins, RNA, and genetic material that are transmitted to recipient cells through the bloodstream.^[^ ^31^ ^]^ EXOs have been proven to be a novel class of signalling structures mediating intercellular and interorgan communication.^[^ ^32^ ^]^ Based on these findings, we hypothesized that administration of EXOs from intestinal epithelial cells might be a key factor in promoting HPC‐to‐hepatocyte differentiation and could potentially improve advanced liver diseases. To validate this therapeutic effect, we conducted experiments using a CCL4‐induced liver injury and confirmed the beneficial effects of EXOs derived from Int‐Orgs. To further investigate the mechanisms by which intestinal epithelial cells promote the differentiation of HPCs into hepatocytes, we examined the role of EXOs from intestinal epithelial cells in HPCs differentiation and found that a cluster of miRNAs, miR‐371‐373 in exosomes from intestinal epithelial cells contributed to the enhancement of HPC‐to‐hepatocyte differentiation by targeting RPS6KA2. Our findings offer new insights for the development of innovative therapeutic strategies for patients with advanced liver disease.

## 2 Result

### 2.1 Construction of Integrated Gut‐Liver‐on‐a‐Chip Platforms Using Cell Lines and Organoids

The gut‐liver‐on‐a‐chip device was designed with three layers of PDMS: a top layer for the inlets and outlets, a middle layer for intestinal culture, and a basal layer for liver culture (Figure 1a,b; Figure S1a, Supporting Information). The upper and lower chambers were separated by a porous polyester membrane (10 µm thickness and 0.4 µm pore size). The intestinal culture was placed in the left upper chamber with a bionic spatial distribution that mimicked the structure of the intestinal crypt‐ and villus‐like domains (Figure 1a). The epithelium of the small intestine predominantly consists of absorptive cells. The circular folds, villi, and microvilli work in concert to significantly expand the intestinal surface area, thereby ensuring maximal contact between the epithelial cells and chyme, which is crucial for optimal nutrient absorption.^[^ ^33^, ^34^ ^]^ In this device, the intestinal culture region featured a bionic spatial distribution that closely mimicked the structural organization of the intestinal crypt‐ and villus‐like domains (Figure 1a). The liver culture region recapitulated the microstructure of the hepatic sinusoid with liver chambers arranged on both sides and a medium‐flow channel designed through the middle (Figure 1a). Micropillars were created as a barrier between the cells and the medium flow. The central microtube was designed as a vessel‐like structure connecting the intestine and liver chambers (Figure 1a).

Caco‐2 and HepaRG cells were seeded on the chip in the intestinal and liver chambers, respectively, and perfused with medium using a syringe pump (Figure 1c; Figure S1b, Supporting Information). After continuous culturing for 6 days, the bright‐field image on the chip showed that Caco‐2 cells in the gut region formed a tight cellular barrier along the villus‐like structure within the channel, while HepaRG cells in the liver region were distributed along both sides of the hepatic sinusoidal region (Figure 1d). The respective coculture media for the cell lines were determined in preliminary studies (Figure S1c,d, Supporting Information). Immunostaining showed that under dynamic perfusion culture conditions, the cells cultured in the intestinal chamber on the chip exhibited a bionic spatial distribution similar to that of the villi of the small intestine in vivo (Figure 1e‐left). LGR5/KRT20‐positive cells were observed in the gut chamber (Figure 1f). Both the central region and crypt‐like structures flanking the channel contained cells that were positively stained for SOX9 (intestinal stem cells) and MUC2 (goblet cells), whereas lysozyme‐positive Paneth cells and L‐FABP‐positive enterocytes were found to be more densely concentrated in the central channel (Figure 1g,h). Positive markers (SOX9, L‐FABP, lysozyme, and MUC2) for functional cells were highly expressed throughout the culture channel of Caco‐2 cells when compared to those cultured under 2D static culture conditions (Figure S1e, f, Supporting Information). HepaRG cells cultured in the liver chamber formed spheroids with diameters ranging from 30 to 100 µm after 12 days (Figure S1g, Supporting Information). The expression of hepatocyte markers (ALB, AAT, and KRT18) in the HepaRG cells on the chip was confirmed by immunofluorescence staining (Figure 1e,i; Figure S1h, Supporting Information).

In this study, two types of organoids were generated from adult human stem cells (intestinal crypt stem cells and hepatic progenitor cells) (Figure S2a—e, Supporting Information). The culture strategies for both intestinal and liver organoids involved the pre‐ and post‐differentiation stages, as illustrated in Figure S2a (Supporting Information). Undifferentiated Int‐Orgs grew in a grape‐like cluster pattern, while HPC‐Orgs grew in a cystic shape (Figure S2b, c, Supporting Information). Compared with the organoids before differentiation, the cell arrangement of the differentiated Int‐Orgs and Hep‐Orgs was more compact and darker (Figure S2d, e, Supporting Information). Morphologically, the budding structures of Int‐Orgs were more obvious, while Hep‐Orgs still maintained a cystic structure (Figure S2f, g, Supporting Information). Quantitative real‐time polymerase chain reaction (qRT‐PCR) results showed that HPCs prior to differentiation highly expressed bile duct cell markers (*KRT19*, *HNF1β*, *CFTR*, *EPCAM*), while HPCs after hepatic differentiation highly expressed hepatocyte markers (*ALB*, *HNF4A*, *KRT18*, *CYP3A4*) (Figure S3a, b, Supporting Information). Compared with undifferentiated HPCs, the expression of hepatocyte markers in differentiated HPCs more closely resembled that in primary human hepatocytes (Figure S2a, b, Supporting Information). Immunostaining results confirmed the stemness and bipotent transitional capabilities of HPC‐Orgs, as evidenced by the expression of the stemness marker SOX9, bile duct marker EPCAM/CK19, and hepatocyte marker HNF4A (Figure S3c, Supporting Information). After two‐stage differentiation, the differentiated liver organoids (Hep‐Orgs) displayed strong expression of hepatocyte markers (ALB, HNF4A, KRT18, CYP3A4), hepatocyte functional proteins (ALB and AAT), and hepatocyte polar proteins (DPPIV, ZO‐1, BSEP, MDR1) (Figure S3d, Supporting Information). In comparison with HPC‐Orgs, the levels of ALB and AAT in Hep‐Orgs were elevated, while the secretion of AFP decreased significantly (Figure S3e, Supporting Information). In both pre‐ and post‐differentiated Int‐Orgs, lysozyme‐positive Paneth cells, MUC2‐positive goblet cells, ChgA‐positive enteroendocrine cells, L‐FABP‐positive enterocytes, and the ubiquitous villin protein were detected by immunostaining. The expression of these intestinal cell markers was more obvious and prominent in post‐differentiated Int‐Orgs (Figure S3f, g, Supporting Information).

Subsequently, the undifferentiated Int‐Orgs and HPC‐Orgs were seeded in their respective culture chambers on the chip. On Day 2, Int‐Orgs were distributed in crypt‐like structures. By Day 6, Int‐Orgs had grown thicker and covered almost the entire channel of the intestinal chamber (Figure 1j). The intestinal barrier constructed by differentiated Int‐Orgs on the chip had a lower permeability ratio of FITC‐dextran than the Caco‐2 cell‐based intestinal barrier, indicating better intestinal barrier integrity for the organoids‐on‐a‐chip (Figure S4a, Supporting Information). After a short culture period of 2–3 days, the typical organoid morphology of HPC‐Orgs was observed in the liver chamber (Figure 1k). Following differentiation, the morphology of the Hep‐Orgs on the chip underwent changes, with more hepatocyte‐like cells and increased cytoplasmic granularity (Figure 1k). The expression levels of E‐CAD (adherent junctions) and Villin in differentiated Int‐Orgs on the chip were higher and denser than those in Caco‐2 cells on the chip. In addition, the expression of other intestinal cell markers (MUC2, L‐FABP, KRT‐20, and Lysozyme) was detected in the corresponding regions within the intestinal chamber (Figure 1l). For Hep‐Orgs, expression levels of BSEP and KRT‐18 were detected after differentiation (Figure 1m). Moreover, the levels of secreted proteins (ALB and AAT) from both Hep‐Orgs and HepaRG cells were found to be higher on the chip than those observed under Transwell culture conditions (Figure S4b,c, Supporting Information).

### 2.2 Coculture of HPCs with Intestinal Cells Promoted Hepatic Differentiation in the Gut‐Liver‐on‐a‐Chip Models

**Figure 2:**
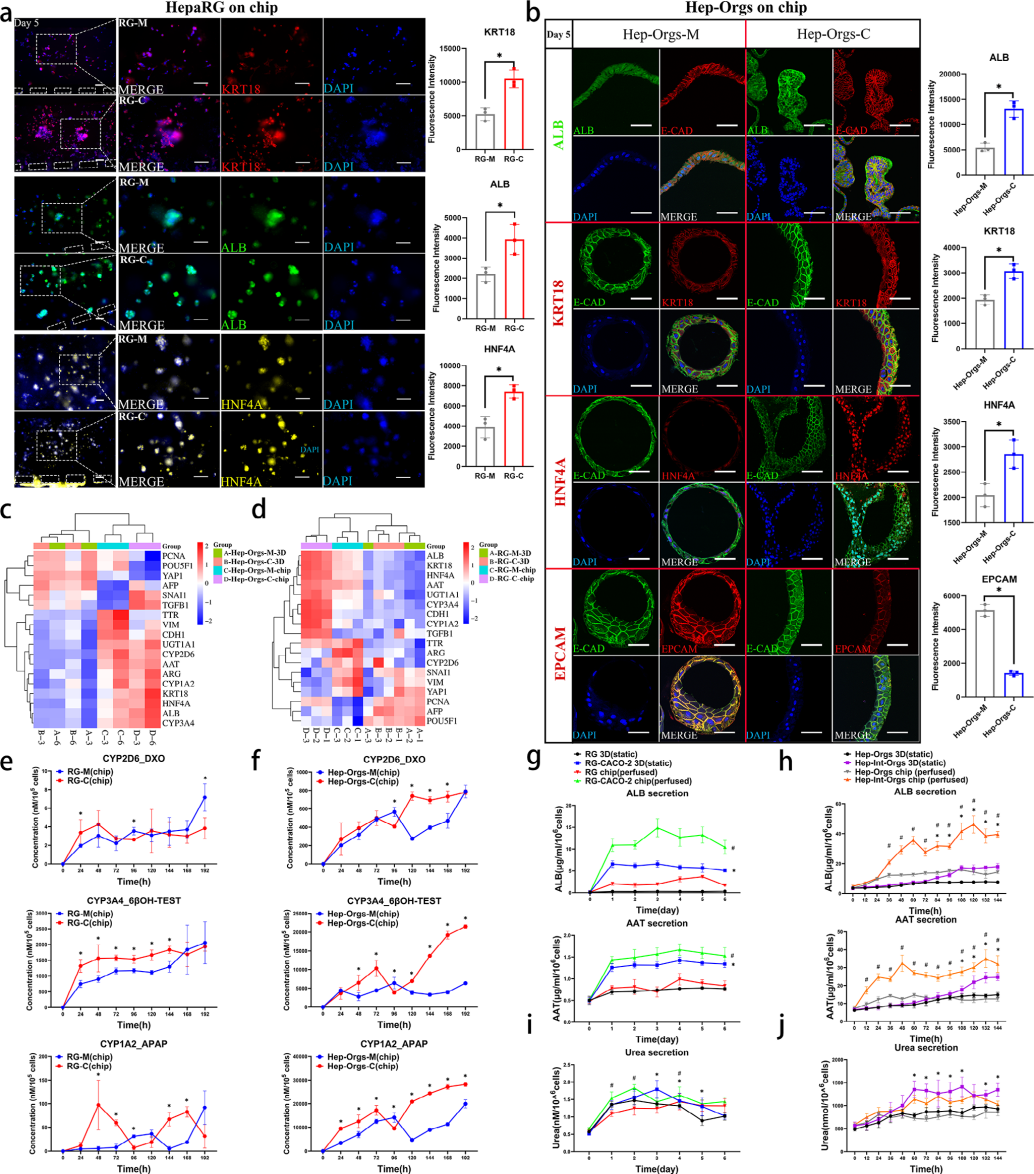
Enhanced hepatic differentiation of HPCs (HepaRG/HPC‐Orgs) cocultured with intestinal cells (Caco‐2/Int‐Orgs) on the chip. a) Caco‐2 and HepaRG cells were cocultured on the chip for 5 days. Immunostaining was used to compare the protein levels between the RG‐M (HepaRG monoculture) and RG‐C (HepaRG cocultured with Caco‐2) groups on the chip. Red: KRT‐18, green: ALB, yellow: HNF4A, blue: DAPI. Scale bar = 100 µm. Right‐side charts show the fluorescence intensity of the labelled proteins in the left‐side panel (*n* = 3, mean ± SD, Student's *t*‐tests). ^*^ *p* < 0.05. b) Immunofluorescence staining of the markers of hepatic differentiation in mono‐ and cocultured Hep‐Orgs on the chip. Hep‐Orgs on chip‐M: Hep‐Orgs monocultured on chip at differentiation stage II (Day 5); Hep‐Orgs on chip‐C: Hep‐Orgs cocultured with Int‐Orgs on the chip at differentiation stage II (Day 5). ALB (green), KRT‐18 (red), HNF4A (red), EPCAM (red), E‐CAD (red or green), and DAPI (blue) are shown in the left panel. Scale bar = 100 µm. The right‐side charts show the fluorescence intensity of the detected proteins (*n* = 3, mean ± SD, Student's *t*‐tests). ^*^ *p* < 0.05. c) Expression of genes related to hepatic differentiation and stemness in Hep‐Orgs cultured in Transwell plates (3D) and the chip was measured by qRT‒PCR. A: Hep‐Orgs‐M‐3D, Hep‐Orgs monocultured in the Transwell 3D system. B: Hep‐Orgs‐C‐3D, Hep‐Orgs cocultured with Int‐Orgs in the Transwell 3D system. C: Hep‐Orgs‐M‐chip, Hep‐Orgs monocultured on chip. D: Hep‐Orgs‐C‐chip, Hep‐Orgs cocultured with Int‐Orgs on chip; 3: after two‐stage differentiation for 3 days; 6: after two‐stage differentiation for 6 days. The heatmap displays the log2 (fold change) value of the genes. Correlation analysis was conducted according to the gene expression of each group *(n* = 3). d) Expression of genes related to hepatic differentiation and stemness in HepaRG cells in Transwell (3D) and the chip was detected by qRT‐PCR. A: RG‐M‐3D, HepaRG monocultured in Transwell 3D system; B: RG‐C‐3D, HepaRG cocultured with Caco‐2 cells in Transwell 3D system; C: RG‐M‐chip, HepaRG monocultured on chip; D: RG‐C‐chip, HepaRG cocultured with Caco‐2 cells on chip. 1, 2, and 3 represent the day(s) of coculture. The heat map displays the log2 (fold change) value of the genes. Correlation analysis was conducted according to the gene expression trend of each group (*n* = 3). e,f) Measurement of CYP metabolites: DXO (CYP2D6), 6βOH‐TEST (CYP3A4), and APAP (CYP1A2) in HepaRG cells (e) and Hep‐Orgs (f) mono‐ and coculture groups on chip within 8 days (*n* = 3, mean ± SD, Two‐way ANOVA). ^*^ *p* < 0.05. g) ELISA data of the levels of secreted ALB and AAT in the HepaRG cells monocultured and cocultured in the Transwell‐3D system and chip in 6 days (*n* = 3, mean ± SD, Two‐way ANOVA). ^*^ *p* < 0.05 in RG‐Caco‐2 3D(static) versus RG 3D(static), ^#^ *p* < 0.05 in RG‐Caco‐2 chip(perfused) versus RG chip(perfused). h) ELISA data of the levels of secreted ALB and AAT in Hep‐Orgs monocultured and cocultured in the Transwell‐3D system and chip in 6 days (*n* = 3, mean ± SD, Two‐way ANOVA). ^*^ *p* < 0.05 in Hep‐Int‐Orgs 3D(static) versus Hep‐Orgs 3D(static), ^#^ *p* < 0.05 in Hep‐Int‐Orgs chip(perfused) versus Hep‐Orgs chip(perfused). i) Urea synthesis analysis of HepaRG cells monocultured and cocultured in Transwell and chip detected by colorimetry within 6 days (*n* = 3, mean ± SD, Two‐way ANOVA). ^*^ *p* < 0.05 in RG‐Caco‐2 3D(static) versus RG 3D(static), ^#^ *p* < 0.05 in RG‐Caco‐2 chip(perfused) versus RG chip(perfused). j) Urea synthesis analysis of Hep‐Orgs monocultured and cocultured in Transwell and chip, detected by colorimetry within 6 days (*n* = 3, mean ± SD, Two‐way ANOVA). ^*^ *p* < 0.05 in Hep‐Int‐Orgs 3D(static) versus Hep‐Orgs 3D(static), ^#^ *p* < 0.05 in Hep‐Int‐Orgs chip(perfused) versus Hep‐Orgs chip(perfused).

In order to investigate the effect of intestinal cells (Caco‐2 or Int‐Orgs) on hepatic progenitor cells (HepaRG/HPC‐Orgs), a coculture system was established on a chip. A schematic of the experimental procedure for organoid coculture and differentiation on the chip is shown in Figure S5a (Supporting Information). The coculture media for organoids were optimized prior to the establishment of gut‐liver‐organoids‐on‐chip (Figure S5b—h, Supporting Information). Fluorescence imaging of the chip showed that the expression of ALB, HNF4A, and KRT18 in HepaRG cells co‐cultured with Caco‐2 cells on day 5 was significantly higher than that in HepaRG cells cultured alone (**Figure** **2**a). In the liver culture region, hepatocytes located near the central area of the liver sinusoids exhibited more pronounced expression of ALB and AAT than those in the peripheral regions of the liver sinusoids (Figure S6a, Supporting Information). This was likely due to the fact that cells in the former regions could more fully and rapidly receive the secreted substances produced by the intestinal cells. After sectioning the paraffin‐embedded Hep‐Orgs, various cross‐sections of cyst‐like structures were observed (Figure 2b). Increased expression of ALB, HNF4A, and KRT18 was also detected in co‐cultured Hep‐Orgs (ALB‐2.5 fold, HNF4A‐1.4 fold, KRT18‐1.5 fold) (Figure 2b). qRT‐PCR results showed that the expression of *ALB*, *CYP3A4*, *HNF4A*, *KRT18*, *CYP1A2*, *AAT*, *CDH1*, and *UGT1A1* were upregulated in both co‐cultured HepaRG and HPC‐Orgs, whereas genes associated with proliferation and stemness (*PCNA* and *POU5F1*) exhibited a downward trend in these cells (Figure 2c,d). The level of the CYP3A4 enzyme in HepaRG cells in the co‐culture group was significantly increased (Figure S6b, Supporting Information). To evaluate the activity of the drug‐metabolizing enzymes of CYP450s in hepatocytes under different conditions, dextromethorphan, testosterone, and phenacetin were added, and their corresponding metabolites were analyzed using HPLC‐MS. After subtracting the levels of metabolites solely produced by Caco‐2 or Int‐Orgs alone, the results of HPLC‐MS analysis indicated that, under coculture conditions, the concentrations of DXO (Dextrophan), 6βOH‐TEST (6β‐hydroxy testosterone), and APAP (acetaminophen)—the metabolic products of dextromethorphan, testosterone, and phenacetin via CYP2D6, CYP3A4, and CYP1A2, respectively—were elevated compared to those observed under monoculture conditions (Figure 2e, f; Figure S6c, d, Supporting Information). This difference was found to be particularly pronounced in co‐cultured Hep‐Orgs groups (Figure 2f). ELISA data revealed that the levels of secreted proteins (ALB and AAT) were enhanced in co‐cultured HepaRG cells and Hep‐Orgs compared to those in the monocultured groups (Figure 2g,h). Urea production was also significantly elevated in co‐cultured Hep‐Orgs (1.8‐fold), whereas no significant increase was observed with HepaRG cells (Figure 2i,j). To further investigate the potential mechanisms regulating the differentiation of HPCs into intestinal epithelial cells, transcriptome sequencing of HepaRG cells and Hep‐Orgs on chips was performed. Compared with the monoculture group, HepaRG cells in the coculture group had 2100 upregulated and 2124 downregulated genes (Figure S7a, Supporting Information). Similarly, in Hep‐Orgs, 2106 genes were upregulated, and 2113 genes were downregulated in the coculture group (Figure S7b, Supporting Information). There was an overlap of 1159 DEGs between the HepaRG and Hep‐Org groups (Figure S7c, Supporting Information). Liver function‐related genes, such as *ALB*, *AAT*, *HNF4A*, *E‐CAD*, and *CYP3A4*, were found to be highly expressed in both HepaRG and Hep‐Orgs in the coculture group. In contrast, *YAP1*, *PCNA*, and *POU5F1* were expressed at low levels in both the coculture groups (Figure S7a, b, Supporting Information). These results were consistent with the data obtained from qRT‐PCR and ELISA (Figure 2c,d,g–j), indicating that coculturing with intestinal cells promotes the hepatic differentiation of HPCs. According to KEGG enrichment analysis of biological processes, the pathways enriched in HepaRG cells were primarily related to the functional metabolism of hepatocytes, such as cholesterol biosynthesis, drug metabolism, and complement activation (Figure S7d—f, Supporting Information). Pathways enriched in Hep‐Orgs included glutamic acid metabolism, vitamin absorption, glucose metabolism, bile acid secretion, lipid metabolism, glycogen synthesis, and drug metabolism (Figure S7g—I, Supporting Information). Moreover, several overlapping DEGs participated in the EGFR‐MEK‐ERK pathway (*NTF4*, *EGFR*, *CREB1*, *RPS6KA2*) and the downstream classical MAPK pathway (*MYC*, *FOSL1*, *FOS*, *CCND1*) (Figure S7f, I, Supporting Information). Additionally, the enriched pathways encompassed the Hippo, WNT, and AMPK signalling pathways (Figure S7j, Supporting Information).

### 2.3 EXOs of Caco‐2/Int‐Orgs Promoted Hepatogenic Differentiation of HepaRG/Hep‐Orgs

**Figure 3:**
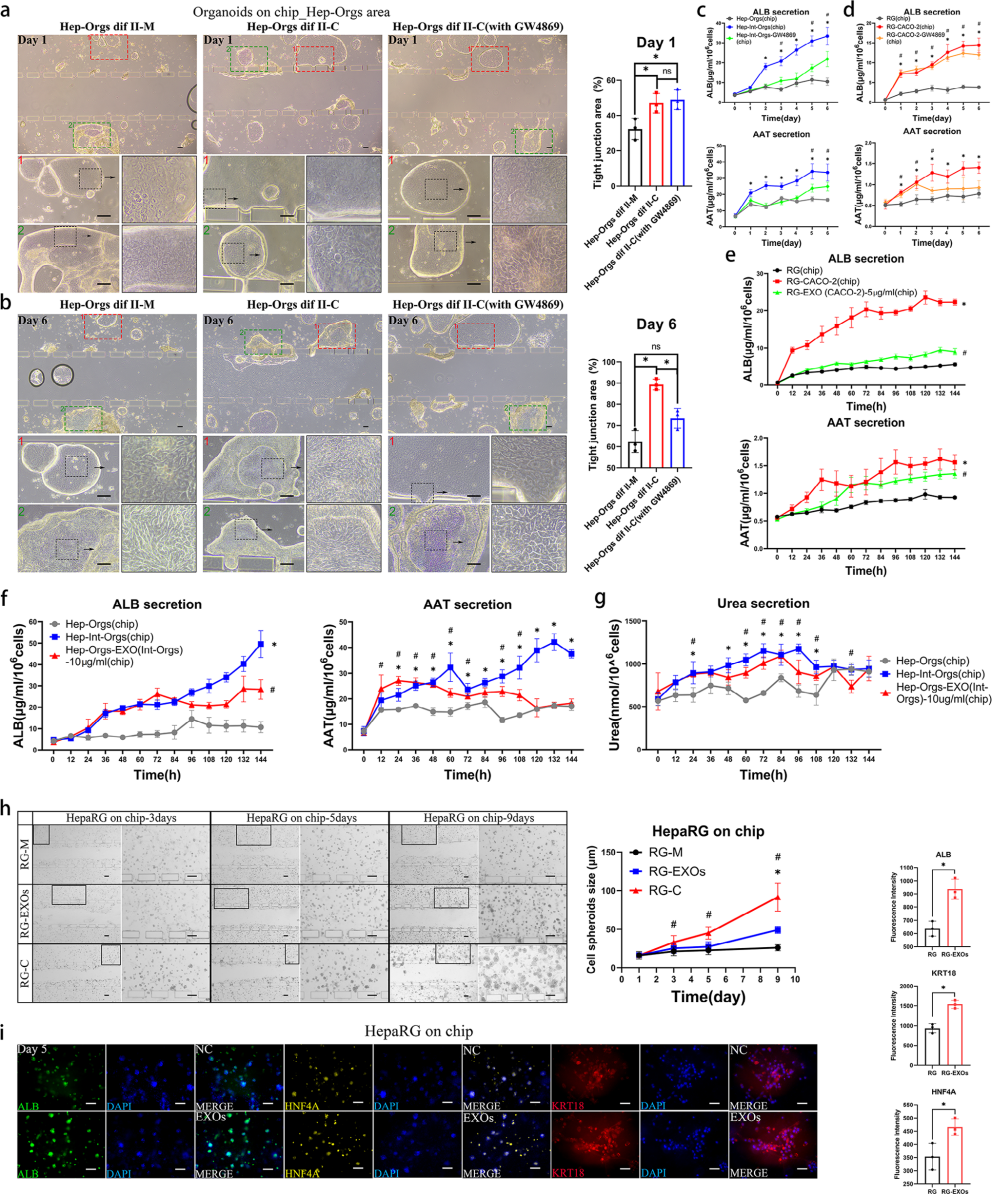
The effects of intestinal EXOs inhibition on the hepatic differentiation of HPCs (HepaRG/Hep‐Orgs). a) Bright‐field images of Hep‐Orgs on the chip at the differentiation stage II (Day 1). Upper panels and lower panels: Hep‐Orgs on chip under monoculture (left), coculture (middle), and coculture treated with GW4869 (right) at differentiation stage II (Day 1), and high‐magnification images corresponding to the squares. Scale bar = 100 µm. The column chart on the right shows the proportion of the area of tight junction of Hep‐Orgs in panel (a) (*n* = 3, mean ± SD, One‐way ANOVA). ^*^ *p* < 0.05. b) Bright‐field images of Hep‐Orgs on the chip at the differentiation stage II (Day 6). Upper panels and lower panels: Hep‐Orgs on chip under monoculture (left), coculture (middle), and coculture treated with GW4869 (right) at differentiation stage II (Day 6) and high‐magnification images corresponding to the squares. Scale bar = 100 µm. The column chart on the right shows the proportion of the area of tight junction of Hep‐Orgs in panel (b) (*n* = 3, mean ± SD, One‐way ANOVA). ^*^ *p* < 0.05. c) The levels of secreted proteins (ALB, AAT) of Hep‐Orgs on chip were detected by ELISA in monoculture (control), coculture, and coculture treated with GW4869 during the 6 days of the differentiation stage II (*n* = 3, mean ± SD, One‐way ANOVA). ^*^ *p* < 0.05 for Hep‐Int‐Orgs(chip) versus Hep‐Orgs(chip), #*p* < 0.05 for Hep‐Int‐Orgs‐GW4869(chip) versus Hep‐Orgs(chip). d) Levels of secreted proteins (ALB, AAT) in HepaRG cells on the chip detected by ELISA in monoculture (control), coculture, and coculture treated with GW4869 during the 6 days of the differentiation stage II (*n* = 3, mean ± SD, One‐way ANOVA). ^*^ *p* < 0.05 for RG‐Caco‐2(chip) versus RG(chip), #*p* < 0.05 in RG‐Caco‐2‐GW4869(chip) versus RG(chip). e) The levels of secreted proteins (ALB, AAT) in monocultured, cocultured, and EXO‐treated HepaRG cells on the chip were detected by ELISA during 6 days of differentiation (stage II) (*n* = 3, mean ± SD, Two‐way ANOVA). ^*^ *p* < 0.05 in RG‐Caco‐2(chip) versus RG(chip), # *p* < 0.05 for RG‐EXO (Caco‐2)‐5 µg mL^−1^ (chip) versus RG(chip). f) The levels of secreted proteins (ALB, AAT) of Hep‐Orgs on the chip were detected by ELISA in monoculture, coculture, and EXO‐treated groups during the 6 day differentiation stage II (*n* = 3, mean ± SD, One‐way ANOVA). ^*^ *p* < 0.05 in Hep‐Int‐Orgs(chip) versus Hep‐Orgs(chip), #*p* < 0.05 for Hep‐Orgs‐EXO(Int‐Orgs)‐10 µg mL^−1^ (chip) versus Hep‐Orgs(chip). g) Urea synthesis of Hep‐Orgs in the different groups was measured by colorimetry within 6 days of differentiation II (*n* = 3, mean ± SD, One‐way ANOVA). ^*^ *p* < 0.05 in Hep‐Int‐Orgs(chip) versus Hep‐Orgs(chip), #*p* < 0.05 in Hep‐Orgs‐EXO(Int‐Orgs)‐10 µg mL^−1^ (chip) versus Hep‐Orgs(chip). h) HepaRG cells in monoculture (RG‐M), EXO‐treated (RG‐EXOs), and coculture (RG‐C) groups were cultured on chips for three, five, and nine days. The corresponding high‐magnification images of the squares are shown on the right side. Scale bar = 100 µm. The line graph on the right presents the size variations of the HepaRG cell spheroids in panel (h) (*n* = 3, mean ± SD, One‐way ANOVA). ^*^ *p* <0.05, RG‐EXOs versus RG‐M; # *p* <0.05, RG‐C versus RG‐M. i) Expression of hepatic cell markers, including ALB (green), KRT18 (red), and HNF4A (yellow), in monocultured and EXO‐treated HepaRG cells on the chip, detected by immunostaining on day 5. Scale bar = 100 µm. The charts show the fluorescence intensity of the proteins in each group (*n* = 3, mean ± SD, Student's t‐tests). ^*^ *p* < 0.05.

**Figure 4:**
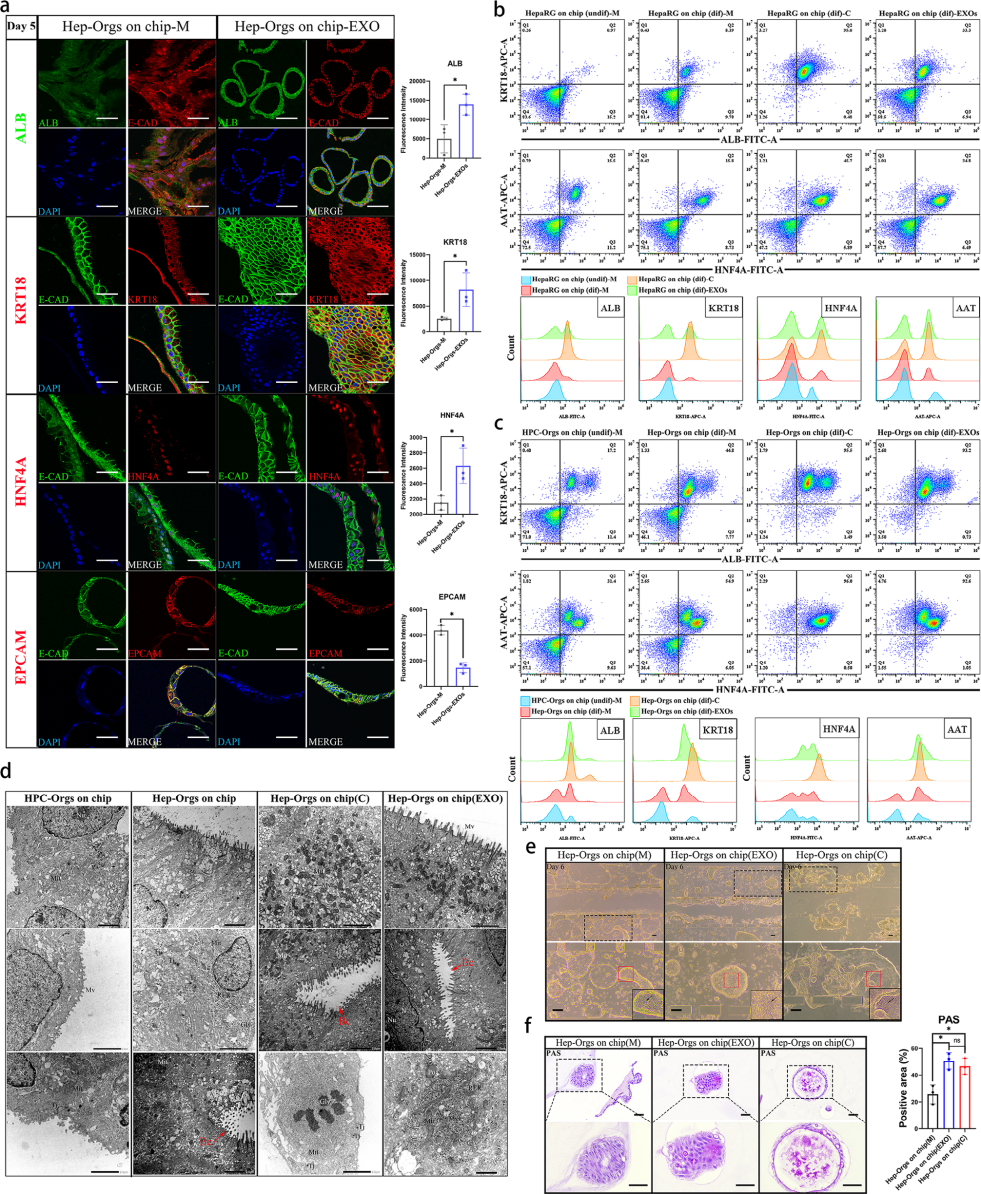
Changes in hepatic function and morphology of Hep‐Orgs cultured in the coculture system and EXO‐treated system. a) The expression of ALB (green), KRT18 (red), and HNF4A (red) in hepatocytes, as well as EPCAM (red), in the monocultured and EXO‐treated Hep‐Orgs on the chip on Day 5 of the differentiation stage II detected by immunostaining. *E‐CAD*, red or green; DAPI, blue. Scale bar = 100 µm. The bar charts show the fluorescence intensity of detected proteins (*n* = 3, mean ± SD, Student's t‐tests). ^*^ *p* < 0.05. b) Scatter plots showing the expression of ALB (FITC), KRT18 (APC), HNF4A (FITC), and AAT (APC) in HepaRG on‐chip (undif)‐M, HepaRG on‐chip (dif)‐M, HepaRG on‐chip (dif)‐C, and HepaRG on‐chip (dif)‐EXOs groups after 5 days, as detected by flow cytometry. M, monoculture; C, coculture with Caco‐2 cells; EXOs, treatment with EXOs from Caco‐2 cells. Histograms show the differences in the expression of these indices among the groups (*n* = 3). c) The scatter plots showed the expression of ALB (FITC), KRT18 (APC), HNF4A (FITC), and AAT (APC) in HPC‐Orgs on the chip (undif)‐M, Hep‐Orgs on the chip (dif)‐M, Hep‐Orgs on the chip (dif)‐C, and Hep‐Orgs on the chip (dif)‐EXOs groups after 6 days, detected by Flow cytometry. M = monoculture; C = cocultured with Int‐Orgs; EXOs = treated with EXOs of Int‐Orgs. The histograms show the difference in the expression of these indexes among the groups (*n* = 3). d) The first column panel: TEM images of HPC‐Orgs on the chip. N = Nucleus, Nu = Nucleoli, Mit = Mitochondria, Tj = Tight junction, Mv = Microvilli; The second column panel: TEM image of Hep‐Orgs on the chip. Lys = lysosome, Bc = bile canaliculi, RER = rough endoplasmic reticulum, Gly = glycogen storage. The red arrow points to the bile canaliculi. The third column panel: TEM images of Hep‐Orgs cocultured with Int‐Orgs on the chip at the differentiation stage II (Day 5). Mvb = Multivesicle bodies. The red arrow points to the bile canaliculi. The fourth column panel: TEM image of EXO‐treated Hep‐Orgs on the chip. Po = Peroxisome, Mvs = Microvesicles. The red arrow points to the bile canaliculi. Scale bar = 2 µm. e) Upper panels: bright‐field images of Hep‐Orgs on the chip under monoculture (left), coculture (right), and EXO‐treated (middle) conditions at the differentiation stage II (Day 6). Lower panels: The corresponding high‐magnification images of the squares in the upper panels and the cells framed by a red box in the panels are magnified in the lower right corner (200×). Scale bar = 100 µm. f) PAS staining of Hep‐Orgs on the chip under monoculture (left), coculture (right), and EXO‐treated (middle) conditions at the differentiation II stage (Day 6). Scale bar = 100 µm. The column chart on the right shows the proportion of the PAS‐stained area in panel (h) (*n* = 3, mean ± SD, One‐way ANOVA). ^*^ *p* < 0.05, ns: not significant.

To determine whether the promotion of hepatic differentiation was mediated by EXOs from intestinal epithelial cells, we added EXOs and the exosome inhibitor, GW4869,^[^ ^35^, ^36^, ^37^ ^]^ to the coculture chip system. The optimal concentration of GW4869 was determined based on cell activity and EXOs inhibition efficiency (Figure S8a, b, Supporting Information). After six days of differentiation on the chip, Hep‐Orgs in the coculture group exhibited the most pronounced features of differentiation, characterized by polygonal cell morphology, clear intercellular spaces, and tightly interlaced intercellular connections (**Figure** **3**a,b). On both day 1 and day 6, the proportion of the tight junction area in the co‐culture group was 1.5 times that of the monoculture group (Figure 3a,b). However, in the coculture group treated with GW4869, Hep‐Orgs/HepaRG cells displayed a noticeable but delayed differentiation, as indicated by reduced levels of ALB and AAT (Figure 3c,d) and a 0.3‐fold decrease proportion of the intercellular tight junction area (Figure 3b). After this, nanoparticle tracking analysis (NTA) and transmission electron microscopy (TEM) were performed in order to determine the morphology and particle size of EXOs extracted from Caco‐2 and Int‐Orgs (Figure S8c, d, e, Supporting Information). The positive markers (TSG101*/*HSP70*/*CD63) and negative marker (calnexin) of the EXOs from Caco‐2 cells were confirmed by western blotting (Figure S8f, Supporting Information). NANOVIEW was used with specific antibodies (CD63*/*CD81*/*CD9) to identify the EXOs of Int‐Orgs (Figure S8g, Supporting Information). The uptake of EXOs (labelled with PHK26) was observed in HepaRG cells after 48 h of perfusion on the chip (Figure S8h, Supporting Information). Similarly, labelled EXOs were detected in Hep‐Orgs–24‐48 h after perfusion on the chip (Figure S8i, j, Supporting Information). After being treated with EXOs derived from Caco‐2, both HepaRG and Hep‐Orgsproduced higher levels of ALB, AAT, and urea compared with the untreated groups (Figure 3e–g). During a 9‐day continuous culture on the chip, HepaRG cells co‐cultured with Caco‐2 cells aggregated into spheroids, a phenomenon that was also observed in the EXO‐treated group (Figure 3h). Immunostaining analysis demonstrated a significant increase in the protein levels of ALB, KRT18, and HNF4A in HepaRG cells and Hep‐Orgs treated with EXOs on the chip for 5 days compared with the untreated group (Figure 3i and **Figure** **4**a). Flow cytometry analysis indicated that the expression levels of ALB, AAT, KRT18, and HNF4A in Hep‐Orgs and HepaRG cells were significantly enhanced under co‐culture conditions or upon exosome treatment compared with those in the monoculture group (Figure 4b,c). qRT‐PCR data also showed enhanced expression of hepatocyte markers (*ALB*, *KRT18*, *CYP3A4*, and *AAT*) and reduced expression of progenitor cell markers (*AFP*, *EPCAM*, and *SOX9*) in both the EXO‐treated and co‐cultured groups (Figure S9, Supporting Information). In contrast to monocultured Hep‐Orgs, EXO‐treated and co‐cultured Hep‐Orgs exhibited ultrastructural features closely resembling primary hepatocytes, such as polygonal cell morphology, tightly interlaced intercellular connections, mitochondrial enrichment, widespread distribution of rough endoplasmic reticulum, prominent bile canaliculi structures, increased presence of microvesicles, and aggregation of multivesicular bodies around the endoplasmic reticulum, as shown by TEM (Figure 4d). Figure 4e shows similar changes in the polygonal cell morphology and tightly interlaced intercellular connections among the groups. PAS staining revealed a 2 fold increase in glycogen accumulation in EXO‐treated Hep‐Orgs, as shown in Figure 4f. The above data demonstrated that EXOs of Caco‐2/Int‐Orgs promoted hepatogenic differentiation of HepaRG/Hep‐Orgs.

### 2.4 EXOs from Human Int‐Orgs Alleviated the Development of CCL4‐Induced Liver Fibrosis in Mice

**Figure 5:**
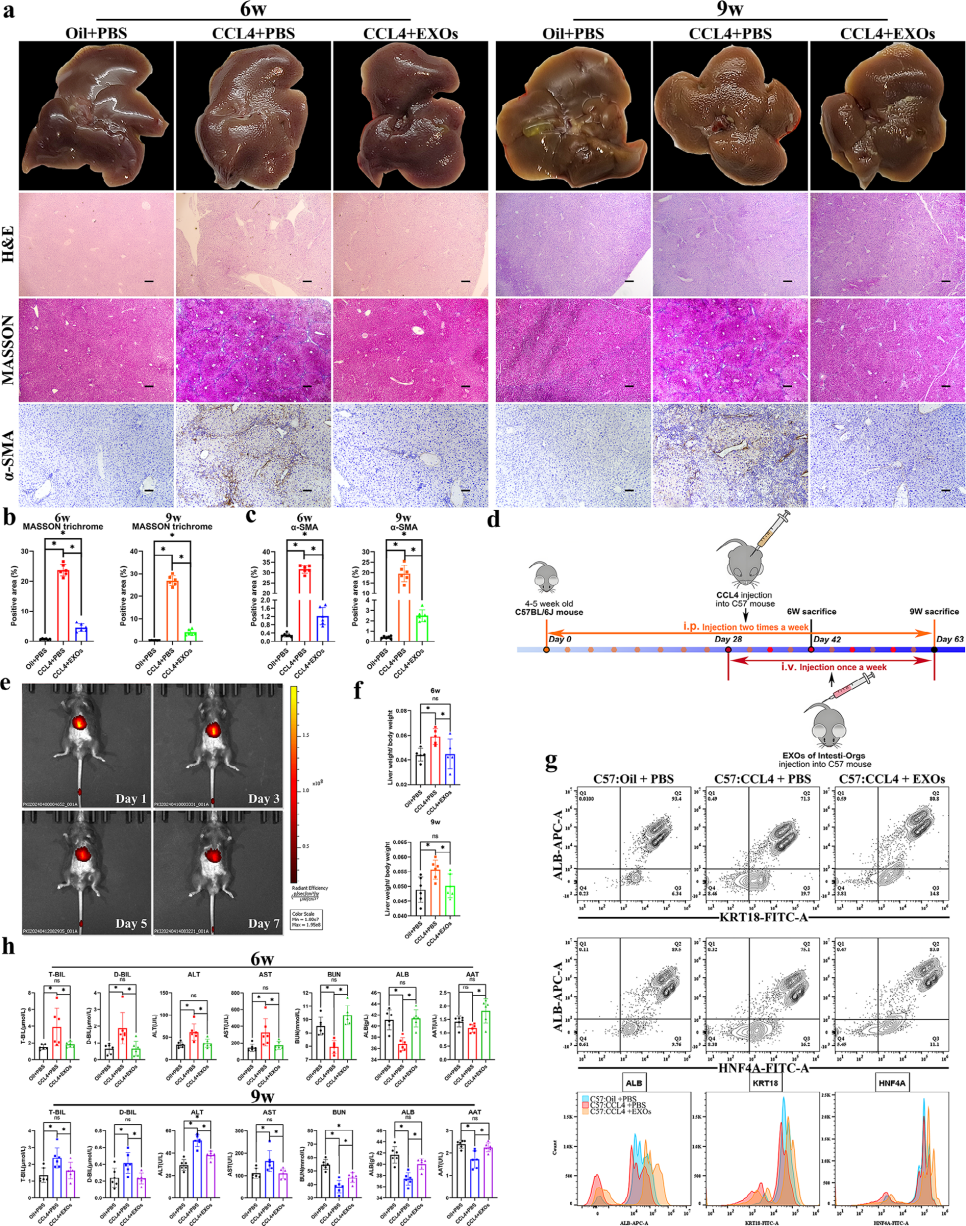
Treatment of EXOs from Int‐Orgs improved the hepatic fibrosis in mice. a) The macrograph, HE staining, MASSON trichrome staining, and α‐SMA staining images of CCL4‐induced hepatic fibrosis in mice at 6 weeks and 9 weeks in the control groups (Oil+PBS), modelling groups (CCL4+PBS), and treatment groups (CCL4+EXOs). Scale bar = 100 µm. b) The histogram shows the positive area (%) of Masson trichrome staining of the different groups (6w‐left, 9w‐right) (*n* = 6, mean ± SD, One‐way ANOVA). ^*^ *p* < 0.05. c) The histogram shows the positive area (%) of α‐SMA staining of the different groups (6w‐left, 9w‐right) (*n* = 6, mean ± SD, One‐way ANOVA). ^*^ *p* < 0.05. d) Schematic diagram of the procedures of animal experiments. e) Distribution of EXOs in vivo (day 1, day 3, day 5, and day 7 after EXOs injection). f) The ratios of liver weight to body weight of 6w (upper) and 9w (lower) CCL4‐induced mice (*n* = 6, mean ± SD, One‐way ANOVA). ^*^ *p* < 0.05. g) The scatter plots showed the expression of ALB (APC), KRT18 (FITC), and HNF4A (FITC) in C57(9w): Oil + PBS, C57(9w): CCL4 + PBS, and C57(9w): CCL4 + EXOs groups detected by Flow cytometry. The histograms show the difference in the expression of these indexes among the groups (*n* = 6). h) Serum levels of T‐BIL, D‐BIL, ALT, AST, BUN, ALB, and AAT in control groups (Oil+PBS), modelling groups (CCL4+PBS), and treatment groups (CCL4+EXOs) (*n* = 6, mean ± SD, One‐way ANOVA). ^*^ *p* < 0.05.

**Figure 6:**
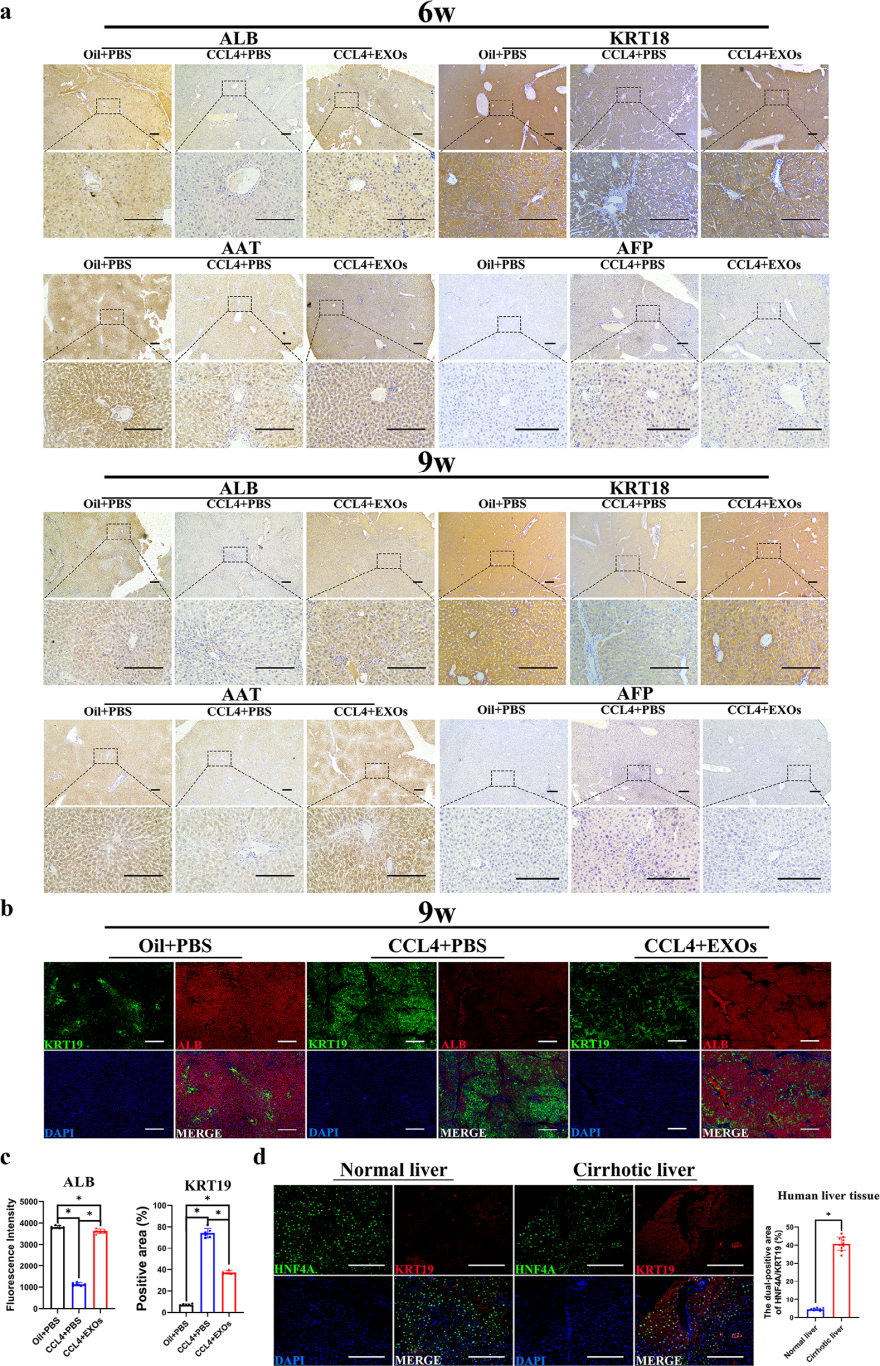
Treatment of EXOs from Int‐Orgs increased the differentiation and functional expression of hepatocytes in mice with hepatic fibrosis. a) IHC staining images show the expression of ALB, KRT18, AAT, and AFP of mice liver at 6 weeks (upper) and 9 weeks (down) in the control groups (Oil+PBS), modelling groups (CCL4+PBS), and treatment groups (CCL4+EXOs). Scale bar = 100 µm. b) Immunofluorescence staining images show the expression of ALB and KRT19 of mice liver at 9 weeks in the control groups (Oil+PBS), modelling groups (CCL4+PBS), and treatment groups (CCL4+EXOs). ALB, red; KRT19, green; DAPI, blue. Scale bar = 100 µm. c) The column chart (left) shows the fluorescence intensity of ALB in panel (b). The column chart (right) shows the positive expression area of KRT19 in panel (b) (*n* = 6, mean ± SD, One‐way ANOVA). ^*^ *p* < 0.05. d) Immunofluorescence staining images show the expression of HNF4A and KRT19 in human liver in normal liver groups and cirrhotic liver groups. Scale bar = 100 µm. The column chart on the right presents the proportion of the HNF4A/KRT19 dual‐positive area in panel (d) (*n* = 10, mean ± SD, Student's t‐tests). ^*^ *p* < 0.05.

Next, we investigated the therapeutic effects of human Int‐Orgs on CCL4‐induced liver fibrosis. Following a 6‐week modelling with CCL4, the livers of the mice exhibited evident tissue structural abnormalities, including fibrous tissue hyperplasia, hepatocyte degeneration, necrosis, and pseudolobular formation (**Figure** **5**a–c). The EXOs of 1‐week differentiated Int‐Orgs were injected into mice with liver fibrosis through the tail vein (Figure 5d). In vivo imaging showed that the DiR‐labelled EXOs were mostly enriched in the liver, and the signals remained high level for 7 days (Figure 5e). The mice were intravenously injected (i.v.) with EXOs (40ug/time, dissolved in PBS) of Int‐Orgs or PBS solution once a week starting at week 4. The Hematoxylin–eosin (HE) staining, MASSON trichrome staining, and α‐SMA staining results displayed that treatment with EXOs led to a significant reduction of the liver fibrotic area in mice with 6‐week and 9‐week CCL4‐induced liver fibrosis (Figure 5a–c). After 2 or 5 weeks of treatment with EXOs, mice with CCL4‐induced liver fibrosis (CCL4+ EXOs) exhibited liver weight/body weight ratios similar to those in the control groups (Oil+ PBS) (Figure 5f). Additionally, the levels of serum TBIL, DBIL, ALT, and AST decreased, whereas the secretion of proteins (ALB and AAT) and the level of BUN significantly increased in the EXO‐treated mice, which was consistent with the flow cytometry results (Figure 5g,h). Immunohistochemical (IHC) analysis of tissue sections showed decreased expression of ALB, AAT, and KRT18 within the liver fibrosis region of CCL4‐induced mice (**Figure** **6**a). Conversely, the expression levels of these markers in the fibrotic regions of mice treated with EXOs were comparable to those in normal liver tissues (Figure 6a). Additionally, AFP expression decreased in the EXO‐treated group (Figure 6a). Dual‐fluorescence staining showed that, compared with the liver fibrosis group (treated with CCL4+PBS), the expression of ALB (a hepatocyte‐specific marker) in hepatocytes in the fibrotic area of the treatment group (treated with CCL4+EXOs) was increased, whereas the expression of KRT19 (a marker for bile ducts) was diminished (Figure 6b,c). After 2 or 5 weeks of EXOs treatment, improvements in the pathologic manifestations of extrahepatic organs (including the spleen, lung, and intestine) of the mice were also observed, as shown by the results of HE staining (Figure S10, Supporting Information). These data suggested that treatment with EXOs alleviated the progression of liver fibrosis, likely by promoting hepatocyte differentiation and suppressing cholangiocyte differentiation in fibrotic liver tissues. To determine the relative proportion of hepatic progenitor cells in human fibrotic liver tissues, normal human and cirrhotic liver tissues were obtained and subjected to HNF4A/KRT19 dual‐fluorescence staining. The results showed that the proportion of hepatic progenitor cells (HNF4A+/KRT19+) in human cirrhotic liver tissues was ≈7 fold higher than that in normal liver tissues (Figure 6d).

### 2.5 The miR‐371‐373 Cluster Within the EXOs from the Intestinal Epithelial Cells Improved the Hepatic Differentiation of HPCs

Next, we explored the molecular mechanisms involved in the regulation of HPC differentiation by EXOs derived from intestinal epithelial cells. Eleven miRNAs were selected (miR‐192‐5p, miR‐371a‐5p, miR‐191‐5p, miR‐182‐5p, miR‐183‐5p, miR‐215‐5p, miR‐372‐3p, miR‐373‐3p, miR‐200b‐3p, miR‐194‐5p, miR‐141‐3p) from the top 50 miRNAs ranked by total read count in the sequencing data of EXOs from Caco‐2 (Figure S11a, Supporting Information). These miRNAs are known to be highly expressed in intestinal tissues but have relatively low expression in liver tissues (EVmiRNA database (http://bioinfo.life.hust.edu.cn/EVmiRNA)). qRT‒PCR results confirmed that in coculture with EXOs from Caco‐2 cells, the levels of these miRNAs increased in HepaRG cells, thus indicating that they can be taken up by HepaRG cells (Figure S11b, Supporting Information). Then, qRT‒PCR was performed to investigate the effects of the 11 miRNAs mimics (100 µM) on HepaRG differentiation‐ and stemness‐related genes in mono‐ and coculture Transwell models. The results showed that the addition of miR‐371a‐5p, miR‐372‐3p, or miR‐373‐3p mimics increased the expression of a series of genes in mono‐ and co‐cultured HepaRG cells, including hepatocyte marker genes (*ALB*, *AAT*, *ARG*, *TTR*, *HNF4A*, *E‐CAD*, *KRT18*), phase I drug metabolism enzyme‐encoding genes (*CYP3A4*, *CYP1A2*, *CYP2D6*), and phase II drug metabolism enzyme‐encoding gene *UGT1A1* (Figure S11c, d, Supporting Information). Furthermore, ELISA data confirmed that among the tested miRNAs, miR‐373‐3p, miR‐371a‐5p, and miR‐372‐3p mimics ranked among the top 3 in significantly increasing ALB secretion by HepaRG cells under monoculture conditions (Figure S11e, Supporting Information). Similar effects of miR‐371a‐5p, miR‐372‐3p, and miR‐373‐3p on HepaRG cells were observed under coculture conditions (Figure S11f, Supporting Information). Next, the expression levels of 11 miRNAs were compared in Caco‐2 cells, Int‐Orgs (undif and dif), and EXOs (Caco‐2 cells and differentiated Int‐Orgs). Surprisingly, differentiated Int‐Orgs and their EXOs showed a much higher expression of miR‐371a‐5p, miR‐372‐3p, and miR‐373‐3p than Caco‐2 cells (Figure S11g–i, Supporting Information). Increased expression of these three miRNAs was also detected in Hep‐Orgs cocultured with Int‐Orgs (Dif) (Figure S11j, Supporting Information).

**Figure 7:**
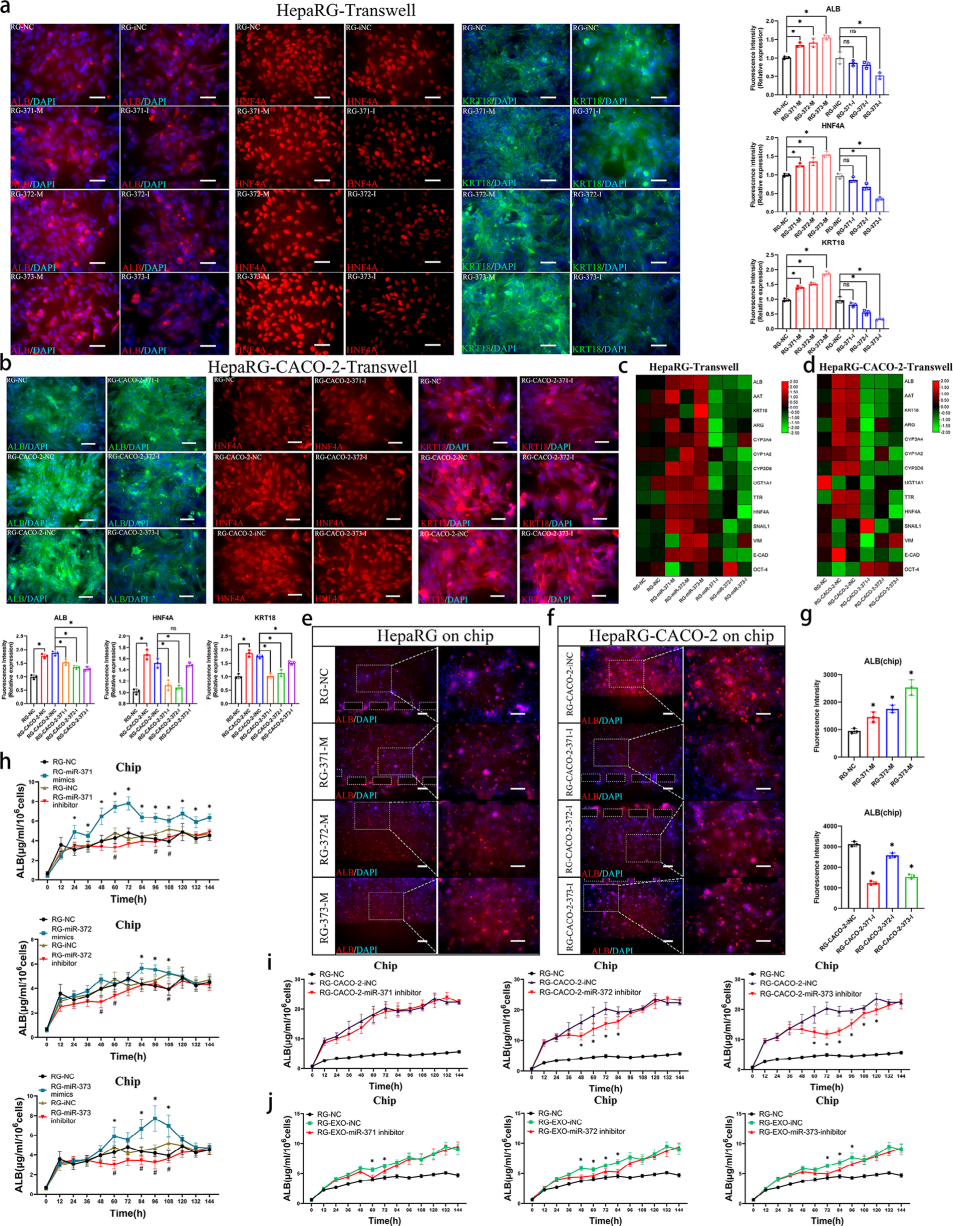
Regulatory effects of the miR‐371‐373 cluster on the differentiation of HPCs a) The expression of hepatic cell markers in HepaRG cells (cultured in Transwell) transfected with miR‐371‐373 cluster mimics and inhibitors detected by immunostaining on Day 3 after transfection. RG‐NC: HepaRG cells transfected with NC; RG‐371‐M: HepaRG cells transfected with miR‐371a‐5p mimics; RG‐371‐I: HepaRG cells transfected with miR‐371a‐5p inhibitor; RG‐372‐M: HepaRG cells transfected with miR‐372‐3p mimics; RG‐372‐I: HepaRG cells transfected with miR‐372‐3p inhibitor; RG‐373‐M: HepaRG cells transfected with miR‐373‐3p mimics; RG‐373‐I: HepaRG cells transfected with miR‐373‐3p inhibitor; *ALB* (red), *HNF4A* (red), *KRT18* (green), and DAPI (blue). Scale bar = 100 µm. The charts show the fluorescence intensity of *ALB* (upper), *HNF4A* (middle), and *KRT18* (bottom) (*n* = 3, mean ± SD, One‐way ANOVA). ^*^p<0.05. b) The expression of hepatic cell markers in HepaRG cells cocultured with Caco‐2 cells (transfected with miR‐371‐373 cluster inhibitors) was detected by immunostaining on Day 3 after coculture. RG‐NC: HepaRG cells transfected with NC; RG‐Caco‐2‐NC: HepaRG cells cocultured with Caco‐2 cells (transfected with NC); RG‐Caco‐2‐iNC: HepaRG cells cocultured with Caco‐2 cells (transfected with iNC); RG‐Caco‐2‐371‐I: HepaRG cells cocultured with Caco‐2 cells (transfected with miR‐371a‐5p inhibitor); RG‐Caco‐2‐372‐I: HepaRG cells cocultured with Caco‐2 cells (transfected with miR‐372‐3p inhibitor); RG‐Caco‐2‐373‐I: HepaRG cells cocultured with Caco‐2 cells (transfected with miR‐373‐3p inhibitor); *ALB* (green), *HNF4A* (red), *KRT18* (red), and DAPI (blue). Scale bar = 100 µm. The charts show the fluorescence intensity analysis of *ALB* (left), *HNF4A* (middle), and *KRT18* (right) (*n* = 3, mean ± SD, One‐way ANOVA). ^*^ *p* < 0.05. c) The expression levels of genes related to hepatic differentiation and stemness in HepaRG cells (cultured in Transwell), transfected with miR‐371‐373 cluster mimics and inhibitors measured by qRT‒PCR. The heatmap shows the log2(fold change) value of the genes (*n* = 3). d) The expression levels of genes related to hepatic differentiation and stemness in HepaRG cells cocultured with Caco‐2 cells (transfected with the miR‐371‐373 cluster inhibitors) were measured by qRT‒PCR. The heatmap shows the log2(fold change) value of the genes (*n* = 3). e) The effects of miR‐371‐373 cluster mimics on *ALB* expression in HepaRG cells on the chip were detected by immunostaining on Day 3 after transfection. *ALB* (red) and DAPI (blue). Scale bar = 100 µm. f) *ALB* expression in HepaRG cells cocultured with Caco‐2 cells (transfected with miR‐371‐373 cluster inhibitors) on the chip was detected by immunostaining on Day 3 after coculture. *ALB* (red) and DAPI (blue). Scale bar = 100 µm. g) The upper and lower panels display the fluorescence intensity analysis of *ALB* in panel (e) and panel (f), respectively (*n* = 3, mean ± SD, One‐way ANOVA). ^*^ *p* < 0.05, compared with RG‐NC (negative control). h) The secreted *ALB* levels of HepaRG cells transfected with the mimics or inhibitor of miR‐371a‐5p (upper panel)/372‐3p (middle panel)/373‐3p (lower panel) on the chip were measured by ELISA during 6 days after transfection (*n* = 3, mean ± SD, One‐way ANOVA). ^*^ *p* < 0.05 for RG‐miR‐371 mimics versus RG‐NC, #*p* < 0.05 for RG‐miR‐371 inhibitor versus RG‐iNC (upper panel); ^*^ *p* < 0.05 for RG‐miR‐372 mimics versus RG‐NC, #*p* < 0.05 for RG‐miR‐372 inhibitor versus RG‐iNC (middle panel); ^*^ *p* < 0.05 for RG‐miR‐373 mimics versus RG‐NC, #*p* < 0.05 for RG‐miR‐373 inhibitor versus RG‐iNC (lower panel). i) Secreted *ALB* levels in HepaRG cells cocultured with Caco‐2 cells (transfected with miR‐371a‐5p (left panel)/372‐3p (middle panel)/373‐3p (right panel) inhibitor) on the chip measured by ELISA within 6 days after transfection (*n* = 3, mean ± SD, One‐way ANOVA). ^*^ *p* < 0.05 for RG‐Caco‐2‐miR‐371 inhibitor versus RG‐Caco‐2‐iNC (left panel); ^*^p<0.05 for RG‐Caco‐2‐miR‐372 inhibitor versus RG‐Caco‐2‐iNC (middle panel); ^*^ *p* < 0.05 for RG‐Caco‐2‐miR‐373 inhibitor versus RG‐Caco‐2‐iNC (right panel). j) Secreted *ALB* levels in HepaRG cells treated with EXOs from Caco‐2 cells (transfected with miR‐371a‐5p (left panel)/372‐3p (middle panel)/373‐3p (right panel) inhibitor) on the chip measured by ELISA during 6 days after transfection (*n* = 3, mean ± SD, One‐way ANOVA). ^*^ *p* < 0.05 for RG‐EXO‐miR‐371 inhibitor versus RG‐EXO‐iNC; ^*^p<0.05 for RG‐EXO‐miR‐372 inhibitor versus RG‐EXO‐iNC; ^*^p<0.05 for RG‐EXO‐miR‐373 inhibitor versus RG‐EXO‐iNC.

Based on these aforementioned findings, miR‐371a‐5p, miR‐372‐3p, and miR‐373‐3p were selected as potential candidates for further investigation. These miRNAs belong to the miR‐371‐373 cluster, which originates from the same chromatin region (Figure S12a, Supporting Information).^[^ ^38^, ^39^, ^40^, ^41^ ^]^ The transfection efficiencies of the mimics and inhibitors of the miR‐371‐373 cluster in different culture systems were validated (Figure S12b–f, Supporting Information). Immunofluorescence analysis and qRT‐PCR results from Transwell models showed that treatment with miR‐371‐373 mimics upregulated the expression of ALB, HNF4A, and KRT18, whereas inhibitors downregulated their expression in both mono‐ and co‐cultured HepaRG cells (**Figure** **7**a–d). MiR‐372‐3p and miR‐373‐3p showed stronger effects on these proteins than miR‐371a‐5p (Figure 7a). Immunofluorescence analysis conducted on the chip revealed that each mimic of the miR‐371‐373 cluster significantly upregulated ALB expression in monocultured HepaRG cells, whereas each corresponding inhibitor resulted in a decrease in ALB expression in co‐cultured HepaRG cells compared to untreated co‐cultured cells. (Figure 7e–g). Similar effects were also confirmed when validating the effects by ELISA (Figure 7h,i), although ALB secretion exhibited a similar trend without statistical significance in the group treated with the miR‐371a‐5p inhibitor (Figure 7i). Next, we investigated whether miR‐371‐373 mediates the regulation of intestinal EXOs to promote liver differentiation. Our data revealed that the addition of EXOs derived from intestinal epithelial cells induced ALB production, whereas the presence of each inhibitor in the miR‐371‐373 cluster suppressed this effect (Figure 7j). These findings suggest that the EXOs from intestinal epithelial cells promote hepatic differentiation by mediating the miR‐371‐373 cluster.

### 2.6 RPS6KA2, as the Common Target of the miR‐371‐373 Cluster, was Involved in the Regulation of Hepatic Differentiation of HPC

**Figure 8:**
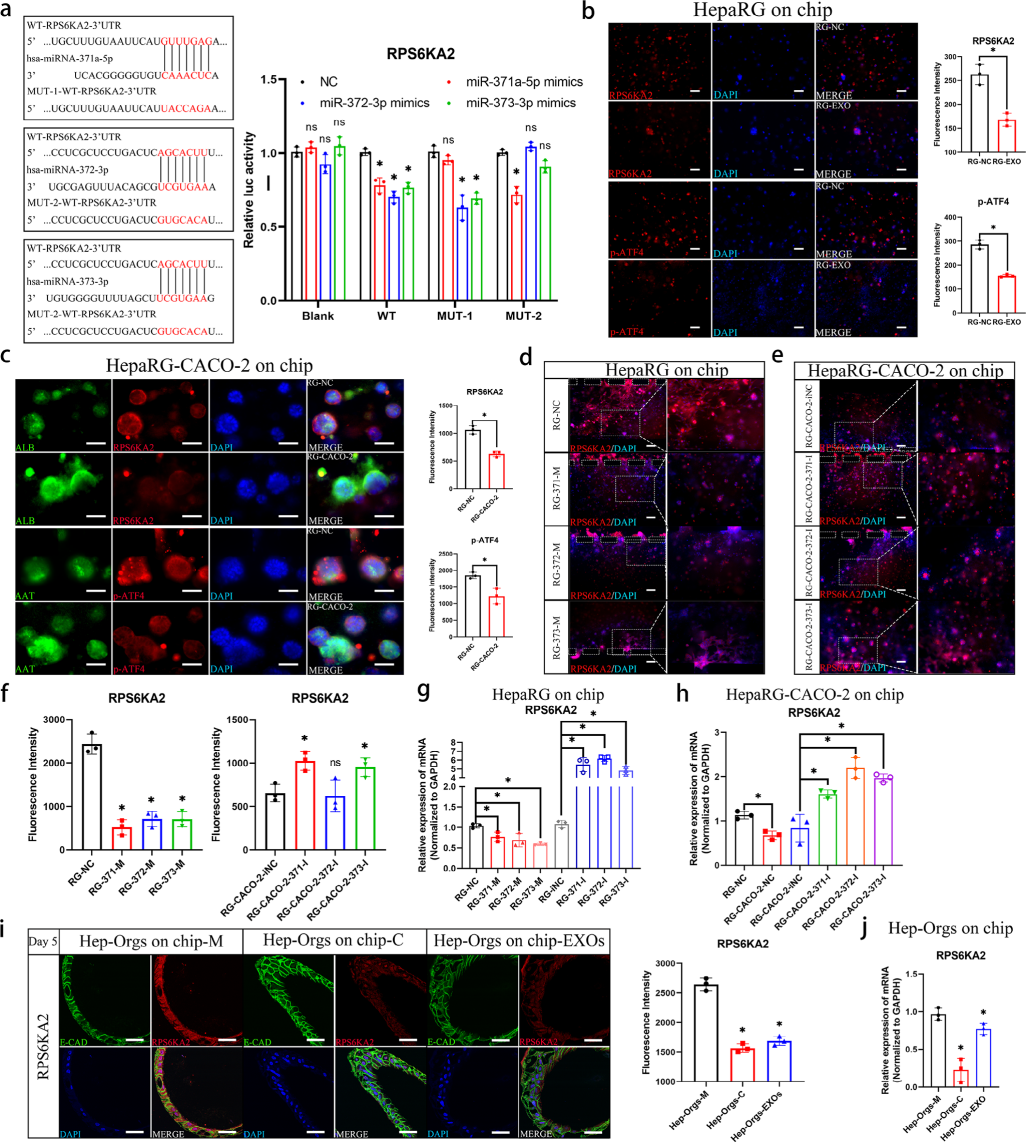
Validation of the miR‐371‐373 cluster target gene *RPS6KA2* a) Dual‐luciferase reporter assays were performed to test putative binding sites between *RPS6KA2* 3′ UTR and miR‐371a‐5p/372‐3p/373‐3p. The wide type (WT) sequence and mutant (MUT) sequence containing the binding site were inserted into the luciferase reporter vector. Luciferase activity indicated the molecular interactions between miR‐371a‐5p/372‐3p/373‐3p mimics and RPS6KA2 mRNA, as shown in the chart on the right (*n* = 3, mean ± SD, One‐way ANOVA). ^*^ *p* < 0.05, compared with NC. b) The expression of *RPS6KA2* (red) and the downstream protein *p‐ATF4* (red) and DAPI (blue) in HepaRG cells with and without EXO treatment (on the chip), visualized by immunostaining on Day 5. Scale bar = 100 µm. The charts display the fluorescence intensity of *RPS6KA2* and *p‐ATF4* (*n* = 3, mean ± SEM, Student's t‐tests). ^*^ *p* < 0.05. c) Immunofluorescence staining visualizing the expression of functional proteins (ALB, AAT (green)), the target protein RPS6KA2 (red), the downstream protein p‐ATF4 (red), and DAPI (blue) in both monocultured and cocultured HepaRG cells on the chip on Day 5. Scale bar = 100 µm. The charts show fluorescence intensity of the detected proteins (*n* = 3, mean ± SD, Student's t‐tests). ^*^p<0.05. d) The expression of *RPS6KA2* (red) in HepaRG cells transfected with miR‐371‐373 cluster mimics was visualized by immunostaining on the chip on Day 3 after transfection. Scale bar = 100 µm. e) The expression of *RPS6KA2* (red) in HepaRG cells cocultured with Caco‐2 cells (transfected with the miR‐371‐373 cluster inhibitors) was detected by immunostaining on the chip on Day 3 after coculture. Scale bar = 100 µm. f) The charts show fluorescence intensity of *RPS6KA2* in Figure 8d (left panel) and e (right panel) (*n* = 3, mean ± SD, One‐way ANOVA). ^*^ *p* < 0.05, compared with RG‐NC. g) Comparison of *RPS6KA2* expression levels in HepaRG cells transfected with miR‐371‐373 cluster mimics or inhibitors (on chips) measured by qRT‒PCR (*n* = 3, mean ± SD, One‐way ANOVA). ^*^ *p* < 0.05. h) Gene expression levels of *RPS6KA2* in HepaRG cells cocultured with Caco‐2 cells (transfected with the miR‐371‐373 cluster inhibitors) on chips measured by qRT‒PCR (*n* = 3, mean ± SD, One‐way ANOVA). ^*^ *p* < 0.05. i) Monocultured, cocultured, and EXO‐treated Hep‐Orgs were extracted from the chip, and immunostaining images of RPS6KA2 (in red) in the paraffin sections of the Hep‐Orgs were captured using a confocal microscope on Day 5. E‐CAD was labelled in green and DAPI in blue. Scale bar = 100 µm. The chart shows the fluorescence intensity analysis of *RPS6KA2* (*n* = 3, mean ± SD, One‐way ANOVA). ^*^ *p* < 0.05, compared with Hep‐Orgs‐M. j) Gene expression levels of *RPS6KA2* in monocultured, cocultured, and EXO‐treated Hep‐Orgs (on chips) measured by qRT‒PCR (*n* = 3, mean ± SD, One‐way ANOVA). ^*^ *p* < 0.05, compared with Hep‐Orgs‐M.

**Figure 9:**
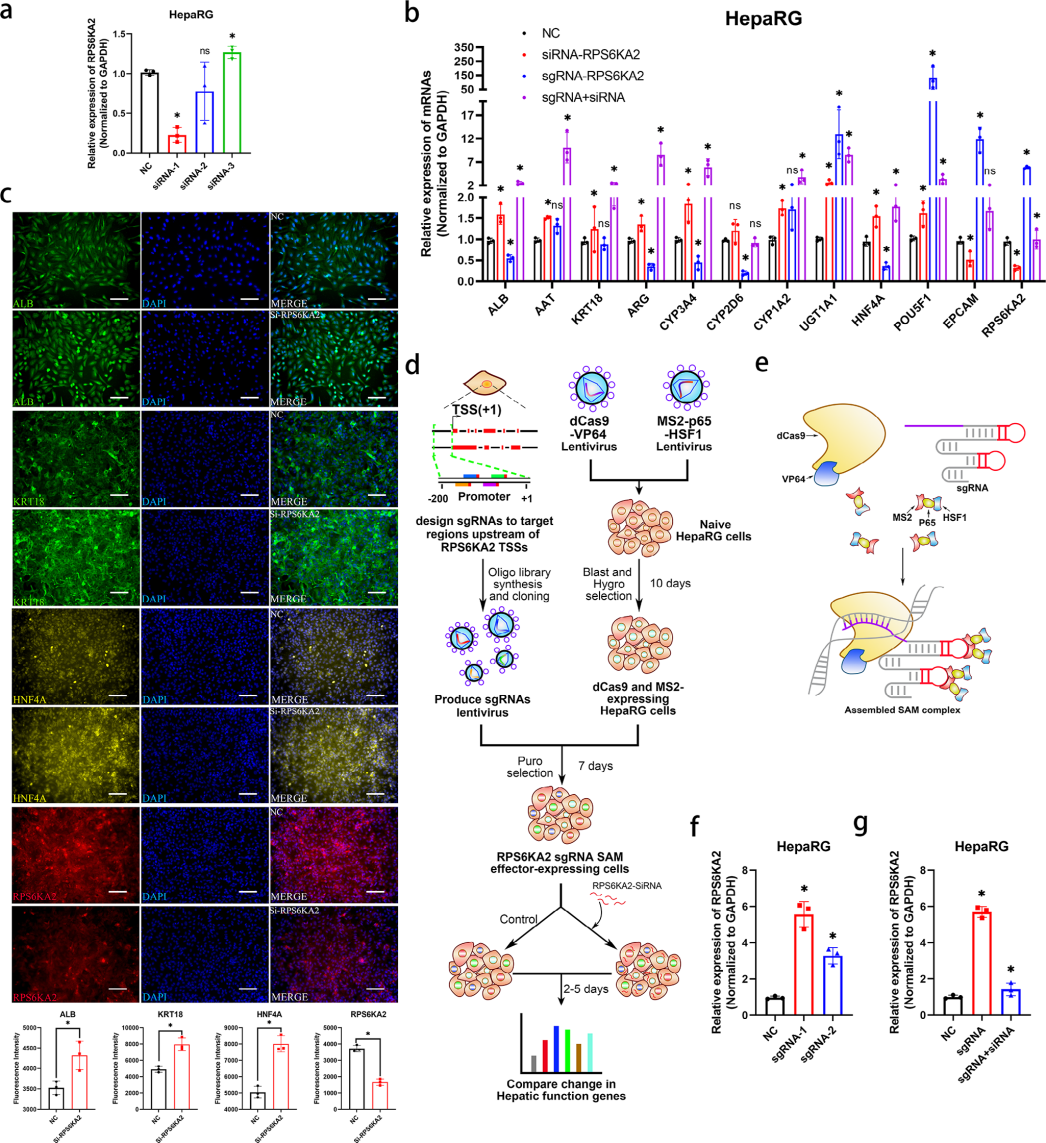
Verification of the function of RPS6KA2 on hepatic differentiation. a) The knockdown efficiency of *RPS6KA2* siRNAs in HepaRG cells was detected by qRT‐PCR (*n* = 3, mean ± SD, One‐way ANOVA). ^*^ *p* < 0.05, compared with NC. b) Expression of genes related to hepatic function and stemness in HepaRG cells (transfected with NC, *RPS6KA2* siRNA‐1, *RPS6KA2* sgRNA‐1, and *RPS6KA2* siRNA‐1 + sgRNA‐1) detected by qRT‐PCR at day 3 after transfection (*n* = 3, mean ± SD, One‐way ANOVA). ^*^ *p* < 0.05, compared with NC. c) Expression of ALB, KRT18 (green), HNF4A (yellow), and RPS6KA2 (red) in HepaRG cells (transfected with NC and RPS6KA2 siRNA‐1) detected by immunostaining at day 3 after transfection. DAPI (blue) shows the nuclei. Scale bar = 100 µm. The charts display the fluorescence intensity of proteins (*n* = 3, mean ± SD, One‐way ANOVA). ^*^ *p* < 0.05. d) Flow chart of transcription activation screening using sgRNA‐dCas9 SAM (synergistic activation mediator system). Blast, blasticidin; Hygro, hygromycin; Puro, puromycin. e) Schematic illustration of the three‐component SAM system, including RPS6KA2‐sgRNA, dCas9‐VP64, and MS2‐P65‐HSF1. f) The overexpression efficiency of *RPS6KA2* sgRNAs in HepaRG cells was detected by qRT‐PCR (*n* = 3, mean ± SD, One‐way ANOVA). ^*^ *p* < 0.05, compared with NC. g) The level of *RPS6KA2* expression in HepaRG cells was evaluated using qRT‐PCR following co‐transfection of siRNA‐1 and sgRNA‐1 or following transfection of sgRNA‐1(*n* = 3, mean ± SD, One‐way ANOVA). ^*^ *p* <0.05, compared with NC.

In order to further investigate the downstream target of the miR‐371‐373 cluster, the miRWalk miRNA (http://mirwalk.umm.uni‐heidelberg.de/) ensemble database was utilized. We found that RPS6KA2 was predicted as the common target of the miR‐371‐373 cluster. Notably, *RPS6KA2* was also found in previous sequencing results as a gene that was downregulated, with fold changes of 2.5 and 19.4 in the HepaRG and Hep‐Org coculture groups, respectively (Figure S7a, b, Supporting Information). This prediction was confirmed by the results of the dual‐luciferase reporter gene experiment, which showed that all three miRNA mimics downregulated the expression of *RPS6KA2*. MiR‐371‐373 has binding sites for *RPS6KA2*, and miR‐372‐3p and miR‐373‐3p share the same binding site (**Figure** **8**a). Immunofluorescence images of HepaRG cells showed that when either co‐cultured with Caco‐2 cells or treated with EXOs from Caco‐2 cells on the chip, the expression of RPS6KA2 and its downstream regulation of phosphorylated CREB2 (p‐ATF4) were significantly decreased compared to those in monocultured HepaRG cells (Figure 8b,c). Conversely, the downregulation of RPS6KA2 was increased in HepaRG cells co‐cultured on the chip after treatment with each inhibitor of the miR‐371‐373 cluster (Figure 8d–h). Additionally, we found the same decrease in the expression of RPS6KA2 in Hep‐Orgs when co‐cultured with Int‐Orgs or treated with EXOs on the chip (Figure 8i,j). To confirm the regulatory effect of RPS6KA2 on the hepatic differentiation of HPCs, the expression of RPS6KA2 was knocked down in HepaRG cells by transfecting *RPS6KA2*‐targeting siRNA (Si‐*RPS6KA2*) (**Figure** **9**a). Genes associated with hepatocyte function and differentiation, including *ALB*, *AAT*, *KRT18*, *ARG*, *CYP3A4*, *CYP2D6*, *UGT1A1*, and *HNF4A*, were upregulated in cells with RPS6KA2 gene knockdown (Figure 9b). Furthermore, immunofluorescence analysis demonstrated that the suppression of RPS6KA2 resulted in increased expression of ALB, HNF4A, and KRT18 (Figure 9c). To construct a cell line with stable overexpression of *RPS6KA2*, the synergistic activation mediator (SAM) transcriptional activation system was employed. The SAM system consisted of the dCas9 protein, guide RNA (gRNA), and a transcriptional activator module (MS2‐P65‐HSF1). gRNA guides the dCas9 protein to bind to the promoter region of the target gene, and the transcriptional activator module interacts with the dCas9 protein to enhance the transcriptional activity of the gene, thereby achieving the activated expression of the gene. Based on the principles of the SAM system,^[^ ^42^ ^]^ SgRNA‐dCas9 lentiviruses targeting the *RPS6KA2* transcription initiation site (TSS) were constructed and successfully upregulated *RPS6KA2* expression (Figure 9d–f). With increased *RPS6KA2* expression, the levels of hepatic functional genes (*ALB* and *ARG*) and drug metabolism enzyme genes (*CYP3A4* and *CYP2D6*) decreased. The expression of *HNF4A* decreased by nearly three fold, whereas stemness indices, including *POU5F1* (120‐fold) and *EPCAM* (10 fold), significantly increased (Figure 9b). To further clarify the regulation of *RPS6KA2* on hepatic differentiation, RPS6KA2‐siRNA was transfected into *RPS6KA*2‐overexpressing HepaRG cells, which led to a reduction in RPS6KA2 expression (Figure 9g). This resulted in the restoration of hepatic functional gene expression, including that of *ALB*, *AAT*, *KRT18*, *ARG*, *CYP3A4*, *CYP1A2*, *UGT1A1*, and *HNF4A*, whereas stemness markers (*POU5F1* and *EPCAM*) were significantly downregulated (Figure 9b). Collectively, these findings indicate that RPS6KA2 plays a critical role in regard to negatively regulating the hepatic differentiation of HPCs.

## 3 Discussion

HPCs are the transitional cells formed between cholangiocytes and hepatocytes. Previous research, along with our own study, has demonstrated that in cirrhotic liver tissues, HPCs with bipotential differentiation capacity are significantly activated.^[^ ^43^, ^44^ ^]^ They play significant roles in the regeneration of liver cell regeneration.^[^ ^3^, ^45^, ^46^, ^47^, ^48^, ^49^, ^50^ ^]^ However, the regulation of hepatocyte differentiation and maturation remains unclear. There is a close relationship between the intestine and the liver. The intestine absorbs nutrients, medications, and toxins from food and transports them to the liver through the portal vein.^[^ ^51^ ^]^ Therefore, the intestines affect the liver microenvironment. However, it is unclear whether it regulates the hepatic differentiation of HPCs. An ideal model that replicates the fundamental structures of the gut and liver and their relationships is required to address this question.

The two primary factors that determine cell models are cell type and cell culture mode. The cell‐line culture model has advantages such as easy accessibility, low cost, easy handling, and fast turnaround time. HepaRG cells were isolated from an Edmonson‐grade I‐differentiated liver tumor that developed into macronodular cirrhosis resulting from long‐term chronic HCV infection. The HepaRG cell line is a human oval ductular bipotent hepatic progenitor cell line that coexpresses both hepatocyte and cholangiocyte.^[^ ^52^ ^]^ Primary hepatocytes are terminally differentiated and have limited potential for hepatic differentiation. Therefore, compared to primary hepatocytes, HepaRG cells were more suitable for the investigation in this study. The human epithelial cell line Caco‐2 is widely used as a model of the small intestine and was selected as the cell line representing intestinal epithelial cells.^[^ ^53^, ^54^, ^55^, ^56^ ^]^ Organoids derived from human tissues (intestine and liver) were used in this study as they are more representative of the in vivo biological characteristics of human organs, such as tissue heterogeneity, intricate cell‐cell and cell‐matrix interactions, and tissue‐specific functions.^[^ ^11^, ^57^ ^]^ The cell culture mode refers to the specific approach or system used to culture cells, whether it is a traditional 2D cell culture, a 3D organoid culture model, a Transwell system, or a microfluidic organ‐on‐a‐chip system. The choice of the culture mode significantly affects how well the in vitro model replicates the natural behavior, structure, and function of cells or tissues in vivo. Microfluidic organs‐on‐a‐chip provide a more realistic tissue microstructure, better simulate the in vivo dynamic culture environment, and more accurately recapitulate the physiological and pathological status of cells.^[^ ^58^ ^]^ Furthermore, compared with a static Transwell system, microfluidic chips can offer a more direct assessment of the influence of one organ on another. To provide a better model that can capture the direct effects of the intestine on the hepatic differentiation of HPCs, a gut‐liver‐on‐a‐chip was designed and fabricated in this study. When compared to other organ‐on‐a‐chip platforms,^[^ ^59^, ^60^ ^]^ our gut‐liver‐on‐a‐chip platform was designed to incorporate more biomimetic features. It not only recapitulates the specific spatial 3D structure of both the intestine and liver but also enables the integration of organoids derived from primary adult tissues of both the intestine and liver into an organ‐on‐a‐chip.

Using the gut‐liver‐on‐a‐chip in this study, we observed an improved intestinal barrier and a higher hepatocyte‐like function in the cell lines and organoids under perfused conditions. Moreover, organoids on the chip exhibited superior functional protein expression and drug metabolism compared with cell lines, suggesting that they closely simulate in vivo functions. Notably, coculture with intestinal epithelial cells significantly enhanced the hepatic differentiation of HPCs, with greater effects observed in the chip than in the Transwell models. Coculture with intestinal epithelial cells (either in cell lines or organoid systems) significantly enhanced the hepatic differentiation of HPCs, as evidenced by the upregulation of hepatocyte markers (e.g., ALB, AAT, and HNF4A) and downregulation of stemness markers (e.g., AFP and POU5F1). The assessment of CYP450 enzyme expression and activity is also instrumental in evaluating liver function. In this study, CYP2D6, CYP3A4, and CYP1A2 were selected owing to their pivotal roles in metabolism and extensive involvement in the processing of a broad spectrum of clinically relevant drugs. CYP3A4 is the most abundantly expressed CYP in the liver, accounting for ≈30–40% of the total CYP content.^[^ ^61^ ^]^ CYP1A2, another crucial hepatic metabolizing enzyme, accounts for ≈13% of all CYP.^[^ ^62^ ^]^ CYP2D6 is a key drug‐metabolizing enzyme involved in the metabolism of ≈20% of the commonly used drugs.^[^ ^63^ ^]^ Our findings revealed that all three enzymes exhibited higher activity under co‐culture conditions compared to mono‐culture conditions in this study. These results support the vital role of intestinal epithelial cells in promoting the hepatic differentiation of HPCs. Notably, these effects were more pronounced in the microfluidic chip system than in the Transwell model, thus underscoring the superior ability of organ‐on‐a‐chip platforms to replicate complex cellular interactions.

Exosomes are small vesicles (30–100 nm) secreted into the extracellular space by nearly all cell types and mediate various forms of cell‐to‐cell communication.^[^ ^64^ ^]^ Our study demonstrated that the addition of EXOs (derived from intestinal epithelial cell lines and organoids) to HPC cultures enhanced hepatocyte‐specific markers and functions (including ALB, ALB, KRT18, HNF4A, urea, and glycogen synthesis) and the acquisition of hepatocyte‐like morphology. These data support their role in hepatic differentiation of HPCs as a critical mechanism for regulating HPCs fate.

Building on these in vitro findings, we extended our investigation to an in vivo model to explore the potential therapeutic efficacy of exosomes derived from Int‐Orgs for the treatment of liver fibrosis. Given the distinct advantages of human‐derived biological products, specifically their low immunogenicity and absence of zoonotic pathogen contamination, which are highly desirable characteristics for future clinical applications in liver diseases, we opted to use human‐derived Int‐Orgs EXOs in our in vivo study. The EXOs isolated from human Int‐Orgs were administered to mice with liver fibrosis induced by CCL4. After 2 or 5 weeks of EXO treatment, mice with liver fibrosis showed increased body weight and decreased fibrotic liver area. EXOs treatment improves liver function in mice with liver fibrosis. The TBIL, DBIL, ALT, and AST levels decreased, whereas the ALB, AAT, and BUN levels increased. Decreased expression of alpha‐fetoprotein (AFP), a marker, was observed in the EXO‐treated group. These findings suggested that EXOs from Int‐Orgs may alleviate the progression of liver fibrosis by promoting hepatic differentiation. This discovery not only deepens our understanding of the biological functions of exosomes in liver diseases but also opens new avenues for the development of potential therapeutic strategies for liver fibrosis.

EXOs have been shown to contain many different cargo molecules, among which miRNAs are the most abundant.^[^ ^65^ ^]^ Additionally, EXOs offer protection to miRNAs in harsh environments, allowing their stable presence in human plasma and bodily fluids.^[^ ^64^ ^]^ To identify the molecular mechanisms underlying the pro‐differentiation effects of intestinal EXOs, we analyzed the miRNAs in EXOs. Among the miRNAs enriched in intestinal EXOs, the miR‐371‐373 cluster has emerged as a key regulator of HPC differentiation. These miRNAs were found to target RPS6KA2, a negative regulator of hepatic differentiation, and their overexpression in HPCs led to the increased expression of hepatocyte markers. Conversely, the inhibition of the miR‐371‐373 cluster reversed the pro‐differentiation effects of intestinal EXOs, confirming their critical roles in this process. The miR‐371‐373 cluster is specifically expressed in human cells, whereas the homologous cluster in mice is miR‐290‐295.^[^ ^66^ ^]^ Moreover, the expression level of the miR‐372‐3p and miR‐373‐3p has been found to be downregulated in NAFLD (non‐alcoholic fatty liver disease) patients with fibrosis compared to those with normal histology.^[^ ^67^ ^]^ A past clinical study also reported that miRNA‐372 is downregulated in hepatocellular carcinoma.^[^ ^68^ ^]^ Collectively, our findings suggest the crucial regulatory role of the miR‐371‐373 cluster in advanced liver disease. Advanced liver disease is frequently accompanied by intestinal dysfunction in clinical patients.^[^ ^69^ ^]^ We have hypothesized that the impairment of intestinal function in patients with liver fibrosis might lead to a diminished amount of the miR‐371‐373 cluster entering the liver via EXOs, thereby preventing HPCs from differentiating into hepatocytes. This, in turn, may exacerbate liver repair during disease progression. Our study suggests that restoring normal communication between the intestine and the liver through external intestinal‐derived exosomes may offer a promising therapeutic strategy for advanced liver diseases. However, further research is needed to confirm this hypothesis and to shed light on the complex interplay between the miR‐371‐373 cluster, intestinal function, and advanced liver diseases.

RPS6KA2, a member of the ribosomal S6 protein kinase family, is associated with various substrates, including those involved in the MAPK*/*ERK pathway.^[^ ^70^, ^71^, ^72^, ^73^ ^]^ Notably, a previous study established that RPS6KA2 functions as a key regulator of hepatic differentiation.^[^ ^74^ ^]^ A dual‐luciferase reporter gene assay confirmed that *RPS6KA2* is a common target of miR‐371a‐5p/372‐3p/373‐3p. The negative regulatory effect of RPS6KA2 was confirmed using RPS6KA2 siRNA and sgRNA. Based on these findings, we propose that the miR‐371‐373 cluster–RPS6KA2 signalling pathway in EXOs from intestinal epithelial cells may mediate the hepatic differentiation of HPCs. Analysis of PPI results suggested an indirect interaction between RPS6KA2 and HNF4A (Figure S7k, Supporting Information). According to previous studies,^[^ ^75^ ^]^ CREB2 (ATF4, the primary transcription factor for RPS6KA2) serves as a bridge between RPS6KA2 and HNF4A. A reduction in the activation of RPS6KA2 results in a decrease in the serine‐133 phosphorylation level of CREB2.^[^ ^73^ ^]^ CREB2 (ATF4) serves as a transcriptional co‐activator of HNF4A. When CREB2 is significantly negatively expressed, the expression level of HNF4A correspondingly increases.^[^ ^75^ ^]^ Given that HNF4A has been confirmed as the core regulator of hepatic differentiation,^[^ ^76^ ^]^ we can therefore hypothesize that RPS6KA2 might exert regulatory effects on hepatic differentiation by interacting with CREB2 and HNF4A. Furthermore, the miR‐371‐373 cluster within EXOs from intestinal cells might alleviate liver fibrosis by promoting the hepatic differentiation of HPCs via RPS6KA2 and its interaction with CREB2 and HNF4A. However, further investigations are required to validate these hypotheses.

This study had some limitations. First, there is inherent variability in the generation of organoids, which is influenced by multiple factors, such as cell sources, culture environment, and donor characteristics. Second, although the gut‐liver chip model successfully simulated the interaction between organs, it is still challenging to eliminate potential variations owing to the complexity of the fabrication process. Additionally, because of the high cost of organoids‐on‐a‐chip, this technique has not been used to evaluate the function of the miR‐371‐373 cluster and its downstream targets in the regulation of HPCs differentiation. The limited cell culture capacity also constrains the use of certain conventional experimental techniques, such as western blotting. Furthermore, the potential influence of the intestinal microbiome on in vivo experimental results could not be fully accounted for. Finally, scaling up the production of Int‐Orgs EXOs for clinical use still faces challenges, considering the difficulties in obtaining primary intestinal tissues and also the high cost of large‐scale production.

## 4 Conclusion

Our gut liver‐on‐a‐chip models have demonstrated that intestinal epithelial cells can facilitate the hepatic differentiation of HPCs through their EXOs. We have shown that treatment with exosomes derived from intestinal organoids substantially ameliorated liver fibrosis in CCL4‐induced mouse models. It is possible that the miR‐371‐373 cluster–RPS6KA2 signalling pathway in EXOs originating from intestinal epithelial cells is responsible for this effect. These findings provide a new perspective for the future treatment of advanced liver diseases.

## 5 Experimental Section

### Culture of Organoids—Patients and Clinical Specimens

Normal human duodenal tissue was obtained from a patient with gastric cancer after surgical resection at the Zhujiang Hospital. Human liver fibrosis samples were obtained from adjacent non‐tumorous tissues of a patient with liver cancer who underwent surgical resection at Zhujiang Hospital. Specimens were collected with informed consent from the patients during surgery, and ethics committee approval was obtained from the Zhujiang Hospital, Southern Medical University (approval number: 2022‐KY‐243‐01). Details of the donors are presented in Table S5 (Supporting Information).

### Culture of Organoids—Isolation of Human Intestinal Crypt Cells and Culture of Int‐Orgs

Human primary intestinal crypt cells were isolated following previously described procedures, with some modifications.^[^ ^77^ ^]^ Briefly, the duodenal samples were transferred to cold DMEM‐F12 (with 15 mm HEPES, Stemcell, Canada, Catalog #36254). The luminal side of the intestine was scraped to remove the luminal content and villous structures. After the intestine was gently washed three times with ice‐cold PBS, it was cut into 1–3‐mm pieces with scissors. The pieces were transferred to a tube and further washed with cold PBS (5–10 times). Tissue pieces were resuspended in 10 mL of Gentle Cell Dissociation Reagent (GCDR, Stemcell, Canada, Catalog: #100‐0485) and incubated on ice for 30 min on a shaker. The supernatant was removed by centrifugation at 290 × g for 5 min. The tissue pieces were resuspended in 10 mL of cold (2–8 °C) DMEM‐F12 containing 1% BSA and pipetted up and down three times. Most intestinal pieces were allowed to settle at the bottom (≈30 s). The supernatant was collected and passed through a 70 µm strainer to obtain the crypt fraction. Crypts were suspended in a 1:1 mixture of Human IntestiCult Organoid Growth Medium (IGM, Stemcell, Canada, Catalog: #06010) and cytokine‐free Matrigel (Corning, Japan, Catalog: 354230) and added to preheated 24‐well plates (50 µl per well). After incubation at 37 °C for 20 min, IGM containing 10 µM Y‐27632 (Stemcell, Canada, Catalog #72302) was added to maintain the culture. The medium was changed every two days. The outgrowing crypts were passaged once a week, and the organoid stocks were maintained for up to 5 months.

### Culture of Organoids—Isolation of Human HPCs and Culture of HPC‐Orgs

Liver tissue was rinsed with HepatiCult Organoid Basal Medium (Stemcell, Canada, Catalog: #100‐0387). The tissue was cut into 1 mm^3^ pieces using scissors. The liver pieces were washed in 10 mL of HepatiCult Organoid Basal Medium containing 1% FBS 10 times, and the supernatant was discarded. Preheated TM Tissue Digestible Solution (BioGenous, China, Catalog: K601008) was added and incubated at 37 °C in a 200‐rpm shaker for 20 mins. The digestion was terminated, and the supernatant was transferred to a conical tube. The digestion cycle was repeated until the tissue pieces completely dissociated into the hepatic ducts and no tissue pieces remained. The collected supernatants were centrifuged at 300 × g for 3 min. Hepatic progenitor cells (HPCs) were obtained using FACS (see flow cytometry analysis for details). The supernatant was discarded, and the pellet was then resuspended in a 1:1 mixture of human HepatiCult Organoid Growth medium (HGM, Stemcell, Canada, Catalog: #100‐0385) and cytokine‐free Matrigel. The supernatant was transferred to a single well of a 24‐well plate to form a dome. After the domes solidified, 750 µL of HGM containing 10 µM Y‐27632 was added for maintenance culture. The culture medium was changed every four days. The cells were considered ready to pass when they resembled organoids. Primary human hepatocytes (PHHs) and primary human intrahepatic bile duct cells (PHBDs) were obtained from primary normal liver tissues by tissue enzymolysis and cell sorting (KRT18^+^PHHs, KRT19^+^PHBDs).

### Culture of Organoids—Differentiation of Int‐Orgs and HPC‐Orgs

Int‐Orgs that were previously cultured with Human IntestiCult Organoid Growth Medium were switched to Human IntestiCult Organoid Differentiation Medium (IDM, Stemcell, Canada, Catalog: #100–0214) containing 5 µM DAPT (Stemcell, Canada, Catalog: #72082) and maintained for 7 days. For the first‐stage differentiation of HPC‐Orgs, HDM‐I (HGM with 10 ng mL^−1^ oncostatin M (Stemcell, Canada, Catalog: #78094) and 1 µm dexamethasone (Sigma, USA, Catalog: D4902)) was added and maintained for 5 days. For the second stage of differentiation, HDM‐II (Human HepatiCult Organoid Differentiation Medium, HDM, Stemcell, Canada, Catalog: #100‐0383) was added and maintained for 10 days.

### Cell Culture and Differentiation

The colon cancer cell line (Caco‐2) was purchased from ATCC (Manassas, VA, USA). HepaRG cells were purchased from Thermo Fisher Scientific (Waltham, MA). Caco‐2 cells were cultured in MEM (BasalMedia, China, Catalog: L540KJ) supplemented with 20% FBS (Gibco, USA, Catalog: 10091148), 100 U mL^−1^ penicillin, and 0.1 mg mL^−1^ streptomycin (BasalMedia, China, Catalog: S110JV). The maintenance medium for HepaRG cells was William's E medium (Gibco, USA, Catalog: A1217601) supplemented with HepaRG general purpose medium supplement (Thermo Fisher, USA, Catalog: HPRG670), 1X GlutaMAX (Thermo Fisher, USA, Catalog: 35050061), 100 U mL^−1^ penicillin and 0.1 mg mL^−1^ streptomycin. All cells were cultured in a humidified incubator at 37 °C and 5% CO2. For differentiation into HepaRG cells, they were seeded at a density of more than 90% and maintained in culture for 5 days. Subsequently, the cells were transferred to differentiation medium and maintained for 10 days. The differentiation medium for HepaRG cells was cultured in maintenance medium supplemented with 2% DMSO (MP Biomedicals, China, Catalog: 196055).

### Cell Counting Kit‐8 Assay

Cell growth and relative quantification were performed using the Cell Counting Kit‐8 (CCK8) assay. Prior to establishing the coculture system, a series of cell growth tests were conducted to determine the optimal medium. Caco‐2 and HepaRG cells were seeded separately in 96‐well plates at a density of 800 cells/well. Different ratios of MEM and Williams’ E complete medium (ranging from 1:0 to 0:1) were added to the wells, and the cells were cultured for 10 days. At each time point (2, 4, 6, 8, and 10 days), 100 µL of DMEM containing 10 µL of CCK8 solution (Dojindo, China, Catalog: LH646) was added to each well and incubated at 37 °C for 2 h. The absorbance of the wells was measured at 450 nm using a microplate reader (Thermo Fisher Scientific). Cell growth curves were plotted using the GraphPad Prism 9 software. A 2:1 ratio of MEM to Williams' E complete medium was determined to be the optimal coculture medium for HepaRG and Caco‐2 cells. (Figure S1c, d, Supporting Information)

To quantify HepaRG cells on the chip, HepaRG cells (mixed with Matrigel) were 3D cultured in 96‐well plates with initial seeding numbers of 1000, 2000, 4000, 8000, and 16 000. Caco‐2 cells of the same number were seeded in 96‐well plates coated with Matrigel. After 4 h of cell adherence, the absorbance of each well was measured using a CCK8 assay. Standard curves were then generated to quantify the number of HepaRG and Caco‐2 cells. HepaRG and Caco‐2 cells were seeded separately onto the chips. 100 µL of DMEM containing 10 µL of CCK8 solution was added to the chips and incubated at 37 °C for 4 h. The perfusate was collected, and light absorption was measured in order to calculate the number of HepaRG and Caco‐2 cells on the chips based on their corresponding standard curves. Enumeration of Hep‐Org and Int‐Org cells on the chips followed procedures analogous to those used for cell lines.

### Construction of the Coculture Systems of Cell Lines and Organoids—Determination of a Coculture Medium for Hep‐Orgs and Int‐Orgs

To determine the optimal coculture medium for Int‐Orgs and HPC‐Orgs, both cell types were individually cultivated in a range of mixed media compositions (IGM: HGM ratios of 1:0, 3:1, 2:1, 1:1, 1:2, and 0:1). Cell growth curves were used to assess the cellular activity in different media blends using the CCK8 assay. Lineage differentiation of Int‐Orgs was assessed by qRT‐PCR, whereas differentiation of Hep‐Orgs was determined by measuring the levels of secreted ALB by ELISA. Following the growth and differentiation assessments of both organoid types, the expansion coculture system was designated as IGM: HGM (2:1). The culture system was then replaced with IGM: HDM‐I (2:1) to ensure initial differentiation of HPC‐Orgs while minimizing the impact on Int‐Orgs. Subsequently, the differentiation coculture medium was adjusted to an IDM: HDM‐II (1:1). The results of the co‐culture medium experiments are shown in Figure S5 (Supporting Information).

### Construction of the Coculture Systems of Cell Lines and Organoids—Construction of a Static Coculture System of Cell Lines and Organoids

To create a static coculture system, Transwell chamber inserts with a 0.4‐µm aperture (Corning, Japan, Catalog: #^34^70, #3450) were used in 6‐well or 24‐well plates. The membrane was coated with Matrigel at 100 µg mL^−1^. HepaRG cells differentiated for 10 days were mixed with Matrigel (10 mg mL^−1^) and seeded at the bottom of the plate. Caco‐2 cells were seeded onto the membranes of the chamber inserts. The coculture medium for HepaRG/Caco‐2 cells was MEM: Williams’ E at a ratio of 2:1. For the construction of the coculture system for organoids, polystyrene rings were used to increase the height of the bottom chamber to accommodate the growth of the 3D organoids. HPC‐Orgs mixed with the Matrigel solution were seeded in the bottom layer, whereas undifferentiated Int‐Orgs were seeded in the upper layer. The Transwell plate was then placed at 37 °C with 5% CO2 for 20 min to allow the Matrigel to solidify. The coculture system was maintained in an expansion medium (IGM: HGM at a ratio of 2:1) for 4 days. After completing the transition stage with an IGM: HDM‐I ratio of 2:1, the medium was switched to a differentiation coculture system with an IDM: HDM‐II ratio of 1:1. These coculture media were also used in the monoculture control group for comparative purposes.

### Establishment of gut‐Liver‐on‐a‐Chip Using Cell Lines or Organoids—Design and Fabrication of Microfluidic Equipment

The microfluidic gut‐liver‐on‐a‐chip was designed using SolidWorks 2020 software. The chip was fabricated by the Wuhan Siyi Service Co., Ltd. (China). The microfluidic chip was cast using polydimethylsiloxane (PDMS, Japan). The necessary microfluidic design was photolithography‐patterned on a silicon wafer using an SU‐8 photoresist (SU‐8 2100, Japan). The PDMS base and its curing agent were mixed at a ratio of 10:1 (w/w) and poured onto a silicon wafer, which acted as the master template. The mixture was then degassed in a vacuum chamber for 20 mins and cured in an oven at 80 °C for 30 min. The cured PDMS was then carefully removed from the mold. The microfluidic chip was composed of three layers and two chambers (intestinal and liver). The top layer contains the inlets and outlets. The intestinal culture chamber was situated in the left middle layer with a lateral wavy shape that mimicked the structure of the native crypt‐ and villus‐like domains. A 10 µm thick translucent polycarbonate membrane (Corning, Japan) was embedded between the middle and bottom layers, precisely positioned beneath the intestine culture chamber. The liver chamber was designed to recapitulate the microstructure of hepatic sinusoids. Micropillars were created to separate the cells from the medium. Additionally, a microtube was incorporated as a vessel‐like structure connecting the intestinal and liver chambers. By utilizing an oxygen plasma treatment, the PDMS layers and glass slides were bonded together to form a sealed microfluidic chip.

Before the experiment, the chips were sterilized under ultraviolet irradiation. Stainless‐steel plugs and polytetrafluoroethylene (PTFE) tubing were inserted into the inlets and outlets. A hepatic sinusoid‐like culture system was constructed to establish a gut‐liver on‐chip system. HepaRG cells (1 × 10^6^ cells mL^−1^) mixed with Matrigel (10 mg mL) were injected into both the left and right liver chambers and incubated at 37 °C for 20 min. Matrigel diluent (300 µg mL) was injected into the intestine channel, and the chip was placed at 37 °C for 20 min. Subsequently, the Caco‐2 cells (2 × 10^6^ cells mL^−1^) were injected into the intestinal chamber and incubated under static conditions for 4 h to facilitate cell adhesion. Nonadherent cells were washed away, and the liver chamber and intestine chamber were perfused with coculture medium (MEM: Williams’ E at a ratio of 2:1) at a flow rate of 1 µL min^−1^.

### Establishment of Gut‐Liver‐on‐a‐Chip Using Cell Lines or Organoids—Construction of a Gut‐Liver‐on‐a‐Chip co‐Culture System

Undifferentiated Int‐Orgs and HPC‐Orgs were prepared by mechanical disruption and digestion using GCDR. As illustrated in Figure S5a (Supporting Information), for the construction of a gut‐liver‐on‐a‐chip co‐culture system, undifferentiated Int‐Orgs (passage 6) and undifferentiated HPC‐Orgs (passage 4) were chosen. Initially, 200 µL of Int‐Orgs Matrigel suspension was injected into the intestine chamber via the intestine inlet. The intestinal channel was maintained with HGM, and the culture was carried out for 2 days at a flow rate of 1 µL min^−1^. Following this, 80 µL of the HPC‐Orgs Matrigel suspension was seeded into the liver chambers. HGM was continuously utilized to sustain the growth of Int‐Orgs, while IGM was introduced into the central channel to maintain HPC‐Orgs, with the culture proceeding for an additional 4 days. Once the intestinal barrier integrity exceeded 70%, the culture medium in all channels was substituted with IGM: HDM‐I (2:1) to maintain the growth of Int‐Orgs and induce the differentiation of HPC‐Orgs for 5 days. Subsequently, the culture medium in all channels was replaced with IDM: HDM‐II (1:1) for the differentiation of Int‐Orgs and HPC‐Orgs, with the culture being maintained for another 6 days. In the HPC‐Orgs monoculture group, the culture medium regimen and perfusion rate were identical to those employed in the co‐culture group.

### Establishment of Gut‐Liver‐on‐a‐Chip Using Cell Lines or Organoids—Assessment of Intestinal Barrier Permeability

FITC‐dextran (40 kDa, Sigma, USA, Catalog: 60842‐46‐8) solution at a concentration of 50 µg mL^−1^ was injected into the intestine chamber of the chip and perfused at a rate of 100 µL h^−1^. Effluents from the gut and medium chambers were collected every 3 h, followed by centrifugation at 1000 × g for 5 min. The supernatant was collected and transferred to a black 96‐well plate. Using the fluorescence detection module of a multifunction microplate reader (Thermo Fisher, USA), the excitation wavelength was adjusted to 488 nm, and the emission wavelength was 525 nm to detect the fluorescence intensity of the medium channel effluent (Fluo_medium_) and gut chamber effluent (Fluo_Epi_). The ratio of Fluo_medium_/Fluo_Epi_ was calculated, and the permeability curve was drawn using GraphPad Prism 9 software.

### Establishment of Gut‐Liver‐on‐a‐Chip Using Cell Lines or Organoids—RNAs Isolation and Quantitative Real‐Time qRT‐PCR

Total RNA was extracted from the EXOs using a Total Exosome RNA and Protein Isolation Kit (Thermo Fisher, USA, Catalog: 4478545) according to the manufacturer's instructions. RNA from cells and organoids was extracted using TRIzol reagent (Thermo Fisher, USA, Catalog: 15596018) and quantified using a Nanodrop2000 (Thermo Fisher, USA). For RNA extraction from the cells on the chip, trypsin‐EDTA was injected for digestion for 5 min, and the non‐gel‐coated cells on the chip were removed. Cell Recovery Solution (CRS, Corning, Japan, Catalog: 354253) was then infused into the chip and incubated on ice for 1 h. Cells on the chip were obtained by infusing the channel with PBS. For mRNA analysis, cDNA was synthesized using an mRNA reverse transcription reagent (AG, China, Catalog: AG11711). For miRNA analysis, cDNA was synthesized using the miRNA reverse transcription reagent (AG, China, Catalog: AG11717). cDNA was quantified by real‐time PCR using SYBR Green qPCR Master Mix (AG, China; Catalog: AG11701). The mRNA and miRNA expression levels were normalized to the expression levels of GAPDH and U6, respectively. The 2^−△△CT^ method was used to calculate the expression levels. The primer sequences for the mRNAs and miRNAs are shown in Tables S1–S 3 (Supporting Information).

### Establishment of Gut‐Liver‐on‐a‐Chip Using Cell Lines or Organoids—Flow Cytometry and Fluorescence Activating Cell Sorter (FACS) Analyses

For cells or organoids on chips, GCDR was injected into the channel, and the Hep‐Orgs on chips were digested at room temperature (RT) for 15 min. The cells were collected from the chip, centrifuged at 290 g for 5 min, and washed twice with PBS. The eBioscience Foxp3/ transcription factor flow fixation/permeabilization buffer (eBioscience, USA, Catalog: 00‐5523‐00) was then added to cell suspension, followed by overnight fixation at 4 °C. Human BD Fc Block (BD Biosciences, USA, Catalog: 564219) was used to prevent the non‐specific binding of antibodies to Fc receptors. Subsequently, the primary antibodies were incubated at RT for 1 h, and the secondary antibodies were incubated at RT for 30 min. The intracellular protein fluorescence intensity was measured using flow cytometry (BD Biosciences, USA), and data analysis and image integration were performed using the FlowJo software.

### Establishment of Gut‐Liver‐on‐a‐Chip Using Cell Lines or Organoids—ALB, AAT, AFP, and CYP3A4 Detected by ELISA

The supernatant samples were collected at various time points. The concentrations of ALB, AAT, and AFP in the supernatants were measured using ALB ELISA kits (Abcam, Cambridge, UK, Catalog: ab179887), AAT ELISA kits (Abcam, UK, Catalog: ab108799), and AFP ELISA kits (Abcam, Cambridge, UK, Catalog: ab108838), respectively. The content of CYP3A4 in the cells was determined using a CYP3A4 ELISA kit (MEMIAN, China, Catalog: MM‐2358H2) following the manufacturer's instructions. The number of cells was used for standardized quantification.

The level of urea in the supernatant was tested using a Urea Colorimetric Assay Kit (Jiancheng, China, Catalog: C013‐1‐1), following the manufacturer's instructions.

### Establishment of Gut‐Liver‐on‐a‐Chip Using Cell Lines or Organoids—Activity of CYP450s Measured by HPLC‒MS

The activity of CYP450s of the differentiated HepaRG cells and HPC‐Orgs in the monoculture and coculture systems was evaluated by measuring the corresponding metabolites using HPLC‒MS. Four specific substrates of CYP450s were chosen: phenacetin (Yuanye, China, Lot NO. Y05M7Y308689) for CYP1A2, and dextromethorphan (Yuanye, China; lot no. Y03S11W120802) for CYP2D6 and testosterone (Apexbio, USA, Lot NO. C6163213416B9) for CYP3A4. To ensure accurate metabolite detection throughout the HPLC‐MS analysis, the PDMS chip was pretreated with complete medium containing 2% bovine serum albumin (BSA) for 12 h to minimize nonspecific drug adsorption. This optimized protocol demonstrated a marked reduction in drug retention by the PDMS material, with an absorption efficiency of < 5%. Then, three drugs, each at a concentration of 50 µM, were added respectively to the chip system with cells, and supernatant was collected every 24 h.

The LC‐MS/MS system consisted of a triple‐quadrupole mass spectrometer (AB/SCIEX 4000 QTRAP; SCIEX, Concord, Ontario, Canada) equipped with an electrospray ionization interface coupled to an LC system (LC‐30AD XR; Shimadzu). The LC system was equipped with two pumps, a system controller, an autosampler, a column oven, and an online degasser. Chromatographic separation was carried out at RT on a Phenomenon Kinetex XB‐C18 column (2.1 × 100 mm, 2.6 µm) at a flow rate of 0.4 mL min^−1^. The temperature of the autosampler was 4 °C, and the injection volume was 5 µL. The mobile phases were composed of 0.1% formic acid and 2 mmol L^−1^ ammonium formate in water (solution A) and methanol (solution B) with a gradient elution as follows: 0–1.0 min, 5% B; 1.0–2.0 min 5–95% B; 2.0–4.5 min, 95% B; and 4.5–4.6 min, 95–5% B. 5% B was then maintained at 4.6–7.0 min to reach equilibrium. The total runtime was 7.0 min. The mobile phase effluent from the column was diverted to waste before 0.5 min and after 6.50 min. Electrospray ionization (ESI) was performed in the positive ion mode with nitrogen as the nebulizer, turbo spray, and curtain gas, with the optimum values set at 50, 50, and 10 psi, respectively. The turbo gas temperature was set at 500 °C, and the ESI needle voltage was adjusted to 5500 V. The MRM transitions were m/z 152.1 → 110.1, m/z 258.2 → 157.1, m/z 312.2 → 266.1, and m/z 305.4 → 269.2 for acetaminophen (APAP), dextrophan (DXO), and 6β‐hydroxytestosterone (6βH‐TEST), respectively. Data acquisition, data processing, and instrument control were performed using Analyst V1.6.3 (SCIEX, Concord, Ontario, Canada). To eliminate the interference of CYP450s in the intestinal cells, the level of metabolites in hepatocytes in the gut‐liver coculture system was equal to the concentration of metabolites in the coculture system minus the concentration of metabolites in the monocultured intestinal cell system.

### Establishment of Gut‐Liver‐on‐a‐Chip Using Cell Lines or Organoids—RNA Sequencing

For the Hep‐Orgs on the chip and HepaRG on the chip, the mRNA extracted from each group of cells from 10 chips was sequenced using next‐generation mRNA sequencing (mRNA‐seq). An Illumina HiSeq NextSeq 2000 instrument was used for sequencing, and a sequencing library was constructed. The FPKM value was employed to quantify the gene expression levels. The DESeq R package was used for differential expression analysis. The corrected p value was set to 0.05, and the absolute value of log 2 FC (fold change) was set to ≥0.6 as the threshold for differentially expressed genes (DEGs). The DEGs were further analyzed based on their biological and molecular functions. R packages, including “clusterProfiler,” “enrichplot,” “org. Hs.eg.db,” “pathview,” and “ggplot2,” were used to perform Gene Ontology (GO) database (http://www.geneontology.org/) and Kyoto Encyclopedia of Genes and Genomes (KEGG) database (http://www.kegg.jp/) pathway enrichment analysis. For GO enrichment analysis, biological process (BP), molecular function (MF), and cellular component (CC) categories were analyzed. A threshold of p < 0.05 was used. The intersection of the two groups of DEGs between HepaRG and Hep‐Orgs was then taken, and overlapping DEGs with the same trend were selected for pathway enrichment analysis. The SangerBox data analysis platform (http://sangerbox.com/) was used to draw chord graphs and volcano and Venn diagrams. STRING (https://www.string‐db.org/) was used to construct protein‐protein (PPI) networks of proteins encoded by DEGs. A combined score > 0.6 was considered significant, and key genes with a degree >20 were chosen. All of the RNA sequencing data were accessible through SRA PRJNA1145173 in the NCBI database.

### Establishment of Gut‐Liver‐on‐a‐Chip Using Cell Lines or Organoids—Immunofluorescence Staining, HE, PAS Staining, and MASSON Trichrome Staining

Immunofluorescence experiments on cell lines cultured in Transwell plates were conducted according to the manufacturer's instructions. The supernatant was removed, and the cells were washed three times with PBS. The cells were fixed in 4% paraformaldehyde for 15 min. The cells were permeabilized in PBS containing 0.2% Triton‐X‐100 at room temperature (RT) for 30 min. The samples were washed three times with PBS and then transferred into immunofluorescence staining blocking solution (Beyotime, China, Catalog: P0102), followed by incubation with primary antibodies diluted in blocking solution overnight at 4 °C. The samples were washed with PBS three times, and fluorescent secondary antibodies were added and incubated at RT for 1 h, followed by nuclear staining with DAPI (Beyotime, China, Catalog: C1006) for 20 min. The images were captured using a fluorescence microscope. For immunostaining cell lines on the chip, solutions were perfused onto the chip using a microinjection pump at a flow rate of 0.1 mL min^−1^. The chip was then filled with 4% paraformaldehyde, fixed for 30 min, and then washed with PBS for 15 min. PBS containing 0.2% Triton X‐100 was added, and the mixture was allowed to permeate at RT for 45 min. The samples in the chip were blocked with an immunofluorescence sealing solution at RT for 2 h. Primary antibodies were added and incubated at 4 °C overnight. After washing with PBS for 30 min, the cells were incubated with the fluorescent secondary antibody at RT in the dark for more than 2 h. DAPI was infused for nuclear staining for 30 min. The bottom slice of the chip was then removed.

To perform immunofluorescence staining on the organoids cultured in Transwell plates, a solution of precooled 4% paraformaldehyde was added and incubated for 30 min. Organoids were mechanically dissociated by pipetting. For organoids‐on‐a‐chip, a collection of 8–10 chips containing organoids was washed three times with PBS. Next, the organoids were coated with a 2% agarose solution and embedded in paraffin. After pre‐cooling the paraffin, make slices with a thickness of 4 µm and place them in a 37 °C oven. In the immunofluorescence experiments on organoid slices, microwave antigen repair was performed using a citrate solution (pH 6.0). The slices were permeabilized with 0.2% Triton X‐100 for 30 min and then blocked with 2% goat serum at RT for 1 h. Primary antibodies were added and incubated at 4 °C overnight, and fluorescent secondary antibodies were added and incubated at RT for 1 h. DAPI was used for nuclear staining. For HE staining, the sections were stained with H&E for 5 and 2 min, respectively. Images were captured using a SP8 laser confocal microscope (Leica, Germany) and an Axiovert A1 inverted fluorescence microscope (Zeiss, Germany). The 3D reconstructed images were obtained using an SP8 laser confocal microscope. Images were processed and analyzed using Photoshop 2020 or ImageJ software. Excitation light intensity, gain, and exposure time were consistent across the groups for the same metric. Information on the antibodies used is shown in Table S6 (Supporting Information).

For PAS detection, periodic acid–Schiff reagent (Solarbio, China, Catalog: G1281‐50) was used to stain glycogen according to the manufacturer's instructions. To detect liver fibrosis, Masson's trichrome reagent (Solarbio, China, Catalog: G1340) was used to stain the fibrotic area, according to the manufacturer's instructions.

### Establishment of Gut‐Liver‐on‐a‐Chip Using Cell Lines or Organoids—TEM

Hep‐Orgs on the chips were collected and fixed with pre‐cooled 2.5% glutaraldehyde. The samples were then washed with PBS. To further remove the matrix components, the suspensions were centrifuged at 1500 rpm for 5 min, and this process was repeated three times. The samples were then sliced into ultrathin sections. The images were obtained using TEM (Hitachi, Tokyo, Japan).

### Isolation and Uptake of EXOs—Isolation and Characterization of EXOs

Caco‐2 cells were cultured in MEM complete medium. Once the cell density reached 70–85%, the cells were washed twice with PBS. Then, the cells were cultured in serum‐free MEM supplemented with 50 ng mL^−1^ EGF (R&D, USA, Catalog: P01133), 100 ng mL^−1^ FGF7 (R&D, USA, Catalog: P21781) and 1× insulin transferrin selenium (ITS‐G, iCell, China, Catalog: iCell‐4507) for 24 h. In the case of organoids, Int‐Orgs were differentiated for 7 days. Afterward, the supernatants were collected and stored at ‐80 °C. Subsequently, the thawed supernatants were filtered using a 0.22 µm filter. Ultrafiltration and size exclusion chromatography (SEC) were used to extract the EXOs. The initial 500–1000 mL of the supernatant was concentrated to 2–4 mL through ultrafiltration using an ultrafiltration tube (100 kD, Millipore, USA, Catalog: UFC910096) under centrifugation at 5000 × g. The EXOs were isolated from the concentrated liquid using an exosome purification kit (Exosupur, ECHO EIOTECH, China, Catalog: Echo9101), following the manufacturer's instructions. The protein concentrations of the extracted EXOs were measured using a bicinchoninic acid assay. To identify EXOs from Caco‐2 cells, the EXO‐positive markers TSG101, HSP70, and CD63, and the negative marker calnexin were detected by western blotting. The EXOs derived from Int‐Orgs were characterized for the presence of CD63, CD81, and CD9 markers using an ExoView chip (NanoView Biosciences, USA). Particle size and concentration of the EXOs were analyzed using ZetaView (Particle Metrix, Germany). The EXO morphology was observed using transmission electron microscopy (TEM, HITACHI, Japan).

### Isolation and Uptake of EXOs—Western Blot Analysis

Determine the exosome protein concentration using the Bicinchoninic Acid Protein Assay kit (Beyotime, China, Catalog: P0010). 5×SDS buffer in proportion was added to the quantified exosome concentration (10–30ug) and vortexed and mixed thoroughly. Denaturation in a 95 °C water bath for 5 min. After electrophoresis, the separated gel was removed, and the proteins in the target area within the gel were transferred to a PVDF membrane (Millipore, USA, Catalog: 03010040001). After blocking with 3% BSA blocking buffer, the membranes were incubated with primary and secondary antibodies and exposed to a chemiluminescence developer (Bio‐Rad, USA). Gray analysis of the bands was performed using the ImageJ software. Information on the antibodies used was shown in Table S6 (Supporting Information).

### Isolation and Uptake of EXOs—EXOs Uptake and Inhibition Assay

The isolated EXOs were incubated with PKH26 Red Fluorescent dye (Red, Umibio, China, Catalog: UR52302) for 10 min at RT. In order to trace the uptake of EXOs by HPCs in the chip, culture medium containing 5 µg mL^−1^ (Caco‐2) or 10 µg mL^−1^ (Int‐Orgs) EXOs was prepared and continuously injected into the chip. The uptake of EXOs by HepaRG cells on the chip was observed using a PKH26 tracer (Red, Umibio, China) following a 48 h infusion of EXOs. The internalization of EXOs by Hep‐Orgs on the chip was assessed by examining histological sections obtained from the chip.

For the EXOs inhibition assay, the concentration of GW4869 (MCE, USA, CAS No: 6823‐69‐4) was determined by the expression of EXOs in intestinal epithelial cells and cell growth curves. The intestinal cells were first treated with GW4869 (Caco‐2‐10 µµ, Int‐Orgs‐20 µµ) for 2 h and then cocultured with HPCs on a chip for 6 days. The supernatant was collected, and the expression levels of secreted ALB and AAT were detected by ELISA.

### Animal Experiments

All animal experiments were performed after institutional review by the Animal Ethics Committee of the Zhujiang Hospital, Southern Medical University (approval number: LAEC‐2023‐136). The mice were divided into six groups: a 6‐week control group, a 6‐week CCL4‐induced liver fibrosis group with PBS treatment, a 6‐week CCL4‐induced liver fibrosis group with EXOs treatment, a 9‐week control group, a 9‐week CCL4‐induced liver fibrosis group with PBS treatment, and a 9‐week CCL4‐induced liver fibrosis group with EXOs treatment. As illustrated in Figure 5d, to construct a model of hepatic fibrosis, 4‐week‐old C57BL/6J mice were intraperitoneally (i.p.) injected with carbon tetrachloride (CCL4, AR>99.5%, 5 mL kg^−1^ body weight, diluted at a ratio of 1:5 in olive oil) (Sigma, USA, Catalog:488488) or olive oil alone twice a week for a period–6‐9 weeks. The mice were intravenously injected (i.v.) with EXOs (40ug/time, dissolved in PBS) of Int‐Orgs (differentiation) or PBS solution once a week starting at week 4 until the mice were sacrificed (6 or 9 weeks). The mice were anesthetized with an inhalation anesthetic (isoflurane, 4–5%), followed by orbital blood collection, and the organs were dissected for subsequent experiments.

The distribution of the EXOs (labeled with DIR, Umibio, China, Catalog: UR21017) of Int‐Orgs in mice over a 1‐week period was analyzed using an in vivo imaging system (IVIS Spectrum with excitation/emission wavelengths of 745 nm/800 nm).

### Experiments of EXO miRNAs—EXO miRNAs Sequence and Analysis

Total miRNA from Caco‐2 EXOs was extracted for miRNA sequencing. The Illumina HiSeq2500 platform was used for sequencing, and an miRNA sequencing library was constructed. Read counts were used to detect the miRNA expression levels. All miRNA sequencing data were accessible through SRA PRJNA1145173 in the NCBI database.

### Experiments of EXO miRNAs—Transient Transfection with miRNA Mimics, Inhibitors, and siRNAs

HepaRG cells were transfected with miRNA (miR‐371a‐5p, miR‐372‐3p, and miR‐373‐3p) mimics, inhibitors, negative controls, inhibitor negative controls (NC, iNC), and *RPS6KA2* siRNA oligonucleotides (Tsingke, China) using Lipofectamine 3000 reagent (Invitrogen, USA, Catalog: L3000008). According to the manufacturer's instructions, Caco‐2 cells were transfected with miRNA (miR‐371a‐5p, miR‐372‐3p, and miR‐373‐3p) inhibitors, NC, and iNC, and the sequences used are shown in Tables S3 and S4 (Supporting Information).

### Experiments of EXO miRNAs—Dual Luciferase Reporter Gene Assay

Luciferase reporters were constructed using the pSI‐Check2 plasmid containing the SV40 promoter. The firefly luciferase gene (fLUC) was used as the reporter gene, and the Renilla luciferase gene (hRluc) was used as the internal control. Blank plasmids h‐*RPS6KA2*‐3UTR‐wt, h‐*RPS6KA2*‐3UTR‐mut‐1, and h‐*RPS6KA2*‐3UTR‐mut‐2, were constructed by Biotechnology Co., Ltd. (ReGene, China). Briefly, the HepaRG cells were seeded in 24‐well plates. Blank plasmids (0.5 µg), h‐*RPS6KA2*‐3UTR‐wt, h‐*RPS6KA2*‐3UTR‐mut‐1, or h‐*RPS6KA2*‐3UTR‐mut‐2 were added to the wells. Next, 100 nm miR‐371a‐5p, miR‐372‐3p, or miR‐373‐3p mimics were added. Lipofectamine 3000 reagent was used for transfection. The culture medium was changed 8 h after transfection. After 24 h, supernatants were harvested. Then, 100 µL of fLUC reaction solution and 100 µL of hRluc reaction solution (Yeasen, China, Catalog: 11402ES80) were added to the supernatant, and detection was completed within 30 min. The fLUC/hRluc luminous intensity ratio was used for analysis.

### Experiments of EXO miRNAs—Construction of Plasmids of sgRNA‐dCas9 and Lentiviral Transduction

The synergistic activation mediator (SAM) plasmid consisted of three components: Human RPS6KA2 sgRNA in pGWLV11‐new‐sgRNA‐1/‐2 (MS2) _puro, dCas9‐VP64_Blast, and MS2‐P65‐HSF1_Hygro. Construction of sgRNA plasmids, lentivirus packaging, and quality control were performed by Azenta Life Sciences Co., LTD, USA. HepaRG cells were cultured in Williams’ E medium (Gibco, USA, Catalog: A1217601) supplemented with HepaRG general‐purpose medium (Thermo Fisher, USA, Catalog: HPRG670), 1×GlutaMAX (Thermo Fisher, USA, Catalog: 35050061), 100 U mL^−1^ penicillin, and 0.1 mg mL^−1^ streptomycin. Cells were passaged every 5 days at a ratio of 1:3.5 × 105 cells were seeded in 6‐well plates and incubated with LV‐Assistant, dCas9‐VP64, and MS2‐P65‐HSF1 lentiviral supernatant (MOI = 10) for 24 h. Transfected cells were replated at low density (0.8 × 10^5^ per well for a 24‐well plate), and a selection agent was added 6 h after plating (10 ug mL blasticidin and 200 ug mL^−1^ hygromycin, Sigma: SBR00022, 400051). Cells were transduced with RPS6KA2 sgRNAs lentiviral vectors (MOI = 10) for 24 h. 10 ug mL^−1^ puromycin was added to the media for selection, with the selection period lasting for 5 days. The sequences of RPS6KA2 sgRNAs are listed in Table S4 (Supporting Information).

### Statistical Analysis

The results were presented as the average ± standard error, and all statistical analyses were carried out using GraphPad Prism 9.0. Data between the two groups were compared using an unpaired two‐tailed Student's *t*‐test. One‐way ANOVA was used to compare multiple groups of sample data with a single variable, and two‐way ANOVA was used for multigroup comparisons with two independent variables, followed by a Tukey HSD post hoc test. A threshold of p<0.05 was used to define significant differences. All data were obtained from at least three independent experiments.

## Conflict of Interest

The authors declare no conflict of interest.

## Supporting information



Supporting Information

## Data Availability

The data that support the findings of this study are available from the corresponding author upon reasonable request.
