# Supplementary material for: Exosomes From Intestinal Epithelial Cells Promote Hepatic Differentiation of Liver Progenitor Cells in Gut‐Liver‐on‐a‐Chip Models
Source: Adv Sci (Weinh). 2025 Jul 6;12(32):e17478. doi: 10.1002/advs.202417478 (PMC12407332; doi:10.1002/advs.202417478)
Supplement: Supplementary file 1 — Supporting Information [file ADVS-12-e17478-s001.docx]

Supporting Information

**Exosomes from intestinal epithelial cells promote hepatic differentiation of liver progenitor cells in gut-liver-on-a-chip models**

*Liang Ye^1^, Shi Li^1,2^, Guofang Bi^3^, Binghui Li,^1,2^ Zhai Cai^4^, Meixian Jin^5^, Ying Zhang^2^, Wanren Yang^1,2^, Yang Li^1^, Shao Li^1^, Wei Hu^6^, Yi Gao^1^, Mingxin Pan^1^, Shuqin Zhou^5, 6 *^, Chao Zhang^7*^, Huichang Bi^3 *^, Qing Peng ^2, 8 *^*

Supporting Information figures

**
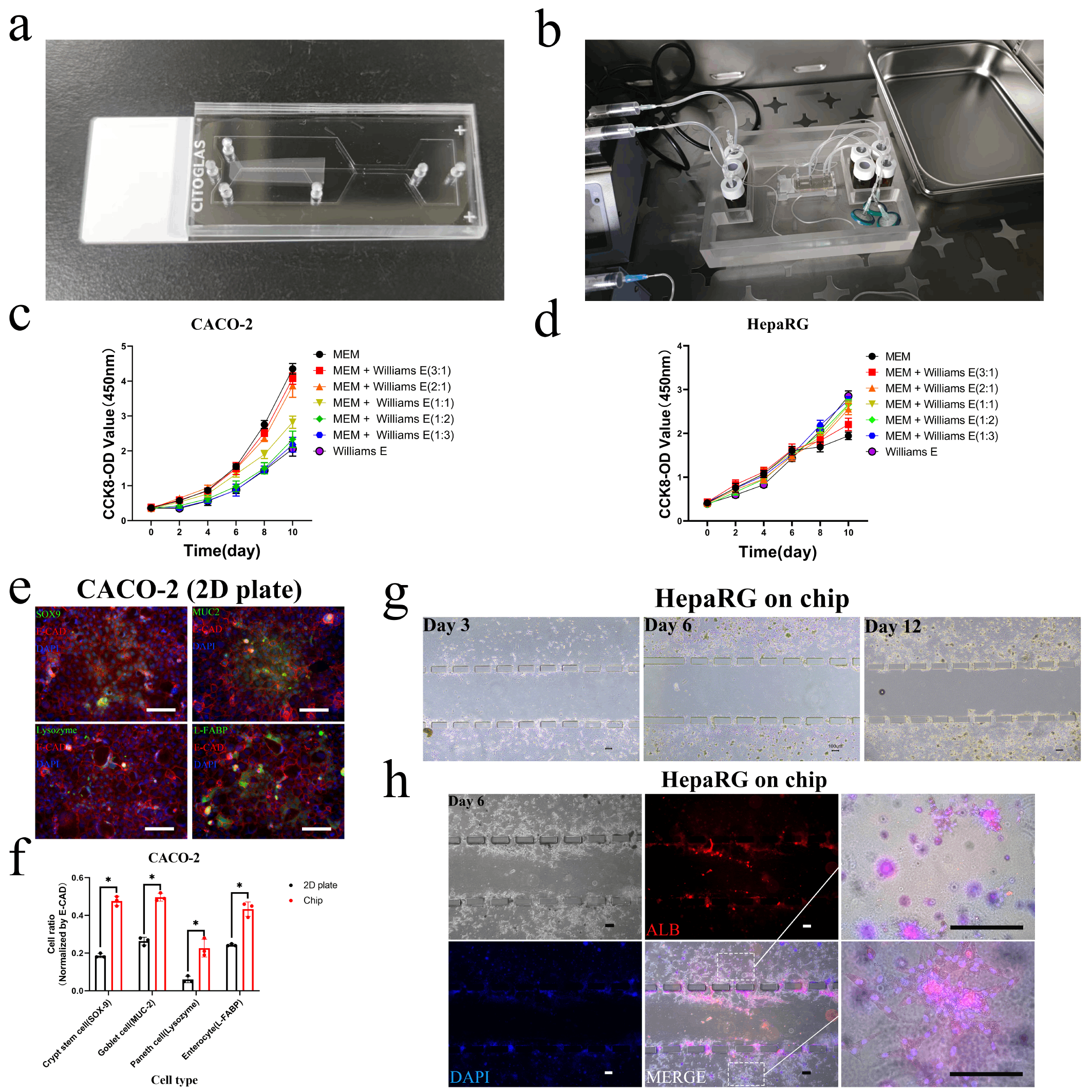
**

**Figure S1. Construction of gut-liver-on-a-chip.**

(a) The physical picture of a gut-liver-on-a-chip.

(b) Photograph of the assembled microfluidic device.

(c-d) The growth curves of Caco-2 (c) and HepaRG (d) cells measured by CCK8 in different proportions of MEM and Williams’ E complete medium (n = 3).

(e) Caco-2 cells were cultured in a 24-well plate, and immunostaining was conducted to determine the localization of distinct intestinal cells, including SOX9+ stem cells (green), MUC2+ goblet cells (green), lysozyme+ Paneth cells (green) or L*-*FABP+ enterocytes (green). Scale bar =100 μm.

(f) Relative proportion of characteristic intestinal cells cultured in a chip and a plate, as determined by immunofluorescence colocalization staining (*n* = 3, mean ± SD. Student’s t-tests). **p*<0.05.

(g) Bright-field images of HepaRG cells that were cultured for 3, 6, and 12 days in the liver chamber. Scale bar =100 μm.

(h) HepaRG cells cultured on the chip for 6 days were stained with antibodies against ALB (red) and DAPI (blue). Scale bar=100 μm.


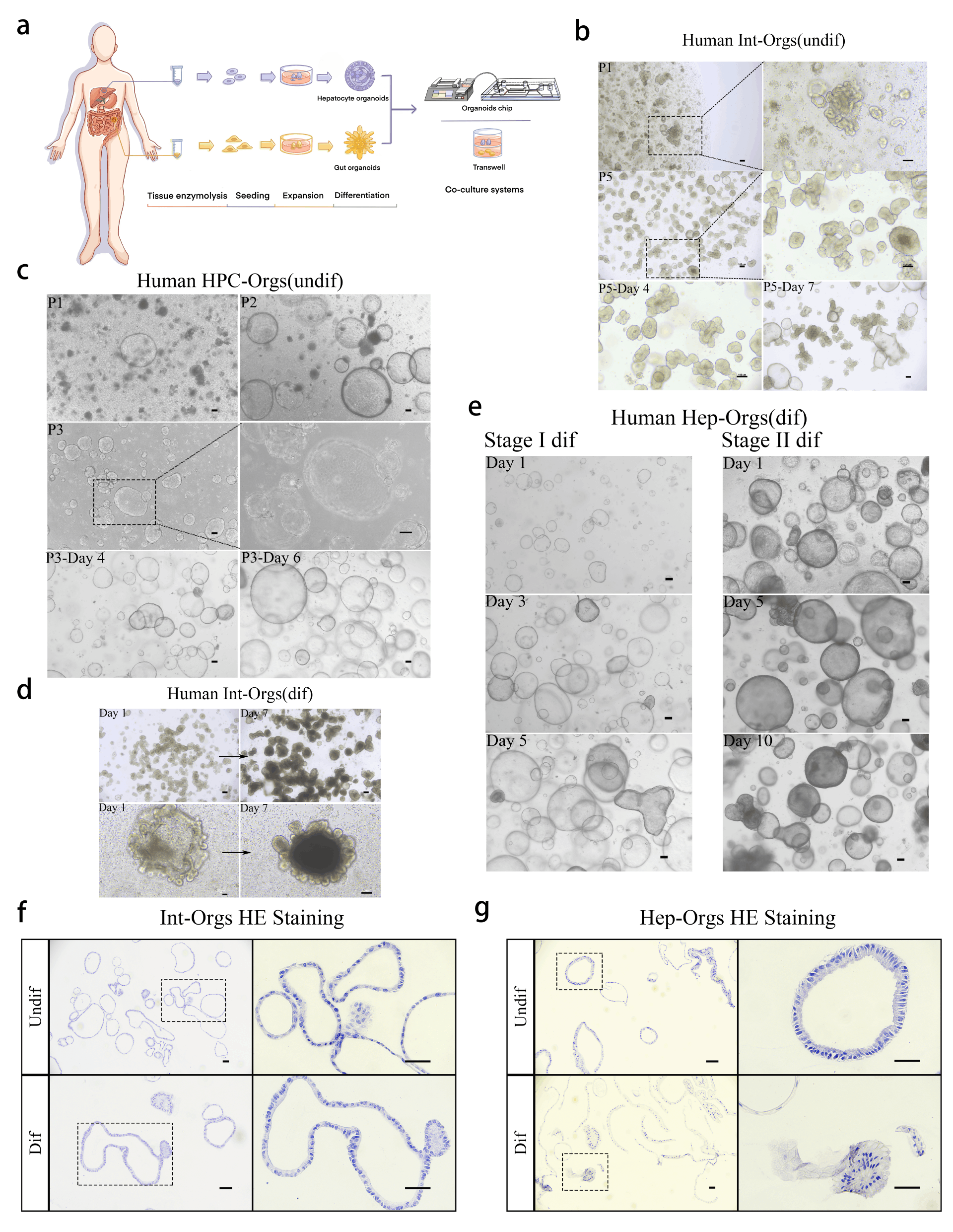


**Figure S2. Construction and differentiation of human Int-Orgs and HPC-Orgs**

(a) Schematic of experimental procedure for organoids acquisition and culture.

(b) The bright field images of Int-Orgs. Upper left and upper right panels: low magnification field and high magnification field of Int-Orgs in Passage 1; Middle left and middle right panels: Low magnification field and high magnification field of Int-Orgs in Passage 5; Lower left and lower right panels: General visual fields of Int-Orgs in Passage 5 cultured continuously for 4 days and 7 days. Scale bar= 100 μm.

(c) The bright field images of HPC-Orgs. Upper left panel: extracted liver tissue cells in Passage 1; upper right panel: HPC-Orgs in Passage 2; middle left and middle right panels: low magnification field and high magnification field of HPC-Orgs in Passage 3; Lower left and lower right panels: HPC-Orgs in Passage 3 continuous culture for 4 days and 6 days. Scale bar= 100 μm.

(d) The bright field images of Int-Orgs differentiation. Upper left and upper right panels: Int-Orgs after differentiation for 1 day and 7 days (low magnification field); Lower left and lower right panels: Int-Orgs after differentiation for 1 day and 7 days (high magnification field). Scale bar= 100 μm.

(e) The bright field images of HPC-Orgs differentiation. Upper left panel, middle left panel and lower left panel: 1 day, 3 days, and 5 days of differentiated HPC-Orgs in the first stage; upper right panel, middle right panel and lower right panel: 1 day, 5 days, 10 days of differentiated HPC-Orgs in the second stage. Scale bar= 100 μm.

(f-g) The HE fields images of undifferentiated (top) and differentiated (bottom) Int-Orgs (f) and Hep-Orgs (g), low magnification field (left) and high magnification field (right). Scale bar= 100 μm.

**
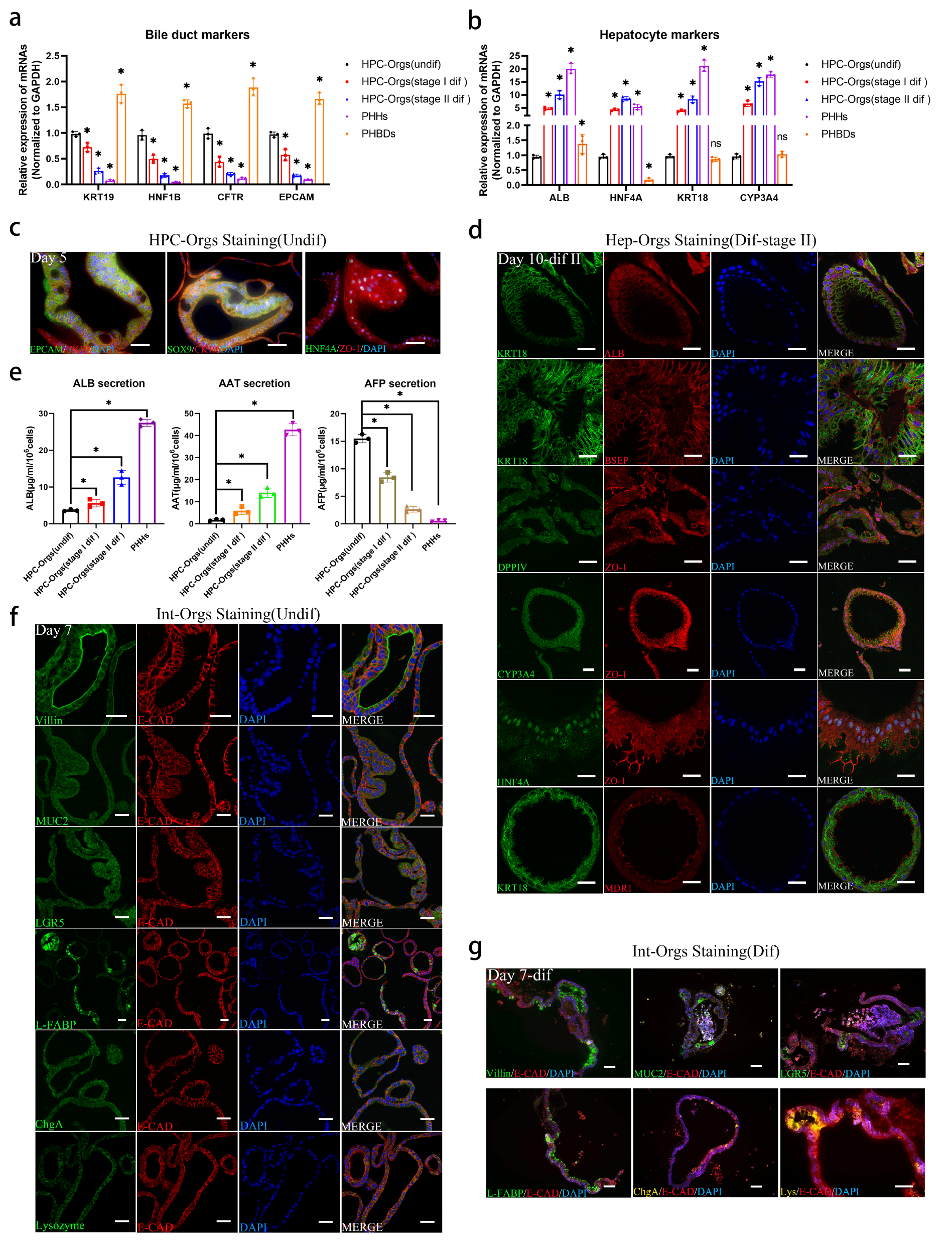
Figure S3. Identification of the differentiated features of human Int-Orgs and Hep-Orgs.**

(a) Expression of gene related with bile duct markers (*KRT19*, *HNF1β*, *CFTR*, *EPCAM*) in HPC-Orgs (undif), HPC-Orgs (stage I dif), HPC-Orgs (stage II dif), PHHs and PHBOs groups was detected by qRT-PCR (*n* = 3, mean ±SD, One-way ANOVA). **p*<0.05. PHHs: Primary human hepatocytes; PHBDs: Primary human intrahepatic bile duct cells.

(b) Expression of gene related with hepatocyte markers (*ALB*, *HNF4A*, *KRT18*, *CYP3A4*) in HPC-Orgs (undif), HPC-Orgs (stage I dif), HPC-Orgs (stage II dif), PHHs and PHBOs groups were detected by qRT-PCR (*n* = 3, mean ±SD. One-way ANOVA). **p*<0.05.

(c) Immunofluorescence staining of paraffin sections of undifferentiated HPC-Orgs (day 5), bile duct cells marker CK19+(red)/EPCAM+(green), hepatocytes marker HNF4A+(green), stem cells marker SOX9+(green), ZO*-*1(red), DAPI (blue). Scale bar= 100 μm.

(d) Immunofluorescence staining of paraffin sections of second stage differentiated Hep-Orgs (day 10), hepatic markers KRT18, DPPIV, CYP3A4, HNF4A (green), ALB, BSEP, ZO*-*1 and MDR1 (red). DAPI (blue) shows the nuclei. Scale bar= 100 μm.

(e) ELISA data of the level of secreted ALB, AAT and AFP of HPC-Orgs at the stage of undifferentiation (undif), the first stage of differentiation (stage I dif), the second stage of differentiation (stage II dif) and PHHs respectively (*n* = 3, mean ±SD, One-way ANOVA). **p*<0.05.

(f) Confocal images of paraffin sections of undifferentiated Int-Orgs (day 7), immunofluorescence staining was used to detect various intestinal cells with different markers, including Villin+ papilla cells (green), MUC2+ goblet cells (green), LGR5+ crypt stem cells (green), L*-*FABP+ enterocytes (green), ChgA+ enteroendocrine cells (green), Lysozyme+ Paneth cells (green) and E*-*CAD (red). DAPI (blue) shows the nuclei. Scale bar= 100 μm.

(g) Fluorescent images of paraffin sections of differentiated Int-Orgs (day 7), immunostaining was used to detect the markers of various intestinal cells, including Villin, MUC2, LGR5, L*-*FABP (green), ChgA, Lysozyme (yellow), E*-*CAD (red). DAPI (blue) shows the nuclei. Scale bar= 100 μm.


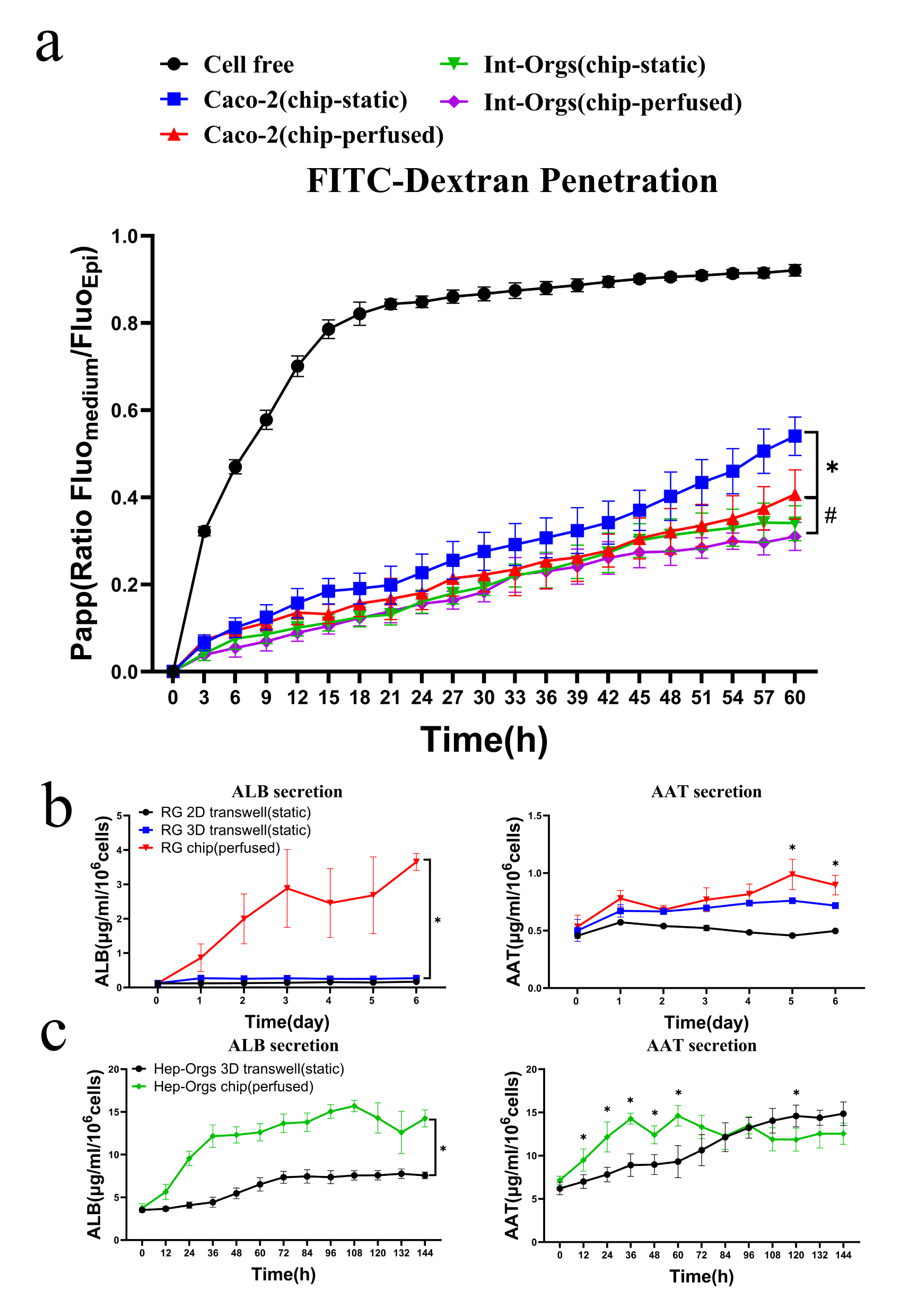


**Figure S4. Comparison of chips and static system.**

(a) Integrity and permeability of Int-Orgs and Caco-2 on chips detected by measuring FITC-dextran perfusion (*n* = 3, mean±SD, Two-way ANOVA).**p*<0.05 for Caco-2(chip-static) vs Caco-2(chip-perfused); #*p*<0.05 for Caco-2(chip-perfused) vs Int-Orgs(chip-perfused).

(b) ELISA data of the levels of secreted ALB and AAT in HepaRG cells monocultured on 2/3D transwell and chip systems within 6 days (n = 3, mean±SD, Two-way ANOVA).**p*<0.05 for RG chip(perfused) vs RG 3D transwell (static).

(c) ELISA data of the level of secreted ALB and AAT in Hep-Orgs monocultured on 3D transwell and chip systems during 6 days of stage of Differentiation II (*n* = 3, mean±SD, Two-way ANOVA). **p*<0.05.


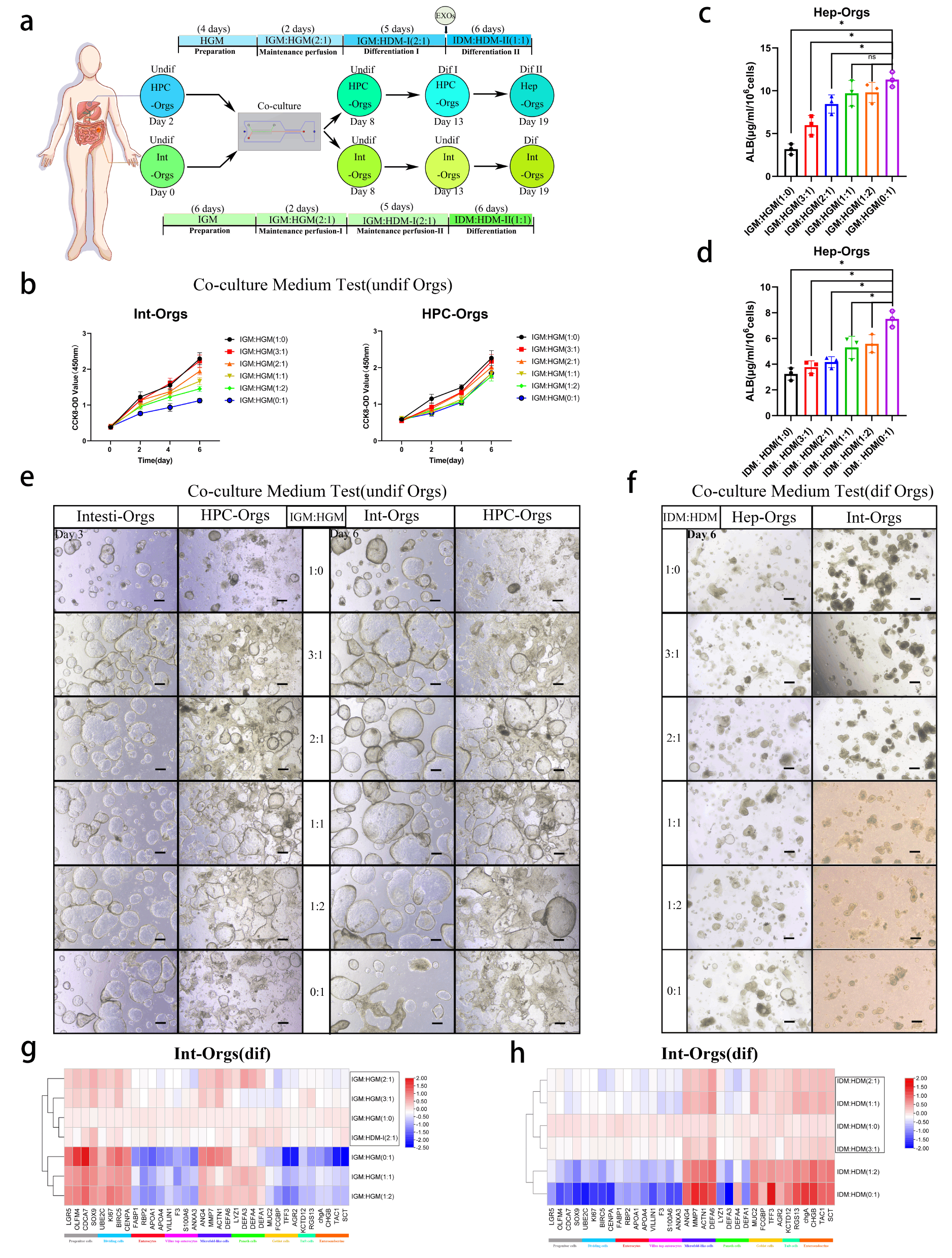


**Figure S5. Determination of organoids coculture medium**

(a) A schema of the experimental procedure for organoid coculture and differentiation on the chip.

(b) The growth curves of Int-Orgs cells (left) and HPC-Orgs cells (right) measured by CCK8 under the condition of mixed IGM and HGM medium in different proportions (*n* = 3).

(c) ELISA data of the levels of secreted ALB in Hep-Orgs cultured with different proportions of IGM and HGM medium after 10 days of differentiation (*n* = 3, mean ±SD, One-way ANOVA). **p*<0.05.

(d) The expression levels of secreted ALB in Hep-Orgs cultured with different proportions of IDM and HDM medium detected by ELISA (*n* = 3, mean ±SD, One-way ANOVA). **p*<0.05.

(e) The bright field images of organoids cultured with mixed IGM and HGM expansion medium in different proportions. Columns presented from the left to the right: undifferentiated Int-Orgs cultured for 3 days, HPC-Orgs cultured for 3 days, undifferentiated Int-Orgs cultured for 6 days and HPC-Orgs cultured for 6 days. Scale bar= 100 μm.

(f) The bright field images of Hep-Orgs (left) and Int-Orgs (right) cultured for 6 days with different proportions of IDM and HDM differentiation medium. Scale bar= 100 μm.

(g) Expression of specific genes of intestinal cells in Int-Orgs cultured with different proportions of IGM and HGM (including HDM-I) medium detected by qRT-PCR after complete differentiation. The heat map displays the log2 (fold change) value of the genes. Correlation analysis was conducted according to the gene expression trend of each group (*n* = 3). The intestinal cell types in the figure include Progenitor cells, Dividing cells, Enterocytes, Villus top enterocytes, Microfold-like cells, Enteroendocrine, Tuft cells, Goblet cells, and Paneth cells.

(h) Expression of specific genes of intestinal cells in Int-Orgs cultured with different proportions of IDM and HDM (HDM-II) medium were detected by qRT-PCR. The heat map displays the log2 (fold change) value of the genes. Correlation analysis was conducted according to the gene expression trend of each group. The intestinal cell types included in the figure are the same as those in panel f.


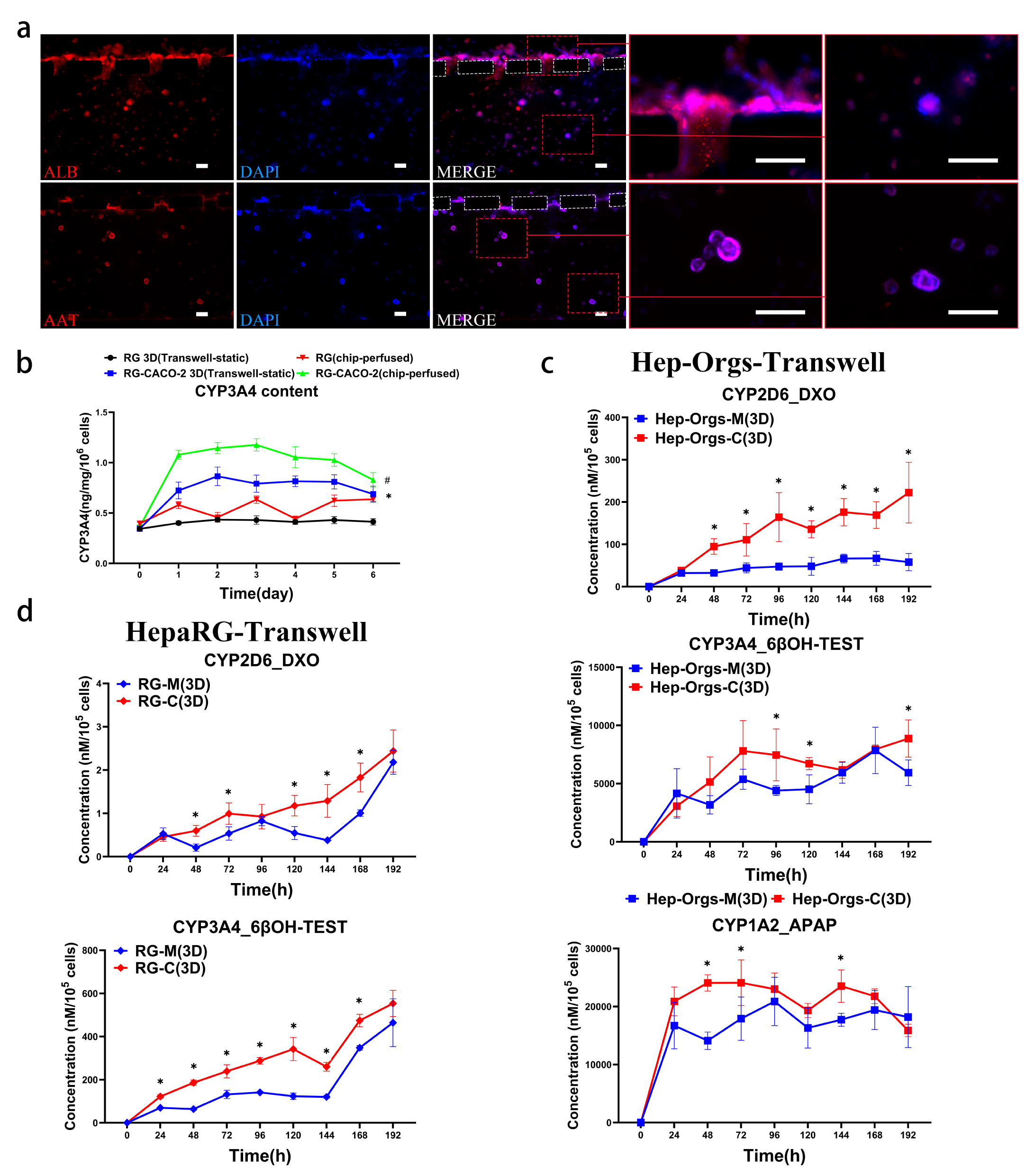


**Figure S6. The effects of cocultured Int-Orgs on hepatic differentiation of HPC-Orgs on Transwell and the regional effects of cocultured Caco-2 cells on hepatic differentiation of HepaRG cells on chip.**

(a) Caco-2 and HepaRG cells cocultured on the chip for 5 days were stained with antibodies against ALB (red), AAT (red) and DAPI (blue). The two fluorescence images on the right are the high-magnification images of HepaRG cells near and far from the central channel of the hepatic sinusoid, respectively. Scale bar=100 μm.

(b) ELISA data of the levels of CYP3A4 content in HepaRG cells monocultured and cocultured in the Transwell system and chip within 6 days (*n* = 3，mean ± SD, Two-way ANOVA). **p*<0.05 for RG-CACO-2 3D(Transwell-static) vs RG 3D(Transwell-static); #*p*<0.05 for RG-CACO-2 3D(chip-perfused) vs RG 3D(Transwell-static).

(c) Measurement of CYP metabolites: DXO (CYP2D6), 6βOH-TEST (CYP3A4) and APAP (CYP1A2) in Hep-Orgs mono- and coculture groups in Transwell device within 8 days during stage of Differentiation II(*n* = 3，mean ± SD, Two-way ANOVA), **p*<0.05.

(d) The concentrations of CYP metabolites: DXO (CYP2D6) and 6βOH-TEST (CYP3A4) in HepaRG cell mono- and coculture groups in a Transwell device within 8 days (*n* = 3，mean ± SD, Two-way ANOVA). **p*<0.05.


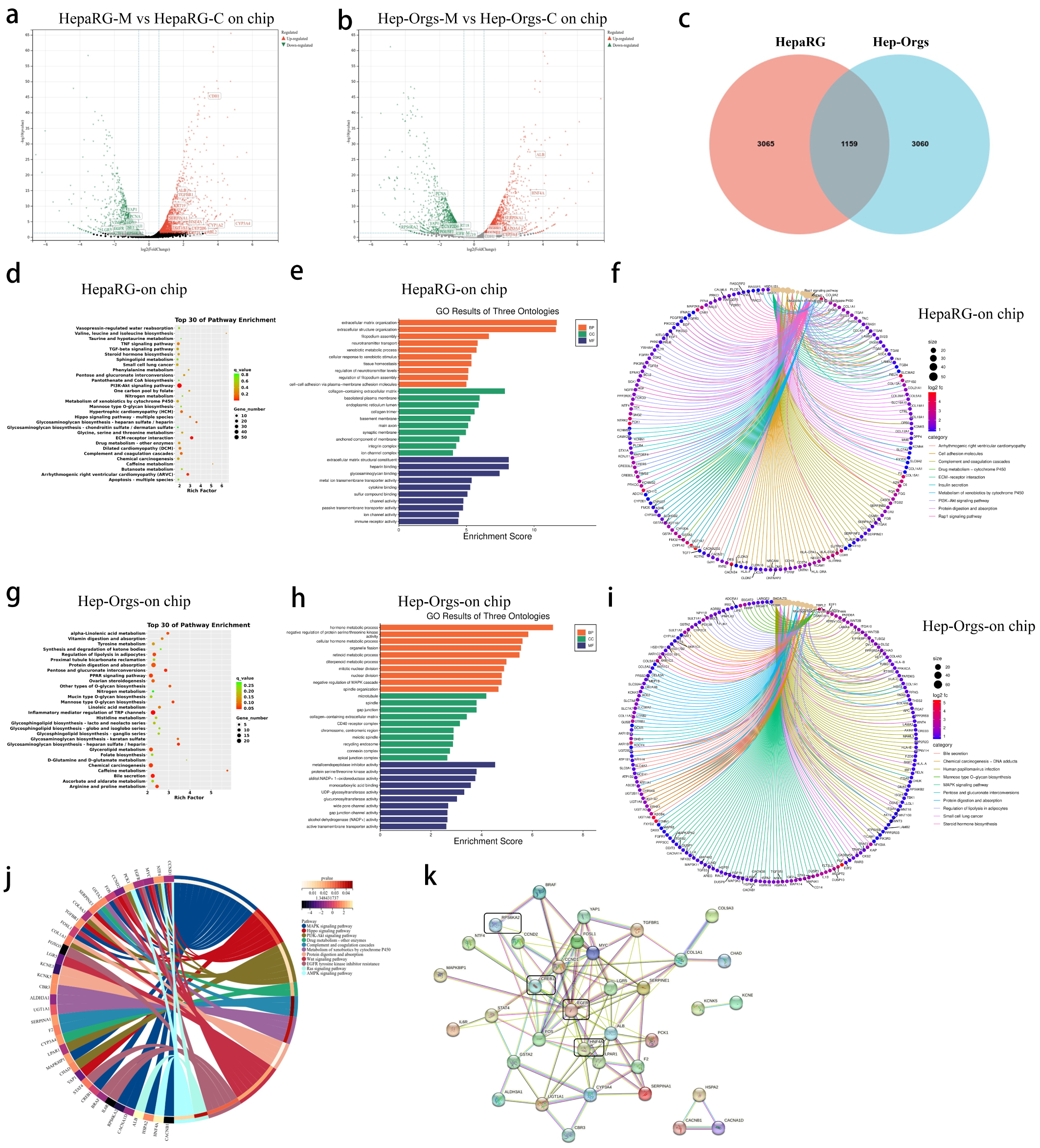


**Figure S7. Transcriptome sequencing analysis of mono- and cocultured HepaRG and Hep-Orgs on chip**

(a-b) Volcanic maps show the distribution of DEGs in mono- and cocultured HepaRG (a) and Hep-Orgs (b) on the chip. Green dots represent downregulated genes, and red dots represent the upregulated genes in the coculture systems.

(c) The Venn diagram shows the intersection of DEGs in HepaRG and Hep-Orgs on chip.

(d) The bubble maps show the KEGG enrichment analysis results of HepaRG on chip. The top 30 enrichment pathways were listed.

(e) The bar charts show the GO enrichment analysis results of HepaRG on chip. Enrichment included three parts: BP (biological process), CC (cell components) and MF (molecular function).

(f) Correlation circle diagrams showing the Enrichment pathways of DEGs in HepaRG.

(g) The bubble maps show the KEGG enrichment analysis results of and Hep-Orgs on chip. The top 30 enrichment pathways were listed.

(h) The bar charts show the GO enrichment analysis results of Hep-Orgs on chip. Enrichment included three parts: BP (biological process), CC (cell components) and MF (molecular function).

(i) Correlation circle diagrams showing the Enrichment pathways of DEGs in Hep-Orgs on chip.

(j) Chord diagram showing the related pathways of DEGs with the same trend in two sets of chips (HepaRG and Hep-Orgs).

(k) PPI protein interaction network shows protein interactions generated by DEGs.


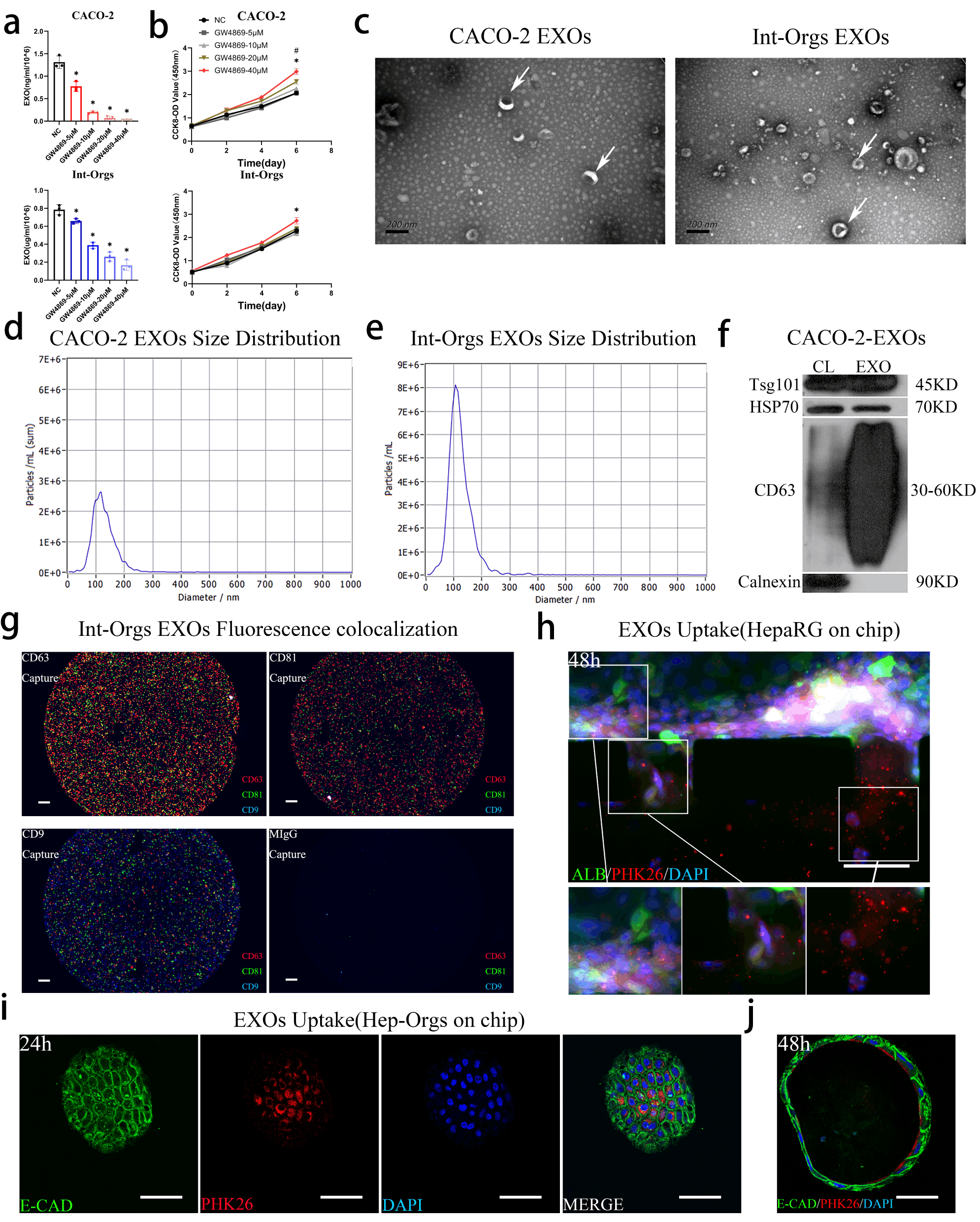


**Figure S8. Identification of EXOs of Caco-2 and Int-Orgs**

(a) The relative EXO concentrations harvested from the Caco-2 cells and Int-Orgs treated with GW4869 at different concentrations (5 μM, 10 μM, 20 μM, 40 μM) (*n* = 3，mean ± SD, One-way ANOVA). **p*<0.05, compared with NC.

(b) Growth curves of Caco-2 cells and Int-Orgs treated with different concentrations of GW4869 (*n* = 3，mean ± SD, Two-way ANOVA).Upper panel: **p*<0.05 for GW4869-20uM vs NC (negative control), #*p*<0.05 for GW4869-40uM vs NC; Lower panel: **p*<0.05 for GW4869-40uM vs NC.

(c) TEM images of EXOs extracted from Caco-2 and Int-Orgs. Scale bar= 200nm.

(d-e) The peak maps show the NTA analysis of EXOs derived from Caco-2 (d) and Int-Orgs (e).

(f) Identification of EXOs from Caco-2 using WB assay. Detection of exosome markers (Tsg101, HSP70 and CD63) and negative control (Calnexin) in EXOs extracted from Caco-2 cells supernatant and cell lysate (CL) (*n* = 3).

(g) Single particle interferometric reflectance imaging sensing analysis (SP‐IRIS) was performed. Fluorescent antibodies against CD81 (green), CD63 (red)and CD9 (blue) were used to detect the positive markers. Anti-Mouse IgG antibody was set as the negative control. Scale bar= 10 μm.

(h) The tracer PKH26 (red) marks EXOs uptake in HepaRG cells in the hepatic sinuses area on the chip after 48hrs, hepatocyte fluorescence localization ALB (green) and DAPI (blue). Left panel and right panel: Low magnification field and EXOs detail maps. Scale bar= 100 μm.

(i) EXOs of Int-Orgs labeled with tracer PKH26 (red) were ingested into Hep-Orgs in the hepatic sinuses area on the chip after 24hrs. Hep-Orgs cells contour immunostaining was labeled with E*-*CAD (green) and DAPI (blue). Scale bar= 100 μm.

(j) Fluorescence profile of EXOs ingested by Hep-Orgs after 48hrs. Scale bar= 100 μm.


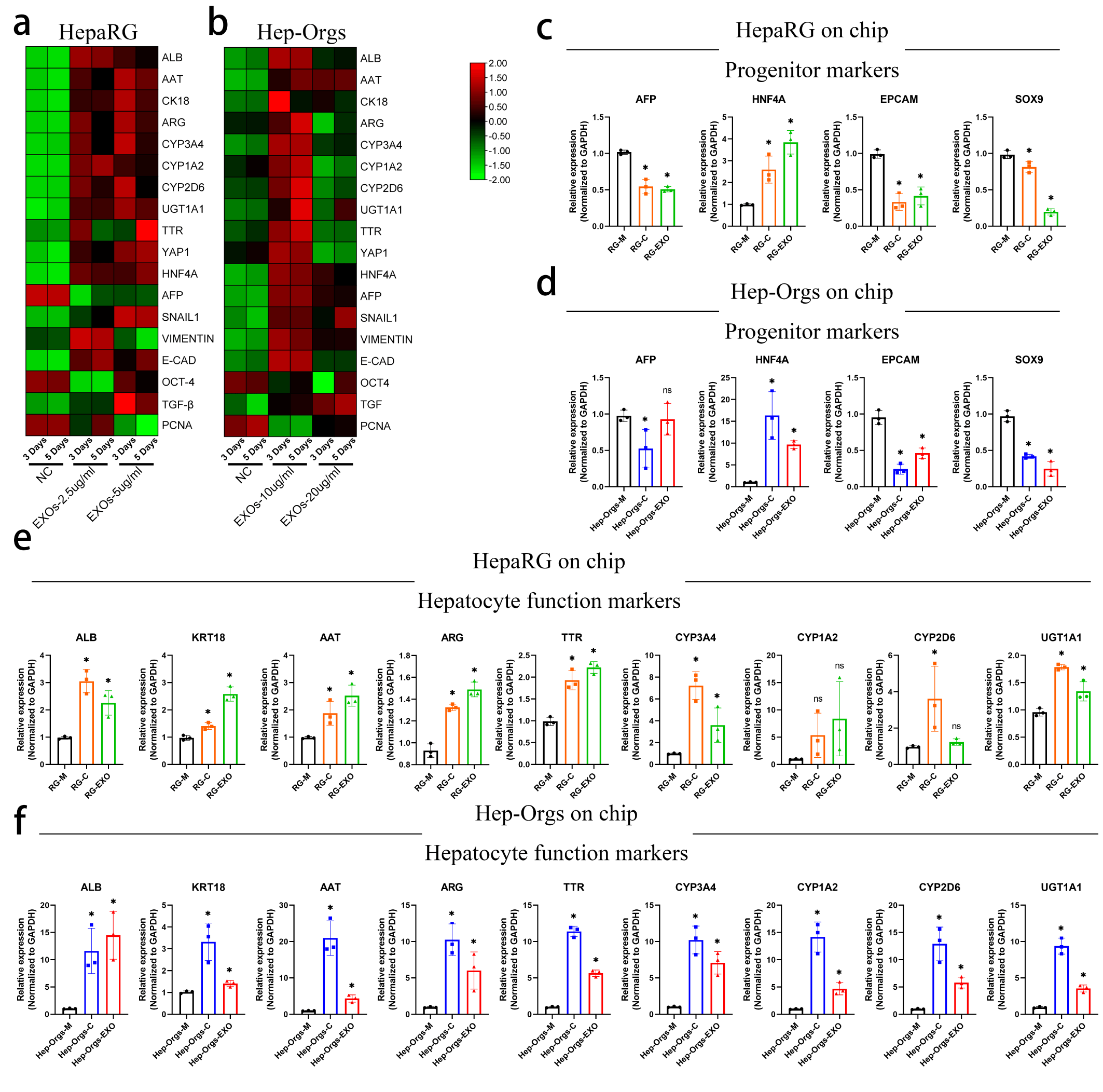


**Figure S9. Effects of EXOs of intestinal epithelial cells on regulating the hepatic differentiation related genes in HPCs**

(a) Comparison of gene expression related to function and stemness in HepaRG cells treated with 2.5μg/ml and 5μg/ml exosomes (derived from Caco-2) on chips detected by qRT-PCR for 3 and 5 days. The heat map displays the log2 (fold change) value of the genes (*n* = 3).

(b) Comparison of gene expression related to function and stemness in Hep-Orgs treated with 10μg/ml and 20μg/ml EXOs on chips detected by qRT-PCR for 3 and 5 days. The heat map displays the log2 (fold change) value of the genes (*n* = 3).

(c-d) Expression differences of genes of hepatic progenitor markers in HepaRG (c) and Hep-Orgs (d) on chips detected by qRT-PCR in monoculture, coculture and EXOs-treated groups (HepaRG-5μg/ml, Hep-Orgs-10μg/ml) (*n* = 3，mean ± SD, One-way ANOVA). **p*<0.05, compared with RG-M or Hep-Orgs-M.

(e-f) Expression differences of genes of hepatic function markers (e) in HepaRG (e) and Hep-Orgs (f) on chips detected by qRT-PCR in monoculture, coculture and EXOs-treated groups (HepaRG-5μg/ml, Hep-Orgs-10μg/ml) (*n* = 3，mean ± SD, One-way ANOVA). **p*<0.05, compared with RG-M or Hep-Orgs-M.


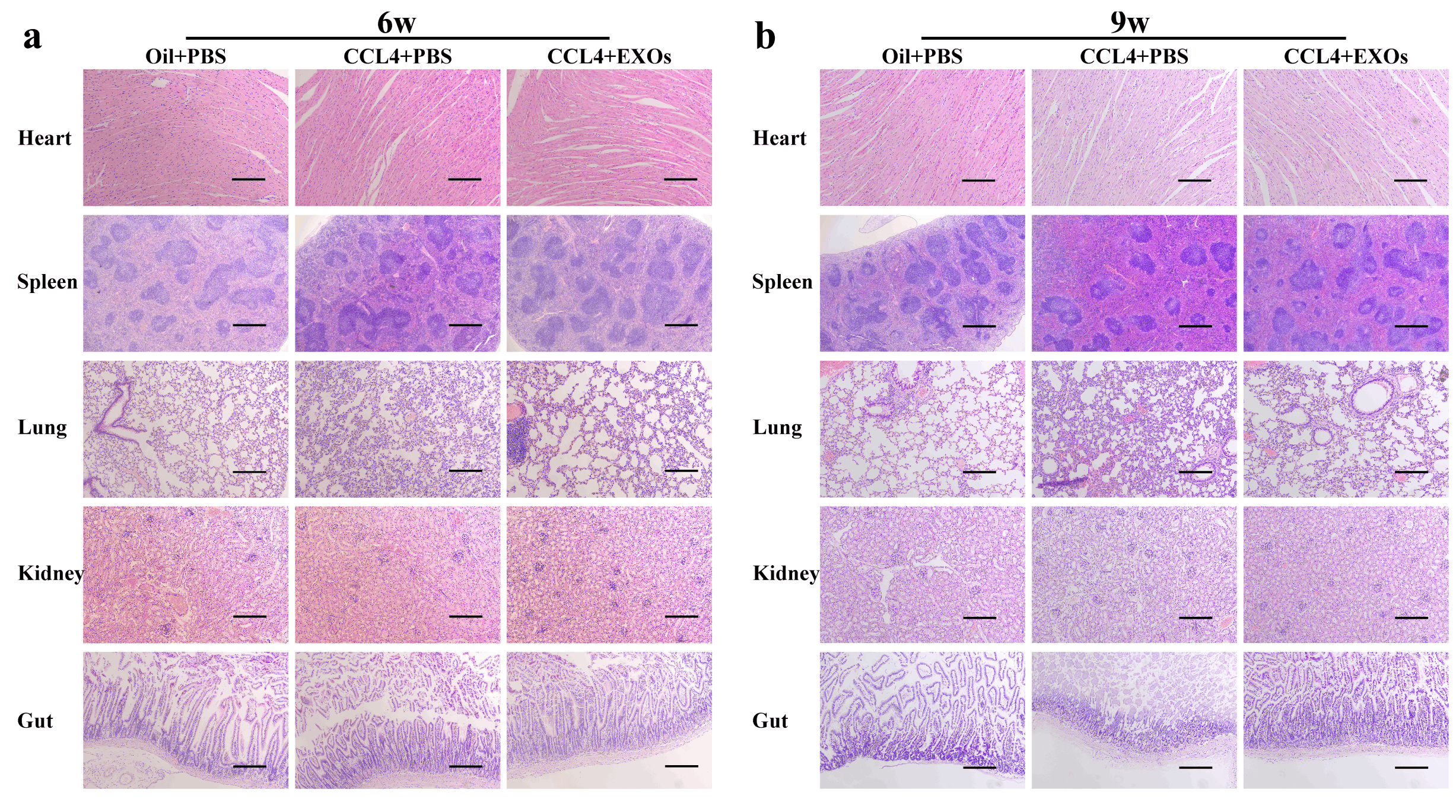


**Figure S10. Treatment of EXOs from Int-Orgs ameliorated the pathologic manifestations of extrahepatic organs in mice with hepatic fibrosis**

(a-b) The HE staining images of heart, spleen, lung, kidney and gut of 6-week (a) and 9-week (b) CCL4-induced

mice in control groups (Oil+PBS), modelling groups (CCL4+PBS) and treatment groups (CCL4+EXOs)（*n* = 6）. Scale bar= 100 μm.

**
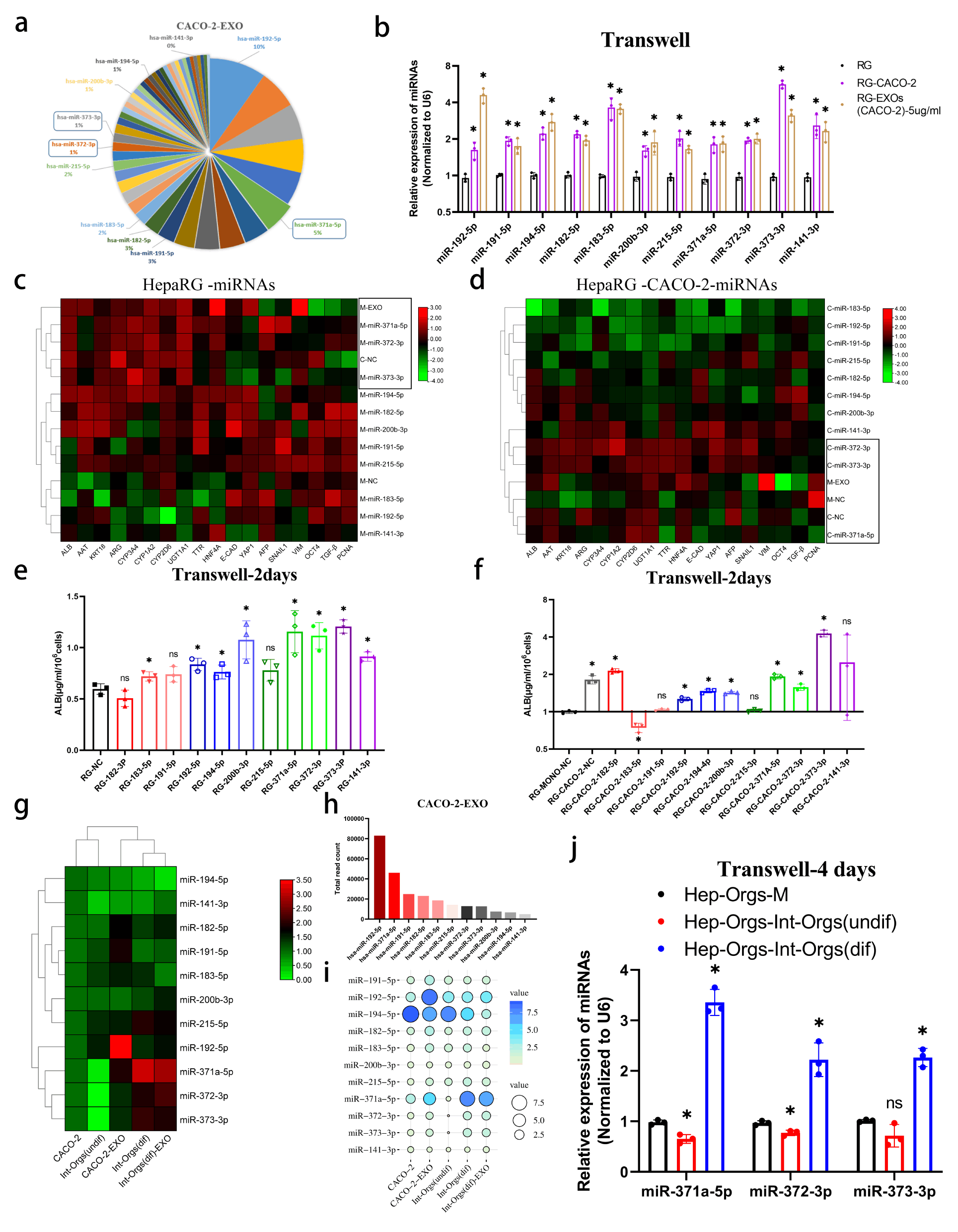
**

**Figure S11. The selection of miRNAs**

(a) The pie chart shows the top 50 highly expressed miRNAs in Caco-2 EXOs according to the miRNAs sequencing data.

(b) Expression of 11 miRNAs in cocultured and EXOs-treated HepaRG cells detected by MiRNA qRT-PCR (*n* = 3，mean ± SD, One-way ANOVA). **p*<0.05, compared with RG.

(c-d) In mono-(c) and coculture(d) conditions, qRT-PCR was used to detect the expression of genes related with hepatic differentiation and stemness in HepaRG cells. M-NC: monocultured HepaRG cells transfected with NC; C-NC: cocultured HepaRG cells transfected with NC; M-EXO: EXOs-treated HepaRG cells tranfected with NC; M-miRNAs: monocultured HepaRG cells transfected with 11 miRNAs mimics. C-miRNAs: cocultured HepaRG cells transfected with 11 miRNAs mimics. The heat map displays the log2 (fold change) value of the genes. Correlation analysis was conducted according to the gene expression trend of each group (*n* = 3).

(e-f) ELISA data of the level of secreted ALB in HepaRG cells (transfected with 11 miRNAs mimics) mono-(e) and coculture (f) in Transwell (2D) devices at day 2 after transfection (*n* = 3，mean ± SD, One-way ANOVA). **p*<0.05, compared with RG-NC or RG-MONO-NC.

(g) Expression of 11 miRNAs in Caco-2 (control), EXOs of Caco-2, undifferentiated Int-Orgs, differentiated Int-Orgs, and EXOs of differentiated Int-Orgs detected by MiRNA qRT-PCR. The heat map displays the log2 (fold change) value of the genes. Correlation analysis was conducted according to miRNAs expression trend of each group (*n* = 3).

(h) The bar chart shows the total read count of 11 miRNAs in Caco-2 EXOs sequencing.

(i) The bubble chart shows the relative content of 11 miRNAs in Caco-2, EXOs of Caco-2, Int-Orgs (undif), Int-Orgs (dif), and EXOs of Int-Orgs (dif).

(j) The chart shows the expression of miR-371-373 cluster in Hep-Orgs cocultured with undifferentiated and differentiated Int-Orgs detected by miRNA qRT-PCR at day 4 after coculture (*n* = 3，mean ± SD, One-way ANOVA). **p*<0.05, compared with Hep-Orgs-M (Monocultured Hep-Orgs).


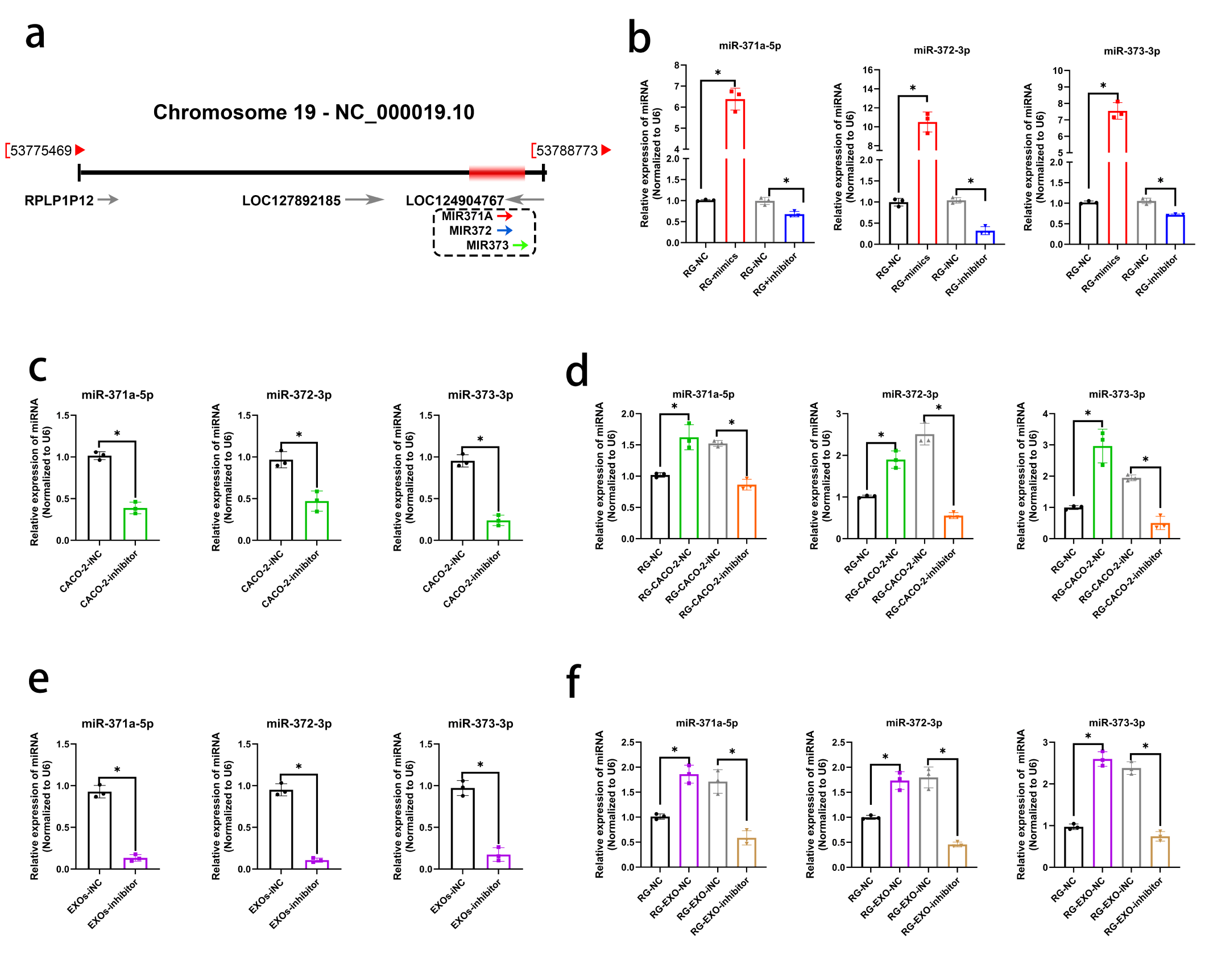


**Figure S12. Validation of transfection efficiency of miR-371-373 cluster mimics and inhibitors**

(a) Transcript region distribution of hsa-miR-371a/372/373 on chromosome 19 (Source from NCBI database).

(b) Transfection efficiency of miR-371-373 cluster mimics and inhibitors in HepaRG cells detected by miRNA qRT-PCR(*n* = 3，mean ± SD, One-way ANOVA). **p*<0.05, compared with RG-NC.

(c) Transfection efficiency of miR-371-373 cluster inhibitors in Caco-2 cells detected by miRNA qRT-PCR (*n* = 3，mean ± SD, Student’s t-tests). **p*<0.05.

(d) Expression of miR-371-373 cluster in HepaRG cells cocultured with Caco-2 cells (transfected with miR-371-373 inhibitors) detected by miRNA qRT-PCR (*n* = 3，mean ± SD, One-way ANOVA). **p*<0.05.

(e) Expression of miR-371-373 cluster in EXOs of Caco-2 cells (transfected with miR-371-373 inhibitors) detected by miRNA qRT-PCR (*n* = 3, mean ± SD, Student’s t-tests). *p<0.05.

(f) Expression of miR-371-373 cluster in HepaRG cells treated with EXOs of Caco-2 (transfected with miR-371-373 inhibitors) detected by miRNA qRT-PCR (*n* = 3, mean ± SD, One-way ANOVA). *p<0.05.

Supporting Information Table

**Table S1. The sequence of mRNAs primers used in this article.**

| **Gene** | **Forward primer (5’-3’)** | **Reverse primer (5’-3’)** |
| --- | --- | --- |
| ***ALB*** | TTTATGCCCCGGAACTCCTTT | AGTCTCTGTTTGGCAGACGAA |
| ***AAT*** | ATGCTGCCCAGAAGACAGATA | CTGAAGGCGAACTCAGCCA |
| ***KRT18*** | GGCATCCAGAACGAGAAGGAG | ATTGTCCACAGTATTTGCGAAGA |
| ***KRT19*** | AACGGCGAGCTAGAGGTGA | GGATGGTCGTGTAGTAGTGGC |
| ***ARG*** | GTTGAACCCCAGGCACTAAAT | CAACGAAGAGATTAGGGTCACTC |
| ***CYP3A4*** | AAGTCGCCTCGAAGATACACA | AAGGAGAGAACACTGCTCGTG |
| ***CYP1A2*** | CTGGGCACTTCGACCCTTAC | TCTCATCGCTACTCTCAGGGA |
| ***CYP2D6*** | CCAACGGTCTCTTGGACAAAG | GGGTCGTCGTACTCGAAGC |
| ***UGT1A1*** | CATGCTGGGAAGATACTGTTGAT | GCCCGAGACTAACAAAAGACTCT |
| ***TTR*** | TGGGAGCCATTTGCCTCTG | AGCCGTGGTGGAATAGGAGTA |
| ***YAP1*** | TAGCCCTGCGTAGCCAGTTA | TCATGCTTAGTCCACTGTCTGT |
| ***HNF4A*** | CACGGGCAAACACTACGGT | TTGACCTTCGAGTGCTGATCC |
| ***HNF1β*** | ACCAAGCCGGTCTTCCATACT | GGTGTGTCATAGTCGTCGCC |
| ***AFP*** | CTTTGGGCTGCTCGCTATGA | GCATGTTGATTTAACAAGCTGCT |
| ***SNAIL1*** | TCGGAAGCCTAACTACAGCGA | AGATGAGCATTGGCAGCGAG |
| ***VIM*** | GACGCCATCAACACCGAGTT | CTTTGTCGTTGGTTAGCTGGT |
| ***E-CAD*** | CGAGAGCTACACGTTCACGG | GGGTGTCGAGGGAAAAATAGG |
| ***OCT-4*** | CTTGAATCCCGAATGGAAAGGG | GTGTATATCCCAGGGTGATCCTC |
| ***CFTR*** | AAAAGGCCAGCGTTGTCTCC | AAACATCGCCGAAGGGCATTA |
| ***PCNA*** | CCTGCTGGGATATTAGCTCCA | CAGCGGTAGGTGTCGAAGC |
| ***C-MYC*** | GGCTCCTGGCAAAAGGTCA | CTGCGTAGTTGTGCTGATGT |
| ***C-FOS*** | CCGGGGATAGCCTCTCTTACT | CCAGGTCCGTGCAGAAGTC |
| ***FOSL1*** | CAGGCGGAGACTGACAAACTG | TCCTTCCGGGATTTTGCAGAT |
| ***EPCAM*** | AATCGTCAATGCCAGTGTACTT | TCTCATCGCAGTCAGGATCATAA |
| ***RPS6KA2*** | GAAGAAGGCGTCGTGAAGGAG | CCGAACTTTTAGGGTGGCTTT |
| ***C-JUN*** | TCCAAGTGCCGAAAAAGGAAG | CGAGTTCTGAGCTTTCAAGGT |
| ***GAPDH*** | AAAGCGGCTGTTAGTCACTGG | CGAGTCATTGCATACTGTCCAT |
| ***LGR5*** | CTCCCAGGTCTGGTGTGTTG | GAGGTCTAGGTAGGAGGTGAAG |
| ***OLFM4*** | ACCTTTCCCGTGGACAGAGT | TGGACATATTCCCTCACTTTGGA |
| ***CDCA7*** | GGGTGGCGATGAAGTTTCCA | GGGGATGTCTTCCACGGAAC |
| ***SOX9*** | AGCGAACGCACATCAAGAC | CTGTAGGCGATCTGTTGGGG |
| ***UBE2C*** | GACCTGAGGTATAAGCTCTCGC | TTACCCTGGGTGTCCACGTT |
| ***KI67*** | ACGCCTGGTTACTATCAAAAGG | CAGACCCATTTACTTGTGTTGGA |
| ***BIRC5*** | AGGACCACCGCATCTCTACAT | AAGTCTGGCTCGTTCTCAGTG |
| ***CENPA*** | TTCCTCCCATCAACACAGTCG | CACACCACGAGTGAATTTAACAC |
| ***FABP1*** | ATGAGTTTCTCCGGCAAGTACC | CTCTTCCGGCAGACCGATTG |
| ***RBP2*** | TTTTGCCACCCGCAAGATTG | CGGAATGTGCTAGTGGTTTTTGT |
| ***APOA1*** | CCCTGGGATCGAGTGAAGGA | CTGGGACACATAGTCTCTGCC |
| ***APOA4*** | CCCAGCAACTCAATGCCCT | CCTTCAGTTTCTCCGAGTCCT |
| ***VILLIN1*** | CTGAGCGCCCAAGTCAAAG | AGCAGTCACCATCGAAGAAGC |
| ***F3*** | GGCGCTTCAGGCACTACAA | TTGATTGACGGGTTTGGGTTC |
| ***S100A6*** | GGGAGGGTGACAAGCACAC | AGCTTCGAGCCAATGGTGAG |
| ***ANXA3*** | TTAGCCCATCAGTGGATGCTG | CTGTGCATTTGACCTCTCAGT |
| ***ANG4*** | CAAGAAAGGGCTAACGCCTC | GGCTTCGTTGCATTGGACA |
| ***MMP7*** | GAGTGAGCTACAGTGGGAACA | CTATGACGCGGGAGTTTAACAT |
| ***ACTN1*** | TCCATCGGAGCCGAAGAAATC | GTGTCGGTGGATCAAAGCACA |
| ***DEFA6*** | CTGAGCCACTCCAAGCTGAG | GTTGAGCCCAAAGCTCTAAGAC |
| ***LYZ*** | CTTGTCCTCCTTTCTGTTACGG | CCCCTGTAGCCATCCATTCC |
| ***DEFA3*** | TGAGAGCAAAGGAGAATGAG | GCAGAATGCCCAGAGTCTTC |
| ***DEFA4*** | CCTTTGCATGGGATAAAAGCTCT | ACACCACCAATGAGGCAGTTC |
| ***DEFA1*** | TCCCTTGCATGGGACGAAAG | GGTTCCATAGCGACGTTCTCC |
| ***MUC2*** | GAGGGCAGAACCCGAAACC | GGCGAAGTTGTAGTCGCAGAG |
| ***FCGBP*** | GCCAAGGCTGAGATGATAGGC | CCTGCACAGAGATGGCATAGT |
| ***TFF3*** | CCAAGCAAACAATCCAGAGCA | GCTCAGGACTCGCTTCATGG |
| ***AGR2*** | GTCAGCATTCTTGCTCCTTGT | GGGTCGAGAGTCCTTTGTGTC |
| ***KCTD12*** | GCTCGGGCTACATCACCATC | GGTCCCGGCTTTCGTTCAG |
| ***RGS13*** | ACATTGACAGTTCGACAAGAGAG | GAAATCTGGGGTAGGAATCCCT |
| ***CHGA*** | TAAAGGGGATACCGAGGTGATG | TCGGAGTGTCTCAAAACATTCC |
| ***CHGB*** | CAGCCAACGCTGCTTCTCA | GGTTCCTGTTATCCACTGGCA |
| ***TAC1*** | TGATCTGAATTACTGGTCCGACT | TCCGGCAGTTCCTCCTTGA |
| ***SCT*** | GGACGCAGAGAACAGCAT | TGACGGGCAGAGCAGACC |

**Table S2. The sequence of miRNAs primers used in this article.**

| **Gene** | **Forward primer (5’-3’)** | **Reverse primer (5’-3’)** |
| --- | --- | --- |
| ***MiR-192-5p*** | CTGACCTATGAATTGACAGCCAA | GCTGTCAACGATACGCTACGTAAC |
| ***MiR-191-5p*** | GGAATCCCAAAAGCAGCTGA | GCTGTCAACGATACGCTACGTAAC |
| ***MiR-194-5p*** | CTGTAACAGCAACTCCATGTGGA | GCTGTCAACGATACGCTACGTAAC |
| ***MiR-182-5p*** | TTGGCAATGGTAGAACTCACACTA | GCTGTCAACGATACGCTACGTAAC |
| ***MiR-183-5p*** | GCTATGGCACTGGTAGAATTCACT | GCTGTCAACGATACGCTACGTAAC |
| ***MiR-200b-3p*** | CGTAATACTGCCTGGTAATGATGA | GCTGTCAACGATACGCTACGTAAC |
| ***MiR-215-5p*** | CGATGACCTATGAATTGACAGACA | GCTGTCAACGATACGCTACGTAAC |
| ***MiR-371a-5p*** | ACTCAAACTGTGGGGGCACTAA | GCTGTCAACGATACGCTACGTAAC |
| ***MiR-372-3p*** | AAAGTGCTGCGACATTTGAGC | GCTGTCAACGATACGCTACGTAAC |
| ***MiR-373-3p*** | GTGCTTCGATTTTGGGGTGTA | GCTGTCAACGATACGCTACGTAAC |
| ***MiR-141-3p*** | GCCGTAACACTGTCTGGTAAAGAT | GCTGTCAACGATACGCTACGTAAC |
| ***U6*** | ATTCGTGAAGCGTTCCATATTTT | GCTGTCAACGATACGCTACGTAAC |

**Table S3. The sequence of miRNAs mimics and inhibitors used in this article.**

| **Oligonucleotides** | **Sense (5’-3’)** | **Antisense (5’-3’)** |
| --- | --- | --- |
| ***MiR-192-5p-M***^a)^ | CUGACCUAUGAAUUGACAGCC | CUGUCAAUUCAUAGGUCAGUU |
| ***MiR-191-5p-M*** | CAACGGAAUCCCAAAAGCAGCUG | GCUGCUUUUGGGAUUCCGUUGUU |
| ***MiR-182-5p-M*** | UUUGGCAAUGGUAGAACUCACACU | UGUGAGUUCUACCAUUGCCAAAUU |
| ***MiR-183-5p-M*** | UAUGGCACUGGUAGAAUUCACU | UGAAUUCUACCAGUGCCAUAUU |
| ***MiR-200b-3p-M*** | UAAUACUGCCUGGUAAUGAUGA | AUCAUUACCAGGCAGUAUUAUU |
| ***MiR-215-5p-M*** | AUGACCUAUGAAUUGACAGAC | CUGUCAAUUCAUAGGUCAUUU |
| ***MiR-371a-5p-M*** | ACUCAAACUGUGGGGGCACU | UGCCCCCACAGUUUGAGUUU |
| ***MiR-372-3p-M*** | AAAGUGCUGCGACAUUUGAGCGU | GCUCAAAUGUCGCAGCACUUUUU |
| ***MiR-373-3p-M*** | GAAGUGCUUCGAUUUUGGGGUGU | ACCCCAAAAUCGAAGCACUUCUU |
| ***MiR-141-3p-M*** | UAACACUGUCUGGUAAAGAUGG | AUCUUUACCAGACAGUGUUAUU |
| ***MiR-182-5p-I***^b)^ | AGUGUGAGUUCUACCAUUGCCAAA |  |
| ***MiR-194-5p-I*** | UCCACAUGGAGUUGCUGUUACA |  |
| ***MiR-200b-3p-I*** | UCAUCAUUACCAGGCAGUAUUA |  |
| ***MiR-371a-5p-I*** | AGUGCCCCCACAGUUUGAGU |  |
| ***MiR-372-3p-I*** | ACGCUCAAAUGUCGCAGCACUUU |  |
| ***MiR-373-3p-I*** | ACACCCCAAAAUCGAAGCACUUC |  |

^a)^ ((mimic)); ^b)^ ((inhibitor)).

**Table S4. The sequence of RPS6KA2 siRNAs and sgRNAs used in this article.**

| **Oligonucleotides** | **Sense (5’-3’)** | **Antisense (5’-3’)** |
| --- | --- | --- |
| ***SiRNA-RPS6KA2-1*** | CGAUAUCUGACGCAGCUAA | UUAGCUGCGUCAGAUAUCG |
| ***SiRNA-RPS6KA2-2*** | GCAAGCGAUGUGUGCAUAA | UUAUGCACACAUCGCUUGC |
| ***SiRNA-RPS6KA2-3*** | CACCUGUUUAGAGGAUUCA | UGAAUCCUCUAAACAGGUG |
| ***SgRNA-RPS6KA2-1*** | CACCGGGCTTGGCAGGGAGGGGACGGTTT |  |
| ***SgRNA-RPS6KA2-2*** | CACCGGTTCCCACCCACCACCAGCAGTTT |  |

**Table S5. Information of donors of intestinal and liver tissue specimens.**

| **Organoid species** | **Age (Y)** | **Gender** | **Diagnosis** | **Sampling site** |
| --- | --- | --- | --- | --- |
| Int-Orgs | 58 | Male | Gastric adenocarcinoma | Duodenum |
| HPC-Orgs | 56 | Male | 1.Hepatitis B cirrhosis  2.Hepatocellular carcinoma | Para-carcinoma  Liver fibrosis tissue |
| PHHs | 33 | Male | Hepatic hemangioma | Para-tumoral  Normal liver tissue |
| PHBDs | 44 | Male | Hepatic hemangioma | Para-tumoral  Normal liver tissue |

**Table S6. The antibodies information of this article.**

| **Antibodies** | **Species** | **Sources** | **Dilution Ratio** | **Identifier** |
| --- | --- | --- | --- | --- |
| **Anti-Human MUC2** | Mouse | Santa Cruz | 1:50(IF) | Cat#sc-515032 |
| **Anti-Human Villin** | Mouse | Santa Cruz | 1:50(IF) | Cat#sc-374350 |
| **Anti-Human Ki-67** | Mouse | CST | 1:500(IHC) | Cat#9449 |
| **Anti-Human L-FABP** | Mouse | Santa Cruz | 1:50(IF) | Cat#sc-271591 |
| **Anti-Human SOX9** | Mouse | Santa Cruz | 1:50(IF) | Cat#sc-166505 |
| **Anti-Human Lysozyme** | Rabbit | Proteintech | 1:200(IF) | Cat#15013-1AP |
| **Anti-Human E-CAD** | Rabbit | Abcam | 1:500(IF) | Cat#ab40772 |
| **Anti-Human E-CAD** | Mouse | Santa Cruz | 1:100(IF) | Cat#sc-8462 |
| **Anti-Human ChrA** | Mouse | Santa Cruz | 1:50(IF) | Cat#sc-393941 |
| **Anti-Human KRT-20** | Mouse | Santa Cruz | 1:100(IF) | Cat#sc-271183 |
| **Anti-Human LGR5** | Rabbit | Affinity | 1:200(IF) | Cat#DF2816 |
| **Anti-Human VE-CAD** | Rabbit | CST | 1:400(IF) | Cat#2500 |
| **Anti-Human CD31** | Mouse | Santa Cruz | 1:50(IF) | Cat#sc-376764 |
| **Anti-Human ZO-1** | Rabbit | Abcam | 1:200(IF) | Cat#ab221547 |
| **Anti-Human KRT18** | Mouse | CST | 1:400(IF/IHC/FC) | Cat#4548 |
| **Anti-Human ALB** | Rabbit | Abcam | 1:200(IF/IHC) 1:100(FC) | Cat#ab207327 |
| **Anti-Human ALB(FITC)** | Rabbit | Thermofisher | 1:100(FC) | Cat#MA5-46869 |
| **Anti-Human A1AT** | Mouse | Santa Cruz | 1:50(IF/IHC/FC) | Cat#sc-166018 |
| **Anti-Human AFP** | Rabbit | Abcam | 1:200(IF/IHC) | Cat#ab285388 |
| **Anti-Human HNF4A** | Mouse | Santa Cruz | 1:50(IF/FC) | Cat#sc-374229 |
| **Anti-Human HNF4A** | Rabbit | Abcam | 1:1000(FC) | Cat#ab201460 |
| **Anti-Human CYP3A4** | Mouse | Santa Cruz | 1:50(IF) | Cat#sc-53850 |
| **Anti-Human BSEP** | Mouse | Santa Cruz | 1:50(IF) | Cat#sc-74500 |
| **Anti-Human DPPIV** | Rabbit | Proteintech | 1:200(IF) | Cat#29403-1-AP |
| **Anti-Human MDR1** | Rabbit | Affinity | 1:100(IF) | Cat#AF5185 |
| **Anti-Human RPS6KA2** | Mouse | Santa Cruz | 1:50(IF) | Cat#sc-517283 |
| **Anti-Human RPS6KA2** | Rabbit | Affinity | 1:1000(WB) | Cat#DF8603 |
| **Anti-Human p-ATF4(phospho S245)** | Rabbit | Abcam | 1:100(IF) | Cat#ab-28830 |
| **Anti-Human EPCAM** | Mouse | Santa Cruz | 1:200(IF) 1:50(FC) | Cat#sc-25308 |
| **Anti-Human CK19** | Rabbit | Abclone | 1:200(IF) | Cat#A19040 |
| **Anti-Human TSG101** | Rabbit | Abcam | 1:1000(WB) | Cat#ab125011 |
| **Anti-Human HSP70** | Rabbit | Abcam | 1:1000(WB) | Cat#ab2787 |
| **Anti-Human CD63** | Mouse | Santa Cruz | 1:200(WB) | Cat#sc-5257 |
| **Anti-Human Calnexin** | Rabbit | Proteintech | 1:500(WB) | Cat#10427-2-AP |
| **Anti-Mouse Alexa Fluor 488/555/594** | Goat | Abcam | 1:500(IF) 1:200(FC) | Cat#ab150113/ab150118 /ab150116 |
| **Anti-Mouse IgG2a heavy chain (CY7)** | Goat | Abcam | 1:200(FC) | Cat#ab130787 |
| **Anti-Mouse IgG H&L (DyLight® 650)** | Goat | Abcam | 1:200(FC) | Cat#ab96882 |
| **Anti-Rabbit Alexa Fluor 488/555/594/647** | Goat | Abcam | 1:500(IF) 1:200(FC) | Cat#ab150077/ab150078 /ab150080/ab150115 |
| **Anti-Mouse IgG (H+L) HRP** | Goat | Affinity | 1:3000(WB) | Cat#S0002 |
| **Anti-Rabbit IgG (H+L) HRP** | Goat | Affinity | 1:3000(WB) | Cat#S0001 |
